# Synthesis and biological evaluation of thieno[3,2-*c*]pyrazol-3-amine derivatives as potent glycogen synthase kinase 3β inhibitors for Alzheimer’s disease

**DOI:** 10.1080/14756366.2022.2086867

**Published:** 2022-06-14

**Authors:** Ning Yan, Xiao-Long Shi, Long-Qian Tang, De-Feng Wang, Xun Li, Chao Liu, Zhao-Peng Liu

**Affiliations:** a Institute of Medicinal Chemistry, Key Laboratory of Chemical Biology (Ministry of Education), School of Pharmaceutical Sciences, Shandong University, Jinan, PR China; b Institute of Materia Medica, Shandong First Medical University & Shandong Academy of Medical Sciences, Jinan, PR China

**Keywords:** Alzheimer’s disease, GSK-3β inhibitors, Aβ, tau hyperphosphorylation, neurite outgrowth

## Abstract

Glycogen synthase kinase 3β (GSK-3β) catalyses the hyperphosphorylation of tau protein in the Alzheimer’s disease (AD) pathology. A series of novel thieno[3,2-*c*]pyrazol-3-amine derivatives were designed and synthesised and evaluated as potential GSK-3β inhibitors by structure-guided drug rational design approach. The thieno[3,2-*c*]pyrazol-3-amine derivative **16b** was identified as a potent GSK-3β inhibitor with an IC_50_ of 3.1 nM *in vitro* and showed accepted kinase selectivity. In cell levels, **16b** showed no toxicity on the viability of SH-SY5Y cells at the concentration up to 50 μM and targeted GSK-3β with the increased phosphorylated GSK-3β at Ser9. Western blot analysis indicated that **16b** decreased the phosphorylated tau at Ser396 in a dose-dependent way. Moreover, **16b** effectively increased expressions of β-catenin as well as the GAP43, N-myc, and MAP-2, and promoted the differentiated neuronal neurite outgrowth. Therefore, the thieno[3,2-*c*]pyrazol-3-amine derivative **16b** could serve as a promising GSK-3β inhibitor for the treatment of AD.

## 1. Introduction

**Figure 1.**
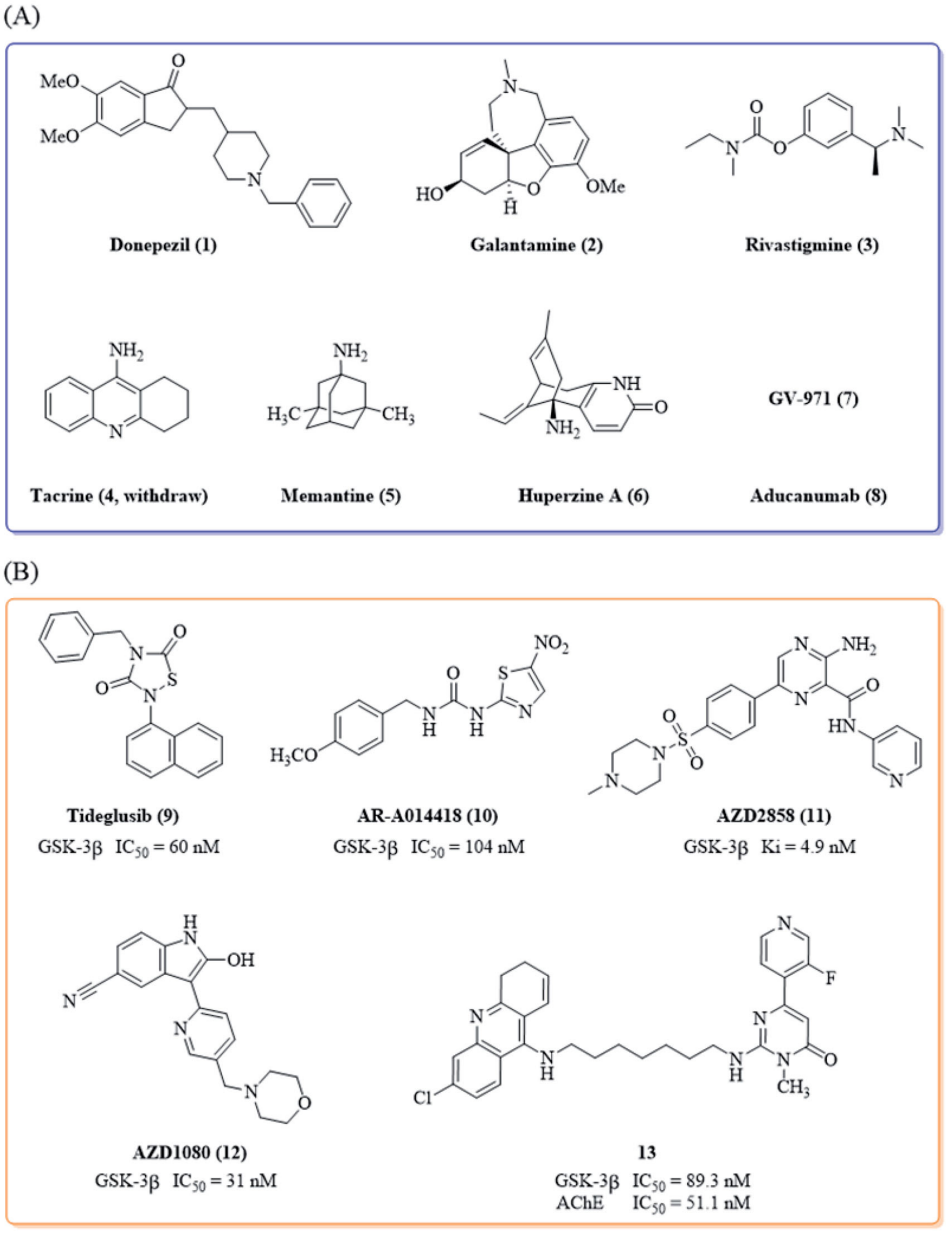
(A) Drugs approved by FDA and NMPA for the treatment of AD by June 2021; (B) Representative GSK-3β inhibitors with anti-AD activity *in vivo*.

Alzheimer’s disease (AD), characterised by memory loss and cognitive impairments, is a chronic neurodegenerative disorder that disturbs more than 50 million people’s healthy life worldwide^1^^,^^2^. At present, only a few drugs, donepezil, galantamine, rivastigmine, tacrine, memantine, huperzine A (NMPA), GV-971 (NMPA) and aducanumab (Figure 1(A)), are available for the treatment of this disease^3–6^; however, there are no drugs that can effectively block or reverse the progression of AD, possibly due to the complicated aetiology of this disease. A number of hypotheses have been proposed for AD pathogenesis^7–18^, among which, the β-amyloid (Aβ) deposit and tau protein hyperphosphorylation are the key concerns^19^^,^^20^. The coexistence of Aβ plaques and tau intracellular neurofibrillary tangles (NFTs) in the neocortex is associated with the collapse of neural circuits and cognitive decline, and the interactions between Aβ and tau exaggerate the pathology of AD^21^^,^^22^.

Glycogen synthase kinase-3 (GSK-3), a proline-directed serine/threonine kinase, is closely associated with Aβ deposits and tau hyperphosphorylation. GSK-3 has two subtypes, GSK-3α (51 kDa) and GSK-3β (47 kDa) in mammals. Most notably, in the brain, GSK-3β is the primary isoform and acts as the dominator for tau hyperphosphorylation^23^^,^^24^. The overactivation of GSK-3β was identified and co-localized with neurofibrillary tangles (NFTs) in postmortem AD brain^25^^,^^26^. Hyperphosphorylated tau lost the physiological ability to bind to tubulin, and therefore, detached from tubulin, resulting in the formation of paired helical filaments (PHFs) and subsequently aggregated to NFTs^27^^,^^28^. The abnormal deposition of NFTs led to extensive damage to the normal transport and signalling pathways, cell cytoskeleton, mitochondria, and neuronal cell death^29^. In addition to the tau pathway, GSK-3β could promote the Aβ fibril generation and induce Aβ aggregation^30^. In transgenic AD mice, the inhibition of GSK-3β could reduce the Aβ-induced toxicity and improve cognition performances^31^. Moreover, the overactivation of GSK-3β could cause neuroinflammation, neuronal death, and apoptosis^32^^,^^33^. In the light of the multifunctional roles of GSK-3β in AD pathology, GSK-3β becomes a potential target for the development of anti-AD drugs^34^^,^^35^.

**Figure 2.**
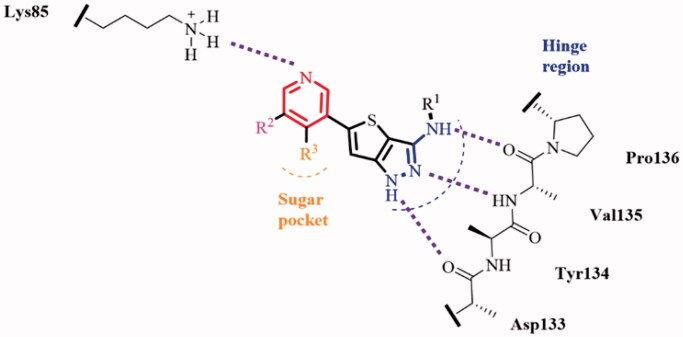
Design of thieno[3,2-*c*]pyrazol-3-amine derivatives as GSK-3β inhibitors.

Tideglusib (Figure 1(B)) is the small thiadiazolidinone GSK-3β inhibitor that entered the clinical trial for the treatment of AD^36^. Besides, the GSK-3β inhibitors, AR-A014418, AZD2858, AZD1080, as well as the GSK-3β and acetylcholinesterase (AchE) dual inhibitor **13**, demonstrated anti-AD effects in AD animals^26^^,^^37–39^ (Figure 1(B)). Most of the reported GSK-3β inhibitors feature with the “double-sites occupation” pharmacophore model: a key skeleton interacted with the hinge region by forming two hydrogen bonds with Asp133 and/or Val135, a moiety connected to the key skeleton as hydrogen bond acceptor to interact with Lys85 side chain^40^. Following this model, we used the thieno[3,2-*c*]pyrazol-3-amine as the key framework and designed a series of thieno[3,2-*c*]pyrazol-3-amine derivatives as the potential GSK-3β inhibitors. The thieno[3,2-*c*]pyrazol-3-amine framework has the possibility to form triple hydrogen bonds with the hinge region so as to enhance its binding with the enzyme (Figure 2). The N atom of pyridine moiety connected with the thieno[3,2-*c*]pyrazol-3-amine may act as hydrogen bond acceptor to interact with Lys85 side chain. A variety of the substituents (R^1^, R^2^ and R^3^) were introduced to investigate their effects on the GSK-3β inhibitory activities.

## 2. Results and discussion

### 2.1. Chemistry

The newly designed thieno[3,2-*c*]pyrazol-3-amine derivatives were synthesised as outlined in Scheme 1. The 5-bromo-1*H*-thieno[3,2-*c*]pyrazol-3-amine **14** was prepared according to the reported method from 3-bromothiophene in seven steps^41^. The reaction of **14** with acyl chloride or sulphonyl chloride generated the thieno[3,2-*c*]pyrazol-3-amine intermediates **15a**–**15e**. The Suzuki coupling of **15a**–**15e** with pyridylboronic acids or pyridylboronic acid esters provided the thieno[3,2-*c*]pyrazol-3-amine derivatives **16a**–**16e**, **17a**–**17d** and **18a**–**18n** in 11.5–55.9% yields. A total of 23 thieno[3,2-*c*]pyrazol-3-amine derivatives were prepared.

**Figure:**
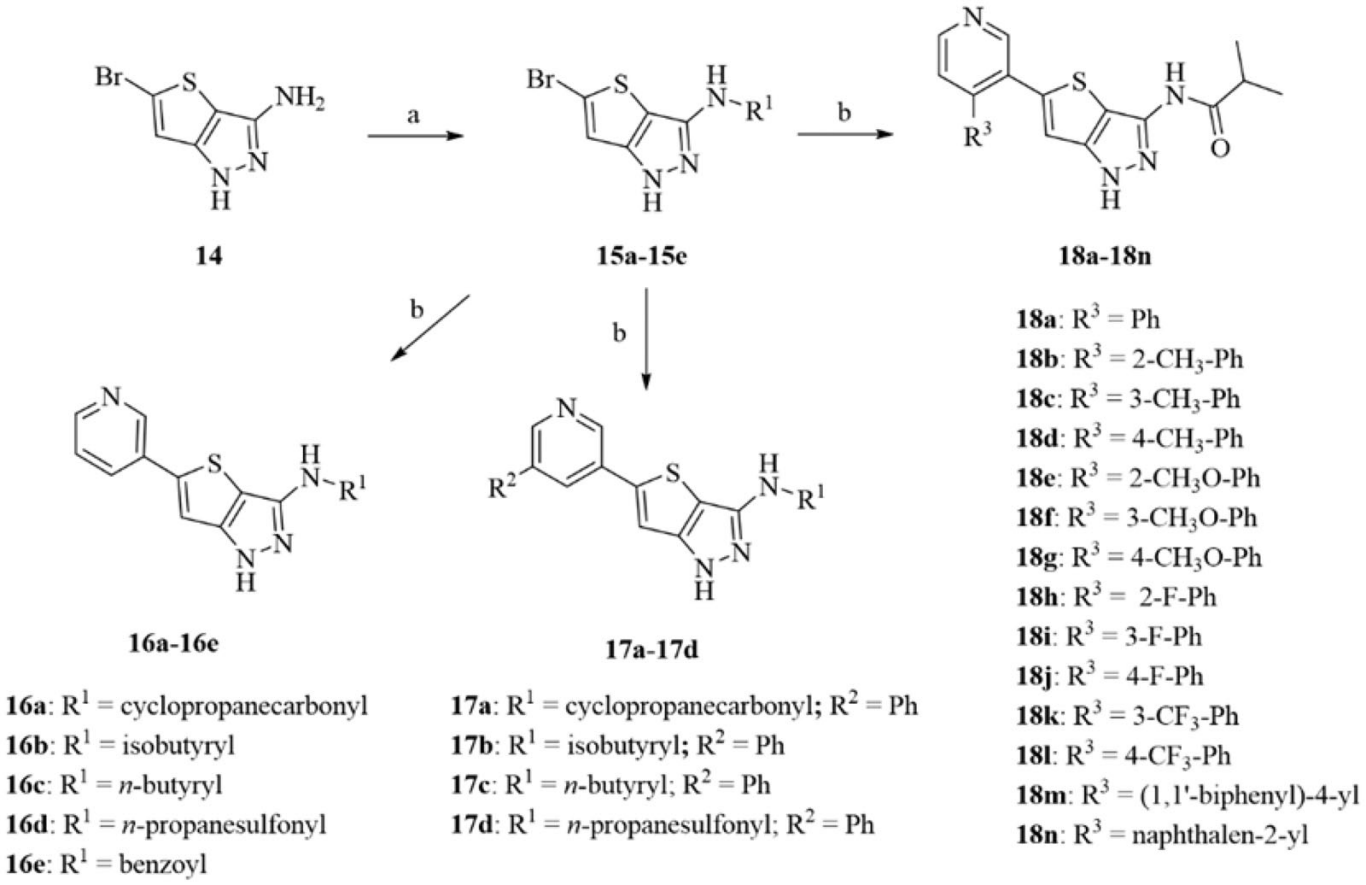
**Scheme 1**. Synthesis of thieno[3,2-*c*]pyrazol-3-amine derivatives **16a**–**16e**, **17a**–**17d** and **18a**–**18n**. Reagents and conditions: (a) various acyl chlorides or propane-1-sulphonyl chloride, pyridine, 110 °C; (b) substituted arylboronic acids or arylboronic acid esters, Pd(dppf)Cl_2_, CH_3_CO_2_K, DMF/EtOH/H_2_O or DMF/1,4-dioxane/H_2_O, 100 °C.

### 2.2. GSK-3β inhibitory activity and kinase selectivity

All the targeted compounds were evaluated for their GSK-3β inhibitory activities in the calliper mobility shift assay *in vitro*. AR-A014418 (**10**, Figure 1(B)), a prototypical GSK-3β-specific inhibitor, was used as the positive control^37^.

At first, the effects of the acyl or sulphonyl groups at the thieno[3,2-*c*]pyrazol-3-amine on GSK-3β inhibitory activities were investigated. As shown in Scheme 1 and Table 1, the cyclopropanecarbonyl and the isobytyryl group showed similar effects on the GSK-3β inhibitory potency. Compounds **16a** and **16b** were very potent GSK-3β inhibitors with the IC_50_ values of 4.4 nM and 3.1 nM, respectively. When the thieno[3,2-*c*]pyrazol-3-amine was substituted by the *n*-butyryl (**16c**) or benzoyl (**16e**), the resulting compound **16c** or benzoyl **16e** maintained high potency, but was about 10-fold less active than **16b**. However, the sulphonamide **16d** showed very weak GSK-3β inhibitory activity. The introduction of a phenyl group at the *meta*-position of the pyridine ring in **16a**–**16d** showed subtle influences on the activity of their parent compounds. Compounds **17a** and **17b** were about 4-fold less active than **16a** and **16b**, but compound **17c** was active as that of **16c**. The sulphonamide **17d** was not active. Therefore, the substitution of the thieno[3,2-*c*]pyrazol-3-amine with a sulphonyl group was not preferred.

**Table 1.** Inhibitory effects of compounds against GSK-3β.

Compd.	GSK-3β IC_50_ (nM)^a^	Compd.	GSK-3β IC_50_ (nM)^a^
**16a**	4.4 ± 0.2	**18d**	99 ± 4.8
**16b**	3.1 ± 0.1	**18e**	64 ± 2.7
**16c**	36 ± 1.7	**18f**	113 ± 8.6
**16d**	1394 ± 55.2	**18g**	105 ± 5.2
**16e**	33 ± 1.6	**18h**	94 ± 3.5
**17a**	20 ± 1.1	**18i**	173 ± 6.8
**17b**	14 ± 0.6	**18j**	15 ± 0.3
**17c**	37 ± 1.3	**18k**	107 ± 6.1
**17d**	>5000	**18l**	158 ± 8.9
**18a**	84 ± 3.2	**18m**	387 ± 21.2
**18b**	64 ± 2.3	**18n**	195 ± 10.4
**18c**	153 ± 6.6	AR-A014418	138.8 ± 7.9

^a^The IC_50_ values are shown as the mean ± SD from two separate experiments.

As compound **16b** showed very potent GSK-3β inhibitory activity, further structural modifications based on **16b** were made at the *para*-position of the pyridine ring. In general, inducing a phenyl or substituted phenyl, a biphenyl, a naphthalenyl group at this position decreased the potency of **16b**. The phenyl substituted analogue **18a** showed modest GSK-3β inhibitory activity with the IC_50_ of 84 nM. For the methyl or methoxy substituted phenyl derivatives, the activity was in the order of *ortho*- > *para*- > *meta*-. The *ortho*-methyl and the *ortho*-methoxy derivatives **18b** and **18e** were slightly more active than **18a**, with IC_50_ values of 64 and 64 nM, respectively. For the fluorine substituted phenyl analogues, the 4-F-phenyl derivative **18j** was highly active, with an IC_50_ of 15 nM, possibly due to the special features of fluorine atom with the smallest size and the largest electron-withdrawing property. The trifluoromethyl group (**18k** and **18l**) also decreased the potency of **18a** slightly. The biphenyl derivative **18m** showed much weak activity with an IC_50_ of 387 nM. The naphthalenyl derivative **18n** was about 2-fold less active than **18a**.

**Figure 3.**
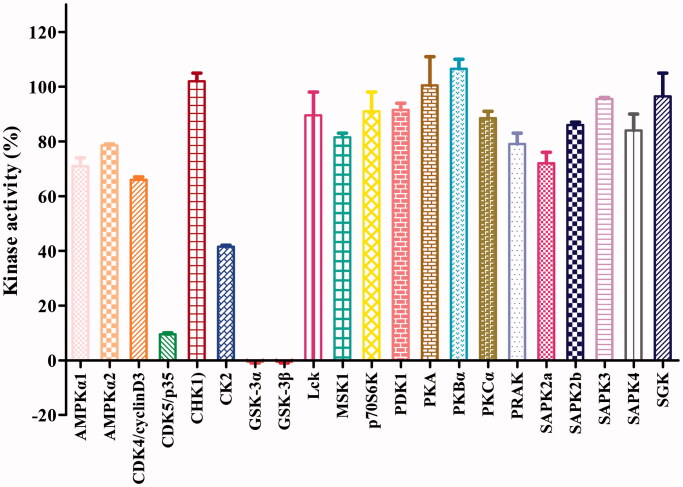
Effects of compound **16b** on the activities of 21 protein kinases *in vitro*. Protein kinases were of human origin and assayed in the presence of 1.0 μM compound **16b** or vehicle (DMSO). The enzymatic activity was measured in the presence of *K*_m_ ATP. Kinase activities were given as the mean of twice determinations. AMPKα1, AMP-activated protein kinase 1; AMPKα2, AMP-activated protein kinase 2; CDK4/cyclinD3, cyclin-dependent protein kinase-4/cyclinD3; CDK5/p35, cyclin-dependent protein kinase-5/p35; CHK1, checkpoint kinase-1; CK2, casein kinase-2; Lck, lymphocyte kinase; MSK1, mitogen- and stress-activated protein kinase-1; p70S6K, p70 ribosomal protein S6 kinase; PDK1, 3-phosphoinositide-dependent protein kinase-1; PKA, cAMP-dependent protein kinase; PKBα, protein kinase Bα; PKCα, protein kinase Cα; PRAK, p38-regulated/activated kinase; SAPK2a, stress-activated protein kinase-2a; SAPK2b, stress-activated protein kinase-2b; SAPK3, stress-activated protein kinase-3; SAPK4, stress-activated protein kinase-4; SGK, serum- and glucocorticoid-induced protein kinase.

The potent GSK-3β inhibitor **16b** was next subjected to kinase selectivity assay. A panel of kinases which were structurally related to GSK-3β was used for the GSK-3β selectivity studies^26^^,^^37^^,^^38^. Among a panel of 21 diverse kinases, at the concentration of 1.0 μM (>320-fold IC_50_ value on GSK-3β), **16b** showed in general good selectivity over most of the kinases except for the low selectivity over the GSK-3α and CDK5 and moderate selectivity over CK2 (Figure 3). GSK-3α and GSK-3β are known to share a 98% sequence homology at the catalytic site^42^. CDK5 and GSK-3 belong to the CMGC protein kinase family, which shared highly homology with each other^43^^,^^44^. CDK5 is known abnormally activated and also responsible for the tau hyperphosphorylation in AD^45^. It was known that Aβ could increase CK2 activity, which in turn accelerated the tau phosphorylation in AD^46^. Therefore, the inhibition of CDK5 and CK2 by **16b** may be beneficial for its anti-AD activities.

### 2.3. Molecular docking study

**Figure 4.**
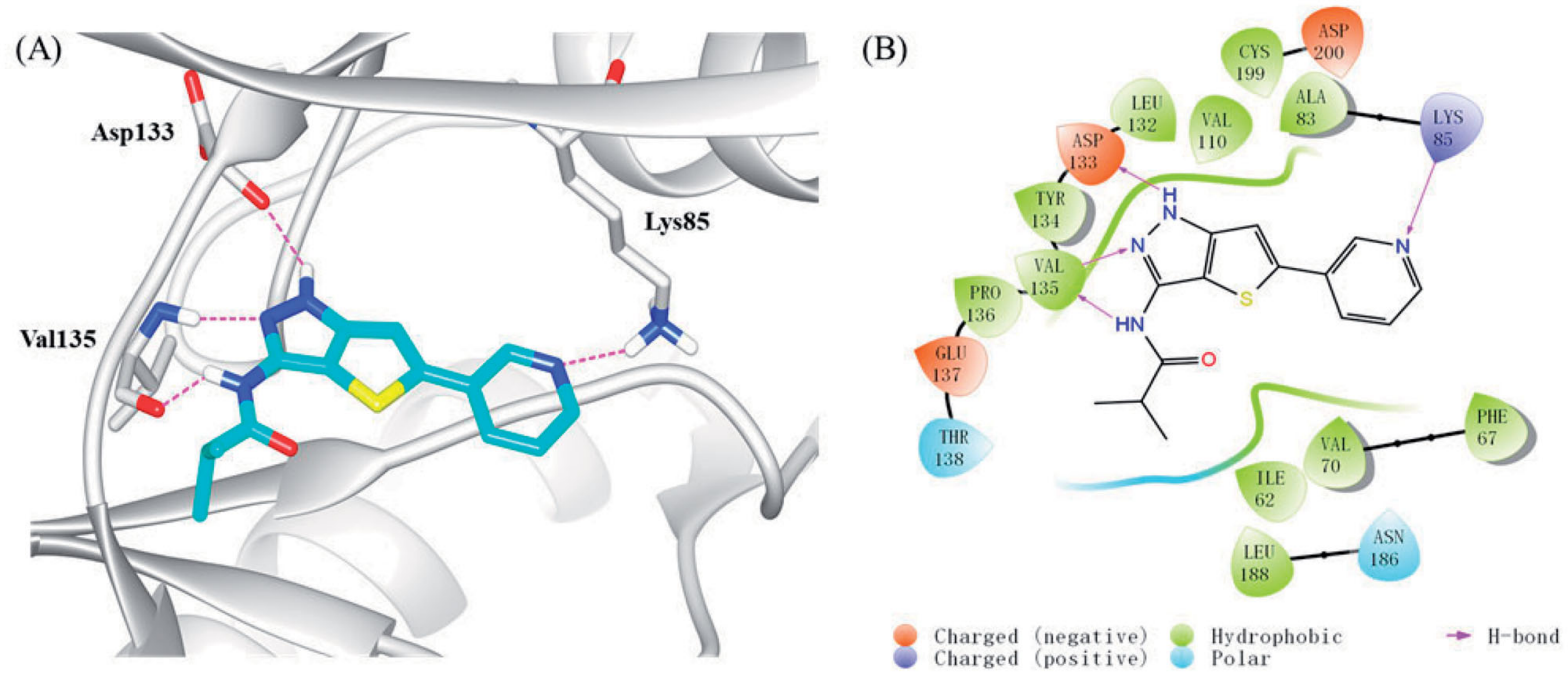
(A) Docking model of compound **16b** in the ATP binding pocket of GSK-3β (PDB: 4ACG). Compound **16b** was shown in green colour stick model, and hydrogen-bonding interactions were shown as purple dotted lines; (B) 2D interactions diagram of **16b**. For clarity, only the polar hydrogen atoms were shown.

To investigate the possible binding mode of compound **16b** with GSK-3β, molecular docking was performed on GSK-3β (PDB: 4ACG)^38^ using Sybyl-X 2.0 softsuite. All docked conformations were ranked based on docking scores. As depicted in Figure 4(A and B), compound **16b** fitted well into the ATP binding pocket of GSK-3β. The thieno[3,2-*c*]pyrazol-3-amine skeleton occupied the adenine pocket and formed triple hydrogen bonds with backbone atoms of Asp133 and Val135 in the hinge region, which is necessary for the ligand recognition. In addition to these hydrogen bonds, the thieno[3,2-*c*]pyrazol-3-amine portion also made hydrophobic interactions with the hydrophobic pocket formed by the residues of Ala83, Val110, Leu132, Asp133, Tyr134, Val135, and Leu188. The pyridine ring also participated in hydrophobic interactions with Phe67, Val70, and Cys199. Meanwhile, the N atom of pyridine served as a hydrogen bond acceptor to interact with Lys85 which located at the β-strand of N-terminal domain. Besides, the terminal isobytyryl group located at a position adjacent to the hinge region and produced hydrophobic interactions with Ile62 and Pro136. As expected, **16b** followed the “double-sites occupation” pharmacophore model well that may contribute to its high inhibitory activity with GSK-3β.

### 2.4. Cytotoxicity of compound 16b on SH-SY5Y cells

**Figure 5.**
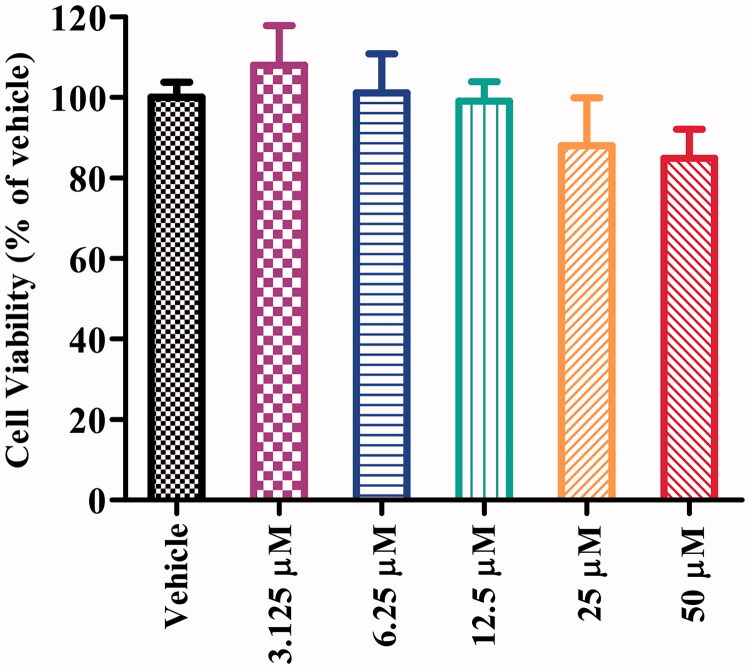
Cell viability of SH-SY5Y cells exposed to compound **16b** at different concentrations (range from 3.125 − 50 μM) for 24 h. Vehicle treater cells were used as control. The results were expressed as the percentage of viable cells observed after treatment with compound **16b** respect to vehicle-treated cells (100%) and shown as the mean ± SD from at least three separate experiments.

To evaluate the neuronal cell cytotoxicity of compound **16b**, we examined its cytotoxic profile on human neuroblastoma SH-SY5Y cells after incubation for 24 h by the MTT (3-(4,5-dimethylthiazol-2-yl)-2,5-diphenyltetrazolium bromide) assay. Differential SH-SY5Y cells possess more neuron-like morphology and biochemical processes to human mature neurons, such as extensively branched neurites and neuro-special markers and were widely used in many neuronal activity studies *in vitro*^47^. As depicted in Figure 5, compound **16b** exhibited no significant cytotoxicity at concentration up to 50 μM, which corresponded to >16000-fold the IC_50_ value of GSK-3β inhibitory activity.

### 2.5. The effects of 16b on GSK-3β and β-catenin expressions in cells

**Figure 6.**
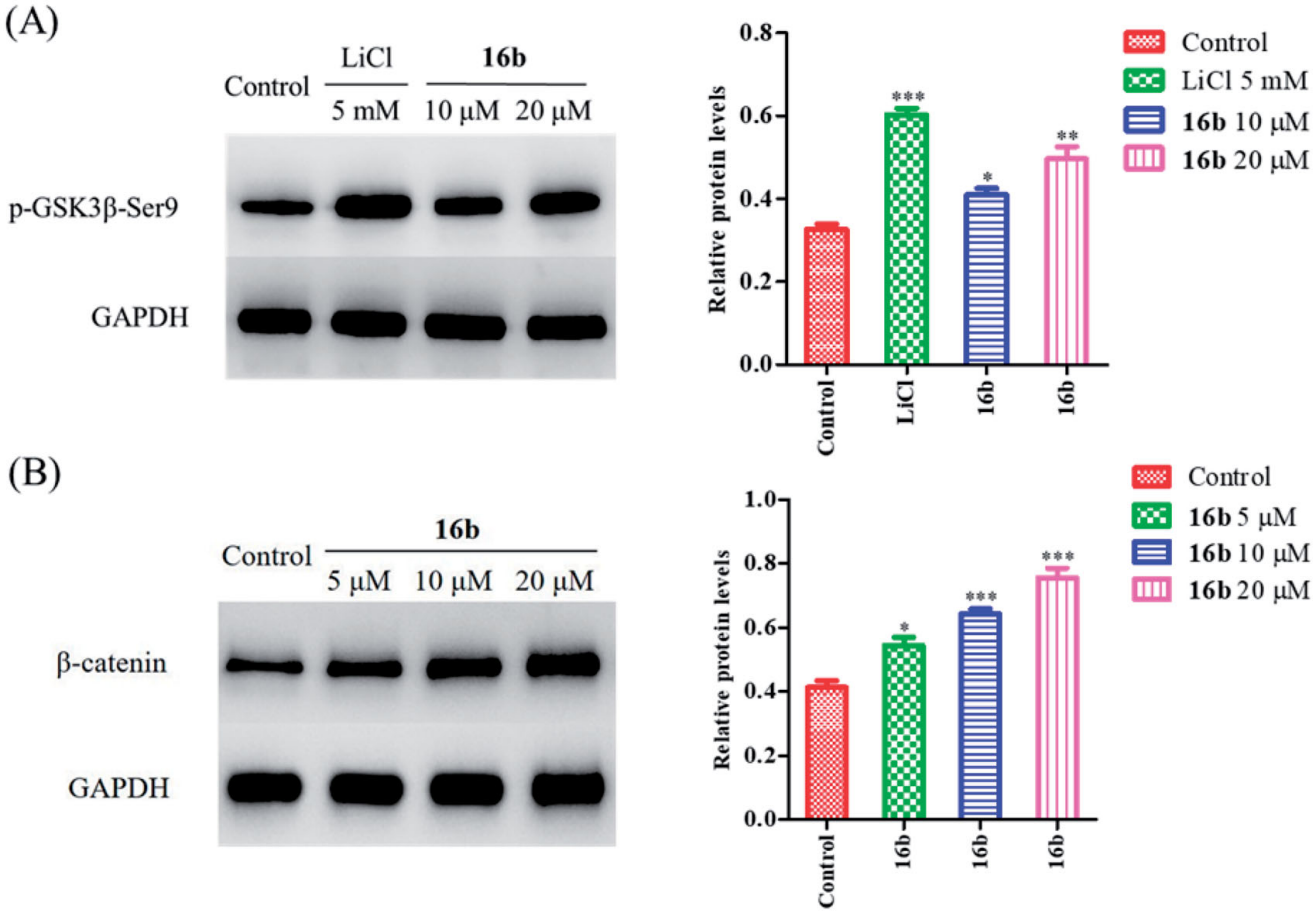
(A) The effect of **16b** on phosphorylation of GSK-3β at Ser9; (B) the effect of **16b** on β-catenin abundance. Protein expressions were detected by immunoblot analysis with a specific antibody. Values are reported as the mean ± SD of three independent experiments. *p < 0.05, **p < 0.01, ***p < 0.001 vs control.

GSK-3β is activated by phosphorylation at Tyr216 and is inhibited by phosphorylation at Ser9 ^48^. As one of the substrates of Akt (protein kinase B), GSK-3 can inhibit Akt^49^. The inhibition of GSK-3β increases the p-Akt expression and activates the PI3K/Akt pathway, and that, in turn, phosphorylates the GSK-3β at the Ser9 site^50^^,^^51^. Since **16b** showed excellent inhibitory potency against GSK-3β *in vitro*, we further investigated its effect on the phosphorylation of GSK-3β at Ser9 on SH-SY5Y cells by Western blot assay, using LiCl as a positive control. As shown in Figure 6(A), LiCl greatly promoted GSK-3β phosphorylation at Ser9 (p-GSK-3β/GAPDH: 0.60 vs 0.33). The treatment with **16b** at 10 μM and 20 μM dose-dependently increased the p-GSK-3β level at Ser9 compared with the control group, with the p-GSK-3β/GAPDH ratio was 0.41 and 0.50, respectively. Therefore, in cellular level, compound **16b** was confirmed to have a direct effect on GSK-3β.

GSK-3β is implicated in the Wnt/β-catenin signalling pathway, which plays an important role in neuronal development^29^. GSK-3β, together with adenomatous polyposis coli (APC), Axin, and casein kinase 1 (CK1), form a ploy-protein complex that regulates the hyperphosphorylation of β-catenin. Phospho-β-catenin is recognised by ubiquitin and degraded by proteasomes^52–54^. Pharmacological inhibition of GSK-3β leads to the activation and stabilisation of β-catenin, subsequently resulting in the accumulation of β-catenin in cytoplasm^55–57^. The activation of Wnt/β-catenin signalling pathway can promote synaptic growth, alleviate spatial memory impairment and neurodegeneration in Alzheimer’s models ^58–60^. Moreover, β-catenin also plays a pivotal role in cell adhesion complexes. The combination of β-catenin and N-cadherin elevates cell-to-cell interactions which is prerequisite for neuronal differentiation^61^^,^^62^. Therefore, we further evaluated the effect of **16b** on β-catenin. In agreement with its GSK-3β inhibitory activity on SH-SY5Y cells, **16b** increased β-catenin abundance in a dose-dependent manner. As shown in Figure 6(B), after treatment with **16b** at the concentration of 5 μM, 10 μM and 20 μM, the β-catenin/GADPH ratio increased from 0.41 of the control to 0.54, 0.64, 0.76, respectively.

### 2.6. Inhibition of Aβ-induced tau protein hyperphosphorylation

**Figure 7.**
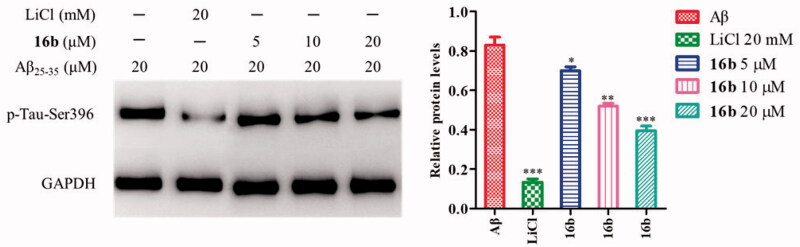
Inhibition of Aβ-induced tau phosphorylation by **16b** in SH-SY5Y cells. Values are reported as the mean ± SD of three independent experiments. *p < 0.05, **p < 0.01, ***p < 0.001 vs control.

GSK-3β phosphorylates tau at sites (Ser199, Ser396, and Ser443), and the hyperphosphorylated tau aggregate into NFTs in AD^63^. Inhibition of GSK-3β could reduce tau hyperphosphorylation. Therefore, the cell-based assay examining Aβ-induced tau phosphorylation at Ser396 represents a direct functional assay to measure the cellular activity of GSK-3β inhibitors^64^. To investigate the effect of **16b** on tau phosphorylation, Western blot analysis was carried out to check Aβ-induced tau protein hyperphosphorylation at Ser396 in SH-SY5Y cells, using LiCl as the control. As shown in Figure 7, the treatment with 20 μM Aβ_25-35_ caused the tau protein hyperphosphorylation at Ser396 with a p-tau/GAPDH ratio of 0.83. At the concentration of 5 μM, 10 μM and 20 μM, **16b** decreased the phosphorylation tau level to 0.70, 0.52, 0.40, respectively.

### 2.7. Effects of 16b on neuronal neurite outgrowth and GAP43, N-myc, and MAP-2 expressions in SH-SY5Y cells

Among kinds of pathological symptoms, neurogenesis impairment and neuronal loss play important roles in neurodegeneration in AD. Therefore, regulating neurogenesis is considered to be a promising therapeutic option for AD^65^.

**Figure 8.**
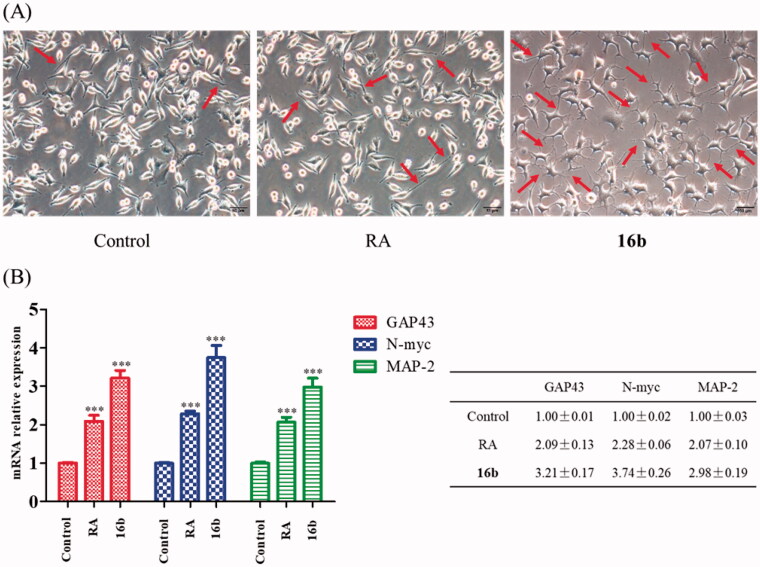
(A) Effect of **16b** (10 μM) on neurite outgrowth (72 h). Red arrows indicated cells bearing neurites. Pictures were taken at 200 × magnification; (B) Effect of **16b** (10 μM) on neurogenesis markers expressions (24 h). ***p < 0.001 vs control.

Compelling evidence indicated that GSK-3β plays a large part in synaptic plasticity and neurogenesis^66^^,^^67^. Differentiated SH-SY5Y cells express neurogenesis-related markers, including growth-associated protein 43 (GAP43), the N-myc gene as well as neuronal polarity marker microtubule-associated protein 2 (MAP-2). GAP43 is an intrinsic determinant of neuronal development and plasticity which regulates axon growth and regeneration^68^^,^^69^. N-myc is indispensable to normal neurogenesis in the expansion of progenitor cell populations^70^. MAP-2 regulates neuronal development, structural stability and synaptic plasticity through the formation of axonal and dendritic processes^71^^,^^72^. Inhibition of GSK-3β could induce neurogenesis and promote the expressions of the neurogenesis-related markers^73^. Firstly, we used SH-SY5Y cells to study the effect of **16b** on neurite outgrowth, using retinoic acid (RA) as a positive control^74^. After incubating with **16b** (10 μM) or RA (10 μM) for 72 h, the morphology of differentiated neuronal neurite outgrowth were obtained. As depicted in Figure 8(A), **16b** exhibited a substantial ability in inducing SH-SY5Y cells neurite outgrowth compared with RA.

We next quantified the neurogenesis-related markers to evaluate the effect of **16b** on neurogenesis. Co-incubated **16b** (10 μM) or RA (10 μM) with SH-SY5Y cells for 24 h, the mRNA expressions of GAP43, N-myc and MAP-2 were measured by the quantitative real-time reverse transcription-PCR (RT-PCR) analysis. As shown in Figure 8(B), RA was able to up-regulate the mRNA expressions of GAP43, N-myc and MAP-2. Meanwhile, compound **16b** exhibited more potent effects on inducing the expressions of GAP43, N-myc and MAP-2 compared with RA.

## 3. Conclusion

In summary, a series of novel thieno[3,2-*c*]pyrazol-3-amine derivatives were designed, synthesised, and evaluated as potential GSK-3β inhibitors. Compound **16b** exhibited potent GSK-3β inhibitory activity with IC_50_ at single-digit nanomolar level. In a panel of 21 kinases, **16b** showed in overall good selectivity over most of them except for the CDK5 and CK2 kinases. In cellular level, **16b** showed no cytotoxicity against SH-SY5Y cells at the concentration up to 50 μM and inhibited GSK-3β through the up-regulation of the phosphorylation at Ser9. Meanwhile, **16b** inhibited the Aβ-induced tau protein hyperphosphorylation at Ser396. In addition, β-catenin plays a crucial role in neurogenesis. Compound **16b** inhibited GSK-3β, interfered the physiological degradation of β-catenin, resulting in the abundance of β-catenin. Moreover, **16b** could increase the mRNA expressions of the recognised neurogenesis-related markers and promote the differentiated neuronal neurite outgrowth, which is very useful in face of progressive neurogenesis impairment and neuronal loss in AD. Compelling evidence have verified GSK-3β to process function link between Aβ and tau. In view of the involvement in multiple pathways in AD progress, GSK-3β is being an interesting drug discovery target for the treatment of AD. As a potent GSK-3β inhibitor, **16b** could serve as a promising lead for further investigation in facing the complicated pathogenesis of AD.

## 4. Experimental section

### 4.1. Chemistry

All solvents used were commercially available and were used without further purification unless otherwise noted. Starting materials used were either available from commercial sources or prepared according to literature procedures. For examined compounds, ^1^H and ^13 ^C nuclear magnetic resonance (NMR) spectra were recorded on a Bruker-400 or Bruker-600 NMR spectrometer, respectively. The following reference signals were used: TMS δ 0.00, or the residual solvent signal of DMSO-*d*_6_ δ 2.50 (^1^H), δ 39.52 (^13 ^C). MS spectra data were obtained using an API 4000 instrument. High-resolution mass spectrometry (HRMS) data were acquired by an AB-Triple TOF5600 or Agilent Q-TOF6540 instrument. Melting points (Mp) were measured on an X-6 micromelting point apparatus (Beijing Tech. Co., Ltd., Beijing, China). The reactions were followed by thin-layer chromatography (TLC) and visualised in an iodine chamber or with a UV lamp. Compounds were purified by column chromatography using silica gel (200 − 300 mesh). The purity (> 95%) of samples were determined using a Shimadzu LC-20AT series system (column, Shim-pack GWS C18, 4.6 × 250 mm, 5 μm; mobile phase, methanol/H_2_O = 80/20; flow rate, 1.0 ml/min; UV wavelength, 254 nm).

#### 4.1.1. N-(5-Bromo-1H-thieno[3,2-c]pyrazol-3-yl)cyclopropanecarboxamide (15a)

To a stirred solution of 5-bromo-1*H*-thieno[3,2-*c*]pyrazol-3-amine (**14**, 218 mg, 1.00 mmol) in pyridine (5 ml) was added dropwise cyclopropanecarbonyl chloride (115 mg, 1.10 mmol). The reaction solution was heated to reflux at 110 °C for 12 h. The reaction was quenched up by the addition of methanol (5 ml), and concentrated under reduced pressure. The residues were purified by silica gel chromatography (hexane/EtOAc = 2:1) to give **15a** (231 mg, 80.8%) as a yellow solid. Mp: > 240 °C. ^1^H NMR (400 MHz, DMSO-*d*_6_) 12.53 (s, 1H), 11.14 (s, 1H), 7.24 (s, 1H), 1.92 − 1.81 (m, 1H), 0.89 − 0.75 (m, 4H); ^13 ^C NMR (100 MHz, DMSO-*d*_6_) δ 171.69, 145.36, 138.95, 120.18, 113.00, 110.42, 13.49, 7.38; ESI (MS): calcd. for C_9_H_9_^81^BrN_3_OS [M + H]^+^: 287.96, found: 288.08.

#### 4.1.2. N-(5-Bromo-1H-thieno[3,2-c]pyrazol-3-yl)isobutyramide (15b)

According to the same procedures for preparing **15a**, compound **15b** was obtained from 5-bromo-1*H*-thieno[3,2-*c*]pyrazol-3-amine (**14**) and isobutyryl chloride in 72.2% yield as a yellow solid. Mp: >240 °C. ^1^H NMR (400 MHz, DMSO-*d*_6_) δ 12.53 (s, 1H), 10.80 (s, 1H), 7.24 (s, 1H), 2.71 − 2.60 (m, 1H), 1.08 (d, *J* = 6.8 Hz, 6H); ^13 ^C NMR (100 MHz, DMSO-*d*_6_) δ 175.20, 145.35, 138.92, 120.18, 113.03, 110.48, 33.77, 19.37; ESI (MS): calcd. for C_9_H_11_BrN_3_OS [M + H]^+^: 287.98, 289.98, found: 288.07, 290.07.

#### 4.1.3. N-(5-Bromo-1H-thieno[3,2-c]pyrazol-3-yl)butyramide (15c)

According to the same procedures for preparing **15a**, compound **15c** was obtained from 5-bromo-1*H*-thieno[3,2-*c*]pyrazol-3-amine (**14**) and butyryl chloride in 76.9% yield as a white solid. Mp: 228 − 230 °C. ^1^H NMR (600 MHz, DMSO-*d*_6_) δ 12.51 (s, 1H), 10.79 (s, 1H), 7.24 (s, 1H), 2.29 (t, *J* = 7.4 Hz, 2H), 1.64 − 1.52 (m, 2H), 0.89 (t, *J* = 7.4 Hz, 3H); ^13 ^C NMR (150 MHz, DMSO-*d*_6_) δ 171.12, 145.34, 138.85, 120.17, 113.00, 110.51, 36.96, 18.38, 13.60; HRMS (ESI): calcd. for C_9_H_11_^81^BrN_3_S [M + H]^+^: 289.9780, found: 289.9789.

#### 4.1.4. N-(5-Bromo-1H-thieno[3,2-c]pyrazol-3-yl)propane-1-sulphonamide (15d)

According to the same procedures for preparing **15a**, compound **15d** was obtained from 5-bromo-1*H*-thieno[3,2-*c*]pyrazol-3-amine (**14**) and 1-propanesulfonyl chloride as a yellow solid without further purification.

#### 4.1.5. N-(5-Bromo-1H-thieno[3,2-c]pyrazol-3-yl)benzamide (15e)

According to the same procedures for preparing **15a**, compound **15e** was obtained from 5-bromo-1*H*-thieno[3,2-*c*]pyrazol-3-amine (**14**) and benzoyl chloride as a yellow solid without further purification.

#### 4.1.6. N-(5-(pyridin-3-yl)-1H-thieno[3,2-c]pyrazol-3-yl)cyclopropanecarboxamide (16a)

To a solution of **15a** (85.8 mg, 0.3 mmol) and 3-(4,4,5,5-tetramethyl-1,3,2-dioxaborolan-2-yl)pyridine (123 mg, 0.6 mmol) in DMF (2 ml), EtOH (1 ml) and distilled water (1 ml) was added Pd(dppf)Cl_2_ (11 mg, 0.015 mmol) and CH_3_CO_2_K (88 mg, 0.9 mmol). The mixture was stirred at 100 °C for 12 h. Distilled water (5 ml) was added, and the mixture was extracted with EtOAc (20 ml × 3). The organic phase was washed with brine and dried over Na_2_SO_4_. After filtration and evaporation, the residue was purified by silica gel chromatography (CH_2_Cl_2_/MeOH = 40:1) to give **16a** (53.1 mg, 18.7%) as a white solid. Mp: 224 − 226 °C. ^1^H NMR (600 MHz, DMSO-*d*_6_) δ 12.56 (s, 1H), 11.11 (s, 1H), 8.94 (s, 1H), 8.53 (d, *J* = 4.8 Hz, 1H), 8.10 − 8.06 (m, 1H), 7.54 (s, 1H), 7.46 (dd, *J* = 8.0, 4.8 Hz, 1H), 1.97 − 1.84 (m, 1H), 0.94 − 0.74 (m, 4H); ^13 ^C NMR (150 MHz, DMSO-*d*_6_) δ 171.93, 149.43, 148.44, 146.61, 146.46, 140.03, 133.11, 131.16, 124.52, 110.97, 107.23, 13.94, 7.75; HRMS (ESI): calcd. for C_14_H_13_N_4_OS [M + H]^+^: 285.0805, found: 285.0811. Purity: 95.2%.

#### 4.1.7. N-(5-(pyridin-3-yl)-1H-thieno[3,2-c]pyrazol-3-yl)isobutyramide (16b)

According to the same procedures for preparing **16a**, compound **16b** was obtained from **15b** and 3-(4,4,5,5-tetramethyl-1,3,2-dioxaborolan-2-yl)pyridine in 18.0% yield as a white solid. Mp: > 240 °C. ^1^H NMR (600 MHz, DMSO-*d*_6_) δ 12.56 (s, 1H), 10.76 (s, 1H), 8.95 (d, *J* = 1.8 Hz, 1H), 8.54 (d, *J* = 4.8 Hz, 1H), 8.11 − 8.07 (m, 1H), 7.54 (s, 1H), 7.46 (dd, *J* = 8.0, 4.8 Hz, 1H), 2.73 − 2.65 (m, 1H), 1.11 (d, *J* = 6.9 Hz, 6H); ^13 ^C NMR (150 MHz, DMSO-*d*_6_) δ 175.45, 149.44, 148.42, 146.62, 146.45, 140.03, 133.11, 131.17, 124.52, 111.06, 107.23, 34.21, 19.88; HRMS (ESI): calcd. for C_14_H_15_N_4_OS [M + H]^+^: 287.0961, found: 287.0971. Purity: 99.3%.

#### 4.1.8. N-(5-(pyridin-3-yl)-1H-thieno[3,2-c]pyrazol-3-yl)butyramide (16c)

According to the same procedures for preparing **16a**, compound **16c** was obtained from **15c** and 3-(4,4,5,5-tetramethyl-1,3,2-dioxaborolan-2-yl)pyridine in 55.9% yield as a white solid. Mp: 238 − 240 °C. ^1^H NMR (600 MHz, DMSO-*d*_6_) δ 12.55 (s, 1H), 10.77 (s, 1H), 8.95 − 8.93 (m, 1H), 8.52 (dd, *J* = 4.8, 1.2 Hz, 1H), 8.10 − 8.05 (m, 1H), 7.53 (s, 1H), 7.45 (dd, *J* = 8.0, 4.8 Hz, 1H), 2.32 (t, *J* = 7.4 Hz, 2H), 1.61 (m, 2H), 0.91 (t, *J* = 7.4 Hz, 3H); ^13 ^C NMR (150 MHz, DMSO-*d*_6_) δ 171.35, 149.45, 148.42, 146.63, 146.46, 139.92, 133.14, 131.16, 124.53, 111.06, 107.24, 37.44, 18.83, 14.09; HRMS (ESI): calcd. for C_14_H_15_N_4_S [M + H]^+^: 287.0961, found: 287.0975. Purity: 99.9%.

#### 4.1.9. N-(5-(pyridin-3-yl)-1H-thieno[3,2-c]pyrazol-3-yl)propane-1-sulphonamide (16d)

According to the same procedures for preparing **16a**, compound **16d** was obtained from **15d** and 3-(4,4,5,5-tetramethyl-1,3,2-dioxaborolan-2-yl)pyridine in 11.5% yield as a white solid. Mp: 208 − 210 °C. ^1^H NMR (600 MHz, DMSO-*d*_6_) δ 12.83 (s, 1H), 10.43 (s, 1H), 8.97 (d, *J* = 1.8 Hz, 1H), 8.56 (dd, *J* = 4.8, 1.2 Hz, 1H), 8.12 − 8.08 (m, 1H), 7.62 (s, 1H), 7.48 (dd, *J* = 8.0, 4.8 Hz, 1H), 3.20 − 3.12 (m, 2H), 1.81 − 1.70 (m, 2H), 0.96 (t, *J* = 7.5 Hz, 3H); ^13 ^C NMR (150 MHz, DMSO-*d*_6_) δ 149.69, 149.59, 146.78, 146.44, 138.20, 133.37, 130.82, 124.55, 111.39, 107.77, 54.05, 17.29, 13.07; HRMS (ESI): calcd. for C_13_H_15_N_4_O_2_S_2_ [M + H]^+^: 323.0631, found: 323.0634. Purity: 95.8%.

#### 4.1.10. N-(5-(pyridin-3-yl)-1H-thieno[3,2-c]pyrazol-3-yl)benzamide (16e)

According to the same procedures for preparing **16a**, compound **16e** was obtained from **15e** and 3-(4,4,5,5-tetramethyl-1,3,2-dioxaborolan-2-yl)pyridine in 16.3% yield as a white solid. Mp: > 240 °C. ^1^H NMR (600 MHz, DMSO-*d*_6_) δ 12.77 (s, 1H), 11.33 (s, 1H), 9.00 (s, 1H), 8.56 (s, 1H), 8.13 (d, *J* = 7.5 Hz, 1H), 8.09 (d, *J* = 7.4 Hz, 2H), 7.61 (s, 2H), 7.54 (t, *J* = 7.3 Hz, 2H), 7.49 (d, *J* = 5.2 Hz, 1H); ^13 ^C NMR (150 MHz, DMSO-*d*_6_) δ 165.29, 149.50, 148.47, 146.66, 146.52, 140.12, 133.66, 133.21, 132.44, 131.17, 128.89, 128.42, 124.59, 111.74, 107.32; HRMS (ESI): calcd. for C_17_H_13_N_4_OS [M + H]^+^: 321.0805, found: 321.0794. Purity: 98.7%.

#### 4.1.11. N-(5-(5-Phenylpyridin-3-yl)-1H-thieno[3,2-c]pyrazol-3-yl)cyclopropanecarboxamide (17a)

According to the same procedures for preparing **16a**, compound **17a** was obtained from **15a** and 3-phenyl-5-(4,4,5,5-tetramethyl-1,3,2-dioxaborolan-2-yl)pyridine in 13.2% yield as a white solid. Mp: > 240 °C. ^1^H NMR (600 MHz, DMSO-*d*_6_) δ 12.61 (s, 1H), 11.13 (s, 1H), 8.91 (d, *J* = 2.3 Hz, 1H), 8.85 (d, *J* = 2.2 Hz, 1H), 8.30 (t, *J* = 2.2 Hz, 1H), 7.84 (d, *J* = 7.3 Hz, 2H), 7.70 (s, 1H), 7.54 (t, *J* = 7.6 Hz, 2H), 7.47 (t, *J* = 7.6 Hz, 1H), 1.96 − 1.88 (m, 1H), 0.90 − 0.79 (m, 4H); ^13 ^C NMR (150 MHz, DMSO-*d*_6_) δ 171.96, 148.46, 147.51, 146.22, 145.42, 140.06, 137.01, 136.28, 131.30, 130.88, 129.62, 128.91, 127.63, 111.10, 107.84, 13.95, 7.76; HRMS (ESI): calcd. for C_20_H_17_N_4_OS [M + H]^+^: 361.1118, found: 361.1123. Purity: 99.9%.

#### 4.1.12. N-(5-(5-Phenylpyridin-3-yl)-1H-thieno[3,2-c]pyrazol-3-yl)isobutyramide (17b)

According to the same procedures for preparing **16a**, compound **17b** was obtained from **15b** and 3-phenyl-5-(4,4,5,5-tetramethyl-1,3,2-dioxaborolan-2-yl)pyridine in 15.7% yield as a white solid. Mp: > 240 °C. ^1^H NMR (600 MHz, DMSO-*d*_6_) δ 12.61 (s, 1H), 10.79 (s, 1H), 8.92 (d, *J* = 2.1 Hz, 1H), 8.85 (d, *J* = 2.1 Hz, 1H), 8.32 (t, *J* = 2.1 Hz, 1H), 7.88 − 7.83 (m, 2H), 7.70 (s, 1H), 7.54 (t, *J* = 7.6 Hz, 2H), 7.47 (t, *J* = 7.6 Hz, 1H), 2.73 − 2.68 (m, 1H), 1.12 (d, *J* = 6.9 Hz, 6H); ^13 ^C NMR (150 MHz, DMSO-*d*_6_) δ 175.48, 148.44, 147.51, 146.21, 145.43, 140.05, 137.01, 136.29, 131.31, 130.87, 129.62, 128.91, 127.63, 111.20, 107.83, 34.22, 19.88; HRMS (ESI): calcd. for C_20_H_19_N_4_OS [M + H]^+^: 363.1274, found: 363.1265. Purity: 99.8%.

#### 4.1.13. N-(5-(5-Phenylpyridin-3-yl)-1H-thieno[3,2-c]pyrazol-3-yl)butyramide (17c)

According to the same procedures for preparing **16a**, compound **17c** was obtained from **15c** and 3-phenyl-5-(4,4,5,5-tetramethyl-1,3,2-dioxaborolan-2-yl)pyridine in 38.6% yield as a white solid. Mp: > 240 °C. ^1^H NMR (600 MHz, DMSO-*d*_6_) δ 12.60 (s, 1H), 10.79 (s, 1H), 8.91 (d, *J* = 1.9 Hz, 1H), 8.85 (d, *J* = 1.9 Hz, 1H), 8.32 (t, *J* = 1.9 Hz, 1H), 7.87 − 7.83 (m, 2H), 7.70 (s, 1H), 7.56 − 7.52 (m, 2H), 7.47 (t, *J* = 7.4 Hz, 1H), 2.34 (t, *J* = 7.3 Hz, 2H), 1.67 − 1.58 (m, 2H), 0.92 (t, *J* = 7.4 Hz, 3H); ^13 ^C NMR (150 MHz, DMSO-*d*_6_) δ 170.28, 147.38, 146.47, 145.15, 144.39, 138.89, 135.96, 135.24, 130.26, 129.84, 128.56, 127.85, 126.58, 110.13, 106.81, 36.37, 17.76, 13.03; HRMS (ESI): calcd. for C_20_H_18_N_4_OS [M + H]^+^: 363.1274, found: 363.1288. Purity: 99.6%.

#### 4.1.14. N-(5-(5-Phenylpyridin-3-yl)-1H-thieno[3,2-c]pyrazol-3-yl)propane-1-sulphonamide (17d)

According to the same procedures for preparing **16a**, compound **17d** was obtained from **15d** and 3-phenyl-5-(4,4,5,5-tetramethyl-1,3,2-dioxaborolan-2-yl)pyridine in 12.7% yield as a white solid. Mp: 112 − 114 °C. ^1^H NMR (600 MHz, DMSO-*d*_6_) δ 12.88 (s, 1H), 10.45 (s, 1H), 8.91 (s, 1H), 8.86 (s, 1H), 8.33 (s, 1H), 7.84 (d, *J* = 7.5 Hz, 2H), 7.77 (s, 1H), 7.54 (t, *J* = 7.5 Hz, 2H), 7.46 (t, *J* = 7.3 Hz, 1H), 3.21 − 3.03 (m, 2H), 1.83 − 1.63 (m, 2H), 0.96 (t, *J* = 7.4 Hz, 3H); ^13 ^C NMR (150 MHz, DMSO-*d*_6_) δ 149.14, 147.32, 145.72, 145.11, 137.76, 136.49, 135.88, 130.68, 130.52, 129.15, 128.48, 127.21, 111.08, 107.94, 53.60, 16.84, 12.61; HRMS (ESI): calcd. for C_19_H_19_N_4_O_2_S_2_ [M + H]^+^: 399.0944, found: 399.0929. Purity: 96.5%.

#### 4.1.15. N-(5-(4-Phenylpyridin-3-yl)-1H-thieno[3,2-c]pyrazol-3-yl)isobutyramide (18a)

To a solution of **15b** (83 mg, 0.289 mmol) and (4-phenylpyridin-3-yl)boronic acid (115 mg, 0.578 mmol) in DMF (2 ml), 1,4-dioxane (1 ml) and distilled water (0.5 ml) was added Pd(dppf)Cl_2_ (9.94 mg, 0.0136 mmol) and CH_3_CO_2_K (53.3 mg, 0.544 mmol). The mixture was stirred at 100 °C for 15 h. Distilled water (5 ml) was added, and the mixture was extracted with EtOAc (20 ml × 3). The organic phase was washed with brine and dried over Na_2_SO_4_. After filtration and evaporation, the residue was purified by silica gel chromatography (hexane/EtOAc = 1:1) to give **18a** (21 mg, 20.0%) as a yellow solid. Mp: > 240 °C. ^1^H NMR (400 MHz, DMSO-*d*_6_) δ 12.32 (s, 1H), 10.69 (s, 1H), 8.71 (s, 1H), 8.63 (d, *J* = 4.8 Hz, 1H), 7.43 (d, *J* = 4.8 Hz, 1H), 7.41 − 7.32 (m, 5H), 6.68 (s, 1H), 2.71 − 2.59 (m, 1H), 1.06 (d, *J* = 6.8 Hz, 6H); ^13 ^C NMR (100 MHz, DMSO-*d*_6_) δ 174.83, 150.41, 149.42, 147.46, 147.35, 145.28, 139.25, 138.03, 129.17, 128.75, 128.55, 128.35, 124.82, 111.55, 110.09, 33.72, 19.36; HRMS (ESI): calcd. for C_20_H_19_N_4_OS [M + H]^+^: 363.1274, found: 363.1270. Purity: 99.3%.

#### 4.1.16. N-(5-(4-(2-Methylphenyl)pyridin-3-yl)-1H-thieno[3,2-c]pyrazol-3-yl)isobutyramide (18b)

According to the same procedures for preparing **18a**, compound **18b** was obtained from **15b** and (4-(2-methylphenyl)pyridin-3-yl)boronic acid in 20.6% yield as a white solid. Mp: > 240 °C. ^1^H NMR (400 MHz, DMSO-*d*_6_) δ 12.28 (s, 1H), 10.67 (s, 1H), 8.79 (s, 1H), 8.61 (d, *J* = 4.4 Hz, 1H), 7.37 − 7.17 (m, 5H), 6.46 (s, 1H), 2.69 − 2.59 (m, 1H), 1.94 (s, 3H), 1.06 (d, *J* = 6.4 Hz, 6H); ^13 ^C NMR (100 MHz, DMSO-*d*_6_) δ 175.29, 149.88, 149.36, 147.81, 147.59, 145.47, 139.71, 138.49, 135.24, 130.55, 130.24, 129.58, 128.93, 126.54, 125.66, 111.70, 109.76, 34.19, 19.94, 19.83; HRMS (ESI): calcd. for C_21_H_21_N_4_OS [M + H]^+^: 377.1431, found: 377.1426. Purity: 97.6%.

#### 4.1.17. N-(5-(4-(3-Methylphenyl)pyridin-3-yl)-1H-thieno[3,2-c]pyrazol-3-yl)isobutyramide (18c)

According to the same procedures for preparing **18a**, compound **18c** was obtained from **15b** and (4-(3-methylphenyl)pyridin-3-yl)boronic acid in 19.0% yield as a yellow solid. Mp: 236 − 238 °C. ^1^H NMR (400 MHz, DMSO-*d*_6_) δ 12.33 (s, 1H), 10.71 (s, 1H), 8.69 (s, 1H), 8.61 (d, *J* = 5.0 Hz, 1H), 7.41 (d, *J* = 5.0 Hz, 1H), 7.27 − 7.16 (m, 3H), 7.07 (d, *J* = 7.4 Hz, 1H), 6.67 (s, 1H), 2.69 − 2.60 (m, 1H), 2.30 (s, 3H), 1.06 (d, *J* = 6.8 Hz, 6H); ^13 ^C NMR (100 MHz, DMSO-*d*_6_) δ 174.84, 150.36, 149.33, 147.55, 147.36, 145.29, 139.25, 138.04, 137.82, 129.22, 129.13, 128.99, 128.29, 125.91, 124.85, 111.45, 110.04, 33.72, 21.03, 19.37; HRMS (ESI): calcd. for C_21_H_21_N_4_OS [M + H]^+^: 377.1431, found: 377.1441. Purity: 96.6%.

#### 4.1.18. N-(5-(4-(4-Methylphenyl)pyridin-3-yl)-1H-thieno[3,2-c]pyrazol-3-yl)isobutyramide (18d)

According to the same procedures for preparing **18a**, compound **18d** was obtained from **15b** and (4-(4-methylphenyl)pyridin-3-yl)boronic acid in 24.1% yield as a yellow solid. Mp: 239 − 240 °C. ^1^H NMR (400 MHz, DMSO-*d*_6_) δ 12.33 (s, 1H), 10.70 (s, 1H), 8.67 (s, 1H), 8.61 (d, *J* = 5.0 Hz, 1H), 7.40 (d, *J* = 5.0 Hz, 1H), 7.24 (d, *J* = 8.0 Hz, 2H), 7.18 (d, *J* = 8.0 Hz, 2H), 6.70 (s, 1H), 2.70 − 2.58 (m, 1H), 2.30 (s, 3H), 1.06 (d, *J* = 6.8 Hz, 6H); ^13 ^C NMR (100 MHz, DMSO-*d*_6_) δ 174.83, 150.49, 149.41, 147.46, 147.37, 145.46, 139.25, 138.66, 137.75, 135.08, 129.17, 128.65, 124.78, 111.51, 110.02, 33.71, 20.77, 19.35; HRMS (ESI): calcd. for C_21_H_21_N_4_OS [M + H]^+^: 377.1431, found: 377.1435. Purity: 95.7%.

#### 4.1.19. N-(5-(4-(2-Methoxyphenyl)pyridin-3-yl)-1H-thieno[3,2-c]pyrazol-3-yl)isobutyramide (18e)

According to the same procedures for preparing **18a**, compound **18e** was obtained from **15b** and (4-(2-methoxyphenyl)pyridin-3-yl)boronic acid in 12.2% yield as a white solid. Mp: 213 − 215 °C. ^1^H NMR (400 MHz, DMSO-*d*_6_) δ 12.25 (s, 1H), 10.65 (s, 1H), 8.71 (s, 1H), 8.58 (s, 1H), 7.42 − 7.28 (m, 2H), 7.18 (d, *J* = 6.4 Hz, 1H), 7.09 − 6.95 (m, 2H), 6.51 (s, 1H), 3.53 (s, 3H), 2.69 − 2.60 (m, 1H), 1.06 (d, *J* = 9.0 Hz, 6H); ^13 ^C NMR (100 MHz, DMSO-*d*_6_) δ 174.76, 155.97, 149.33, 148.71, 147.33, 145.72, 144.74, 139.20, 130.42, 130.18, 127.06, 125.67, 120.72, 111.62, 111.07, 108.48, 99.52, 55.32, 33.73, 19.38; HRMS (ESI): calcd. for C_21_H_21_N_4_O_2_S [M + H]^+^: 393.1380, found: 393.1381. Purity: 98.1%.

#### 4.1.20. N-(5-(4-(3-Methoxyphenyl)pyridin-3-yl)-1H-thieno[3,2-c]pyrazol-3-yl)isobutyramide (18f)

According to the same procedures for preparing **18a**, compound **18f** was obtained from **15b** and (4-(3-methoxyphenyl)pyridin-3-yl)boronic acid in 20.0% yield as a white solid. Mp: 212 − 214 °C. ^1^H NMR (400 MHz, DMSO-*d*_6_) δ 12.33 (s, 1H), 10.70 (s, 1H), 8.70 (s, 1H), 8.63 (d, *J* = 5.0 Hz, 1H), 7.45 (d, *J* = 5.0 Hz, 1H), 7.28 (t, *J* = 8.2 Hz, 1H), 6.97 − 6.85 (m, 3H), 6.72 (s, 1H), 3.70 (s, 3H), 2.70 − 2.61 (m, 1H), 1.06 (d, *J* = 6.8 Hz, 6H); ^13 ^C NMR (100 MHz, DMSO-*d*_6_) δ 174.84, 159.11, 150.39, 149.39, 147.38, 147.32, 145.25, 139.36, 139.25, 129.64, 129.15, 124.74, 121.01, 114.44, 113.80, 111.51, 110.10, 55.05, 33.72, 19.36; HRMS (ESI): calcd. for C_21_H_21_N_4_O_2_S [M + H]^+^: 393.1380, found: 393.1381. Purity: 99.7%.

#### 4.1.21. N-(5-(4-(4-Methoxyphenyl)pyridin-3-yl)-1H-thieno[3,2-c]pyrazol-3-yl)isobutyramide (18g)

According to the same procedures for preparing **18a**, compound **18g** was obtained from **15b** and (4-(4-methoxyphenyl)pyridin-3-yl)boronic acid in 14.7% yield as a yellow solid. Mp: >240 °C. ^1^H NMR (400 MHz, DMSO-*d*_6_) δ 12.35 (s, 1H), 10.71 (s, 1H), 8.65 (s, 1H), 8.59 (d, *J* = 4.8 Hz, 1H), 7.41 (d, *J* = 4.8 Hz, 1H), 7.28 (d, *J* = 8.2 Hz, 2H), 6.94 (d, *J* = 8.2 Hz, 2H), 6.73 (s, 1H), 3.76 (s, 3H), 2.69 − 2.60 (m, 1H), 1.06 (d, *J* = 6.4 Hz, 6H); ^13 ^C NMR (100 MHz, DMSO-*d*_6_) δ 174.83, 159.32, 150.53, 149.39, 147.40, 147.12, 145.67, 139.28, 139.26, 130.13, 130.02, 129.06, 124.71, 114.05, 109.91, 55.13, 33.72, 19.36; HRMS (ESI): calcd. for C_21_H_21_N_4_O_2_S [M + H]^+^: 393.1380, found: 393.1379. Purity: 99.8%.

#### 4.1.22. N-(5-(4-(2-Fluorophenyl)pyridin-3-yl)-1H-thieno[3,2-c]pyrazol-3-yl)isobutyramide (18h)

According to the same procedures for preparing **18a**, compound **18h** was obtained from **15b** and (4-(2-fluorophenyl)pyridin-3-yl)boronic acid in 25.7% yield as a yellow solid. Mp: > 240 °C. ^1^H NMR (400 MHz, DMSO-*d*_6_) δ 12.53 (s, 1H), 10.77 (s, 1H), 8.70 (d, *J* = 4.8 Hz, 2H), 7.88 (t, *J* = 7.2 Hz, 1H), 7.65 (d, *J* = 4.8 Hz, 2H), 7.58 (t, *J* = 7.2 Hz, 1H), 7.46 − 7.40 (m, 2H), 2.72 − 2.65 (m, 1H), 1.11 (d, *J* = 6.8 Hz, 6H); ^13 ^C NMR (100 MHz, DMSO-*d*_6_) δ 174.95, 155.42 (d, *J_C-F_* = 253.1 Hz), 149.89, 147.69, 142.54, 142.24, 142.21, 139.39, 130.19, 130.16, 129.51 (d, *J_C-F_* = 3.2 Hz), 126.86 (d, *J_C-F_* = 14.0 Hz), 125.50 (d, *J_C-F_* = 3.9 Hz), 123.76 (d, *J_C-F_* = 1.8 Hz), 123.22 (d, *J_C-F_* = 13.4 Hz), 111.13, 109.01, 33.74, 19.38; HRMS (ESI): calcd. for C_20_H_18_FN_4_OS [M + H]^+^: 381.1180, found: 381.1194. Purity: 99.7%.

#### 4.1.23. N-(5-(4-(3-Fluorophenyl)pyridin-3-yl)-1H-thieno[3,2-c]pyrazol-3-yl)isobutyramide (18i)

According to the same procedures for preparing **18a**, compound **18i** was obtained from **15b** and (4-(3-fluorophenyl)pyridin-3-yl)boronic acid in 20.5% yield as a yellow solid. Mp: > 240 °C. ^1^H NMR (400 MHz, DMSO-*d*_6_) δ 12.54 (s, 1H), 10.78 (s, 1H), 8.68 (d, *J* = 4.7 Hz, 2H), 7.95 (t, *J* = 8.2 Hz, 1H), 7.92 − 7.74 (m, 4H), 7.50 (s, 1H), 2.75 − 2.66 (m, 1H), 1.12 (d, *J* = 6.8 Hz, 6H); ^13 ^C NMR (100 MHz, DMSO-*d*_6_) δ 174.97, 158.90 (d, *J_C-F_* = 249.3 Hz), 150.35, 147.72, 144.77, 144.75, 142.00, 141.96, 139.41, 138.36, 138.28, 129.10 (d, *J_C-F_* = 3.8 Hz), 123.30 (d, *J_C-F_* = 2.7 Hz), 122.94 (d, *J_C-F_* = 12.7 Hz), 120.99, 114.73 (d, *J_C-F_* = 24.1 Hz), 108.76, 33.75, 19.38; HRMS (ESI): calcd. for C_20_H_18_FN_4_OS [M + H]^+^: 381.1180, found: 381.1192. Purity: 99.0%.

#### 4.1.24. N-(5-(4-(4-Fluorophenyl)pyridin-3-yl)-1H-thieno[3,2-c]pyrazol-3-yl)isobutyramide (18j)

According to the same procedures for preparing **18a**, compound **18i** was obtained from **15b** and (4-(4-fluorophenyl)pyridin-3-yl)boronic acid in 32.6% yield as a yellow solid. Mp: > 240 °C. ^1^H NMR (400 MHz, DMSO-*d*_6_) δ 12.57 (s, 1H), 10.78 (s, 1H), 8.66 (d, *J* = 5.6 Hz, 2H), 8.15 (dd, *J* = 7.2, 2.2 Hz, 1H), 7.85 − 7.78 (m, 3H), 7.58 (s, 1H), 7.55 − 7.47 (m, 1H), 2.74 − 2.65 (m, 1H), 1.11 (d, *J* = 6.8 Hz, 6H); ^13 ^C NMR (100 MHz, DMSO-*d*_6_) δ 174.98, 159.05 (d, *J_C-F_* = 251.9 Hz), 150.24, 147.63, 145.57, 142.05, 139.42, 134.33, 128.26 (d, *J_C-F_* = 9.1 Hz), 127.01, 123.11, 122.98, 121.39, 117.42 (d, *J_C-F_* = 23.0 Hz), 109.00, 33.75, 19.40; HRMS (ESI): calcd. for C_20_H_18_FN_4_OS [M + H]^+^: 381.1180, found: 381.1190. Purity: 98.1%.

#### 4.1.25. N-(5-(4-(3-(trifluoromethyl)phenyl)pyridin-3-yl)-1H-thieno[3,2-c]pyrazol-3-yl)isobutyramide (18k)

According to the same procedures for preparing **18a**, compound **18k** was obtained from **15b** and (4-(3-(trifluoromethyl)phenyl)pyridin-3-yl)boronic acid in 19.4% yield as a yellow solid. Mp: 202 − 204 °C. ^1^H NMR (400 MHz, DMSO-*d*_6_) δ 12.33 (s, 1H), 10.70 (s, 1H), 8.75 (s, 1H), 8.68 (d, *J* = 3.0 Hz, 1H), 7.73 (s, 2H), 7.62 (s, 2H), 7.54 (d, *J* = 3.0 Hz, 1H), 6.69 (s, 1H), 2.68 − 2.58 (m, 1H), 1.06 (d, *J* = 5.6 Hz, 6H); ^13 ^C NMR (100 MHz, DMSO-*d*_6_) δ 174.81, 150.45, 149.56, 147.28, 145.78, 144.47, 139.20, 138.93, 132.95, 129.53, 129.26 (q, *J_C-F_* = 32.1 Hz), 129.11, 125.39 (q, *J_C-F_* = 3.7 Hz), 125.01 (q, *J_C-F_* = 3.7 Hz), 124.72, 123.90 (q, *J_C-F_* = 271 Hz), 111.55, 110.48, 33.68, 19.28; HRMS (ESI): calcd. for C_21_H_18_F_3_N_4_OS [M + H]^+^: 431.1148, found: 431.1154. Purity: 98.6%.

#### 4.1.26. N-(5-(4-(4-(trifluoromethyl)phenyl)pyridin-3-yl)-1H-thieno[3,2-c]pyrazol-3-yl)isobutyramide (18l)

According to the same procedures for preparing **18a**, compound **18l** was obtained from **15b** and (4-(4-(trifluoromethyl)phenyl)pyridin-3-yl)boronic acid in 21.1% yield as a yellow solid. Mp: >240 °C. ^1^H NMR (400 MHz, DMSO-*d*_6_) δ 12.33 (s, 1H), 10.69 (s, 1H), 8.76 (s, 1H), 8.69 (d, *J* = 4.8 Hz, 1H), 7.76 (d, *J* = 7.9 Hz, 2H), 7.58 (d, *J* = 7.9 Hz, 2H), 7.49 (d, *J* = 4.8 Hz, 1H), 6.72 (s, 1H), 2.68 − 2.59 (m, 1H), 1.06 (d, *J* = 6.7 Hz, 6H); ^13 ^C NMR (100 MHz, DMSO-*d*_6_) δ 174.84, 150.47, 149.51, 147.32, 145.95, 144.54, 142.19, 139.24, 129.70, 129.06, 128.65 (q, *J_C-F_* = 31.8 Hz), 125.37 (q, *J_C-F_* = 3.7 Hz), 124.62, 124.03 (q, *J_C-F_* = 271 Hz), 111.70, 110.40, 33.67, 19.27; HRMS (ESI): calcd. for C_21_H_18_F_3_N_4_OS [M + H]^+^: 431.1148, found: 431.1156. Purity: 98.9%.

#### 4.1.27. N-(5-(4-([1,1'-Biphenyl]-4-yl)pyridin-3-yl)-1H-thieno[3,2-c]pyrazol-3-yl)isobutyramide (18m)

According to the same procedures for preparing **18a**, compound **18m** was obtained from **15b** and (4-([1,1′-biphenyl]-4-yl)pyridin-3-yl)boronic acid in 13.5% yield as a yellow solid. Mp: 159 − 161 °C. ^1^H NMR (400 MHz, DMSO-*d*_6_) δ 12.36 (s, 1H), 10.70 (s, 1H), 8.72 (s, 1H), 8.65 (d, *J* = 5.0 Hz, 1H), 7.74 − 7.68 (m, 4H), 7.50 − 7.47 (m, 2H), 7.46 − 7.43 (m, 3H), 7.38 (d, *J* = 7.4 Hz, 1H), 6.76 (s, 1H), 2.69 − 2.59 (m, 1H), 1.05 (d, *J* = 6.8 Hz, 6H); ^13 ^C NMR (100 MHz, DMSO-*d*_6_) δ 174.83, 150.52, 149.45, 147.39, 146.99, 145.25, 139.81, 139.27, 139.11, 137.03, 129.40, 129.10, 128.96, 127.74, 126.67, 126.59, 124.74, 111.58, 110.15, 33.68, 19.32; HRMS (ESI): calcd. for C_26_H_23_N_4_OS [M + H]^+^: 439.1587, found: 439.1588. Purity: 95.0%.

#### 4.1.28. N-(5-(4-(naphthalen-2-yl)pyridin-3-yl)-1H-thieno[3,2-c]pyrazol-3-yl)isobutyramide (18n)

According to the same procedures for preparing **18a**, compound **18n** was obtained from **15b** and (4-(naphthalen-2-yl)pyridin-3-yl)boronic acid in 24.2% yield as a yellow solid. Mp: > 240 °C. ^1^H NMR (400 MHz, DMSO-*d*_6_) δ 12.30 (s, 1H), 10.68 (s, 1H), 8.76 (s, 1H), 8.68 (d, *J* = 5.0 Hz, 1H), 8.03 (s, 1H), 7.97 − 7.90 (m, 2H), 7.86 (d, *J* = 8.5 Hz, 1H), 7.59 − 7.54 (m, 3H), 7.37 (dd, *J* = 8.5, 1.4 Hz, 1H), 6.68 (s, 1H), 2.66 − 2.59 (m, 1H), 1.04 (d, *J* = 6.8 Hz, 6H); ^13 ^C NMR (100 MHz, DMSO-*d*_6_) δ 174.80, 150.39, 149.41, 147.37, 147.32, 145.11, 139.19, 135.75, 132.85, 132.38, 129.29, 128.21, 127.89, 127.73, 127.57, 126.76, 126.52, 126.49, 125.13, 111.45, 110.29, 33.66, 19.30; HRMS (ESI): calcd. for C_24_H_21_N_4_OS [M + H]^+^: 413.1431, found: 413.1431. Purity: 98.3%.

### 4.2. GSK-3β kinase assay

The GSK-3β inhibition assay was performed by calliper mobility shift assay using the method described previously^64^^,^^75^ and AR-A014418 was used as a positive control. In brief, compounds or AR-A014418 were tested from 1 μM or 5 μM, 3-fold dilution for IC_50_ determination. GSK-3β protein and the tested compound were loaded in 384-well plate (Corning). After incubation for 10 min, the FAM-labeled peptide 15 (GL Biochem, Shanghai, China) and ATP prepared in the reaction buffer were added and ran for 1 h at 28 °C. Stop buffer (25 μL) was added and conversion data were collected on a LabChip EZ Reader (PerkinElmer, Shanghai, China) at each concentration through the direct detection of both substrate and product via Laser-Induced Fluorescence (LIF) at 492 nm. The IC_50_ values were calculated from dose-response curves using XLfit (curve fitting software for Excel).

### 4.3. Kinase selectivity screen

Compound **16b** was evaluated for kinase selectivity at Eurofins Cerep SA (Celle-L’Evescault, France) in enzymatic radioactive assays in a panel of 21 different kinases (including GSK-3β) from diverse families. Protein kinase were assayed in the presence of 1.0 μM compound **16b** or vehicle (DMSO). The enzymatic activity was measured in the presence of *K*_m_ ATP.

### 4.4. Molecular docking

Molecular docking was performed on the Sybyl-X 2.0 software (Tripos, St. Louis, MO) and the X-ray crystal structure of GSK-3β (PDB: 4ACG) was obtained from the RCSB Protein Data Bank. The protein was added with hydrogen atoms and charges. Waters were removed from the PDB file. Native ligand (6LQ) was extracted from the protein and used as a standard to generate the protomol. The binding pocket was defined as all residues within 5 Å of the original ligand. Finally, docking was performed by using the Surflex-Dock mode, and the conformations were used to analyse the interactions between ligand and GSK-3β. UCSF Chimaera 1.16 was used to visualise the result of docking ^76^. Maestro 11.9 was used to show the 2D interactions diagram ^77^.

### 4.5. Cytotoxicity on SH-SY5Y cell line

SH-SY5Y cells were cultured in DMEM/F12 (Dulbecco’s modified Eagle medium and Ham’s F-12, 1:1) with 10% FBS (fetal bovine serum), 1% penicillin and 1% streptomycin under 5% CO_2_ atmosphere at 37 °C. SH-SY5Y cells (1 × 10^5^) were seeded in 96-well plates and incubated for 24 h. Different concentrations of compound **16b** were added into each well and incubated for another 24 h. The survival of cells was determined by MTT assay and the absorbance of each well were measured using a SpectraMax M5 multimode plate reader at 570 nm. Results were expressed as percentage of control and statistical analysis was performed using GraphPad Prism 5.0 (GraphPad Software Inc., San Diego, CA, USA).

### 4.6. Western blot analysis on p-GSK-3β and β-catenin

SH-SY5Y cells (1 × 10^6^) were seeded in 12-well plates (Corning, Los Altos, MA, USA) and incubated with compound **16b** or LiCl at the indicated concentrations for 2.5 h at 37 °C in 5% CO_2_. At the end of incubation, cells were lysed by addition of ice-cold RIPA buffer containing a protease inhibitor cocktail. The protein quantification was determined using a BCA protein assay kit (Jiangsu KeyGEN BioTECH Co., Ltd., Nanjing, China). Cellular lysates were mixed with an equal volume of SDS (sodium dodecyl sulfate) loading buffer (Jiangsu KeyGEN BioTECH Co., Ltd., Nanjing, China) and separated by electrophoresis (Bio-rad Power Supplies Basic, Shanghai, China) in polyacrylamide gel. Proteins were transferred from acrylamide gels to nitrocellulose membranes and blocked in a blocking buffer (PBS, 5% non-fat milk) for 1.5 to 2 h at 20 °C. After overnight incubation at 4 °C with primary p-GSK3β-Ser9 (Cell Signalling Technology, Danvers, MA, USA), or β-catenin (Cell Signalling Technology, Danvers, MA, USA), and GAPDH (Santa Cruz Biotechnology, Shanghai, China), the blots were washed in Tween 20-TBS (TBST, Jiangsu KeyGEN BioTECH Co., Ltd., Nanjing, China) for 20 min and then incubated with secondary antibody (IgG-HRP; Jiangsu KeyGEN BioTECH Co., Ltd., Nanjing, China) for 1 h at room temperature. The blots were washed by TBST for 20 min and detected with ECL chemiluminescent reagent (Jiangsu KeyGEN BioTECH Co., Ltd., Nanjing, China) for 3 min. Pixel intensity was quantitated using gel imaging system (SYNGENE G:BOX/iChemi XR5, ISS, San Diego, CA, US) and Gel-Pro32 software (Media Cybernetics, Bethesda, MD, USA). GAPDH was used as an internal control.

### 4.7. Inhibition of Aβ-induced tau hyperphosphorylation

SH-SY5Y cells were seeded in 12-well plates until 80% confluence, serum-deprived for 12 h. Cells were pre-incubated with compound **16b** or LiCl for 1 h, thereafter stimulated with Aβ_25–35_ (Sigma) for another 6 h. According to the previously reported method^64^^,^^75^, the phosphorylated tau was determined.

### 4.8. Neuronal neurite outgrowth assay and quantitative RT-PCR

SH-SY5Y cells (5 × 10^3^) were planted in 96-well plates and cultivated at 37 °C for 24 h. Compound (RA or **16b,** 10 μM) was then added and cultivated for 72 h. The morphology of neurite outgrowth was examined under an inverted microscope (2 × 100; Olympus, Tokyo, Japan). After the SH-SY5Y cells were cultivated for 24 h, total RNA was extracted, and quantitative RT-PCR was performed according to the previously reported method^75^.

## Supplementary Material

Supplemental Material

## References

[1] Patterson C, World Alzheimer report 2018. London: Alzheimer’s Disease International; 2018.

[2] Srivastava S, Ahmad R, Khare SK. Alzheimer's disease and its treatment by different approaches: A review. Eur J Med Chem 2021;216:1724. 10.1016/j.ejmech.2021.113320 33652356

[3] Liu W, Liu X, Liu W, et al. Discovery of novel β-carboline derivatives as selective AChE inhibitors with GSK-3β inhibitory property for the treatment of Alzheimer's disease. Eur J Med Chem 2022; 229:114095. 34995924 10.1016/j.ejmech.2021.114095

[4] Verma A, Kumar Waiker D, Bhardwaj B, et al. The molecular mechanism, targets, and novel molecules in the treatment of Alzheimer's disease. Bioorg Chem 2022;119:105562. 34952243 10.1016/j.bioorg.2021.105562

[5] Syed YY. Sodium Oligomannate: First approval. Drugs 2020;80:441–4. 32020555 10.1007/s40265-020-01268-1

[6] Dhillon S. Aducanumab: First approval. Drugs 2021;81:1437–43. 34324167 10.1007/s40265-021-01569-z

[7] Wang K, Na L, Duan M. The pathogenesis mechanism, structure properties, potential drugs and therapeutic nanoparticles against the small oligomers of Amyloid-β. Curr Top Med Chem 2021;21:151–67. 32938351 10.2174/1568026620666200916123000

[8] Chen G-F, Xu T-H, Yan Y, et al. Amyloid beta: Structure, biology and structure-based therapeutic development. Acta Pharmacol Sin 2017;38:1205–35. 28713158 10.1038/aps.2017.28 PMC5589967

[9] Anu Kunnath R, Subham D, Alex J, et al. Neurodegenerative pathways in Alzheimer's Disease: A Review. Curr Neuropharmacol 2021;19:679–92. 32851951 10.2174/1570159X18666200807130637 PMC8573750

[10] Tan CC, Zhang XY, Tan L, et al. Tauopathies: Mechanisms and therapeutic strategies. J Alzheimers Dis 2018;61:487–508. 29278892 10.3233/JAD-170187

[11] Congdon EE, Sigurdsson EM. Tau-targeting therapies for Alzheimer disease. Nat Rev Neurol 2018;14:399–415. 29895964 10.1038/s41582-018-0013-z PMC6463489

[12] Li Y, Jiao Q, Xu H, et al. Biometal dyshomeostasis and toxic metal accumulations in the development of Alzheimer’s disease. Front Mol Neurosci 2017;10:339. 29114205 10.3389/fnmol.2017.00339 PMC5660707

[13] Huang WJ, Zhang X, Chen WW. Role of oxidative stress in Alzheimer's disease. Biomed Rep 2016;4:519–22. 27123241 10.3892/br.2016.630 PMC4840676

[14] Wang T, Xu SF, Fan YG, et al. Iron pathophysiology in Alzheimer's Diseases. Adv Exp Med Biol 2019;1173:67–104. 31456206 10.1007/978-981-13-9589-5_5

[15] Kinney JW, Bemiller SM, Murtishaw AS, et al. Inflammation as a central mechanism in Alzheimer's disease. Alzheimers Dement (N Y) 2018;4:575–90. 30406177 10.1016/j.trci.2018.06.014 PMC6214864

[16] Tong BC, Wu AJ, Li M, et al. Calcium signaling in Alzheimer's disease & therapies. Biochim Biophys Acta Mol Cell Res 2018;1865:1745–60. 30059692 10.1016/j.bbamcr.2018.07.018

[17] Gerakis Y, Hetz C. Emerging roles of ER stress in the etiology and pathogenesis of Alzheimer's disease. Febs J 2018;285:995–1011. 29148236 10.1111/febs.14332

[18] Hill E, Wall MJ, Moffat KG, et al. Understanding the pathophysiological actions of tau oligomers: A critical review of current electrophysiological approaches. Front Mol Neurosci 2020;13:155. 32973448 10.3389/fnmol.2020.00155 PMC7468384

[19] Tripathi T, Kalita P. Synergistic effect of Amyloid-β and tau disrupts neural circuits. ACS Chem Neurosci 2019;10:1129–30. 30702852 10.1021/acschemneuro.9b00037

[20] Yin X, Qiu Y, Zhao C, et al. The role of amyloid-beta and tau in the early pathogenesis of Alzheimer's Disease. Med Sci Monit 2021;27:e933084. 34471085 10.12659/MSM.933084 PMC8422899

[21] He Z, Guo JL, McBride JD, et al. Amyloid-β plaques enhance Alzheimer's brain tau-seeded pathologies by facilitating neuritic plaque tau aggregation. Nat Med 2018;24:29–38. 29200205 10.1038/nm.4443 PMC5760353

[22] Bennett RE, DeVos SL, Dujardin S, et al. Enhanced tau aggregation in the presence of amyloid β. Am J Pathol 2017;187:1601–12. 28500862 10.1016/j.ajpath.2017.03.011 PMC5500829

[23] Doble BW, Woodgett JR. Role of glycogen synthase kinase-3 in cell fate and epithelial-mesenchymal transitions. Cells Tissues Organs 2007;185:73–84. 17587811 10.1159/000101306

[24] La Pietra V, La Regina G, Coluccia A, et al. Design, synthesis, and biological evaluation of 1-phenylpyrazolo[3,4-*e*]pyrrolo[3,4-*g*]indolizine-4,6(1*H*,5*H*)-diones as new glycogen synthase kinase-3β inhibitors. J Med Chem 2013;56:10066–78. 24295046 10.1021/jm401466v

[25] Pei JJ, Braak E, Braak H, et al. Distribution of active glycogen synthase kinase 3beta (GSK-3beta) in brains staged for Alzheimer disease neurofibrillary changes. J Neuropathol Exp Neurol 1999;58:1010–9. 10499443 10.1097/00005072-199909000-00011

[26] Georgievska B, Sandin J, Doherty J, et al. AZD1080, a novel GSK3 inhibitor, rescues synaptic plasticity deficits in rodent brain and exhibits peripheral target engagement in humans. J Neurochem 2013;125:446–56. 23410232 10.1111/jnc.12203

[27] Iqbal K, Grundke-Iqbal I. Discoveries of tau, abnormally hyperphosphorylated tau and others of neurofibrillary degeneration: A personal historical perspective. J Alzheimers Dis 2006;9:219–42. 10.3233/jad-2006-9s325 16914861

[28] Wischik CM, Harrington CR, Storey JM. Tau-aggregation inhibitor therapy for Alzheimer's disease. Biochem Pharmacol 2014;88:529–39. 24361915 10.1016/j.bcp.2013.12.008

[29] De Simone A, Tumiatti V, Andrisano V, et al. Glycogen synthase kinase 3β: A new gold rush in Anti-Alzheimer's Disease Multitarget Drug Discovery? J Med Chem 2021;64:26–41. 33346659 10.1021/acs.jmedchem.0c00931 PMC8016207

[30] Llorens-Martin M, Jurado J, Avila J, et al. GSK-3β, a pivotal kinase in Alzheimer disease. Front Mol Neurosci 2014;7:46. 24904272 10.3389/fnmol.2014.00046 PMC4033045

[31] Forlenza OV, De-Paula VJR, Diniz BSO. Neuroprotective effects of lithium: Implications for the treatment of Alzheimer's disease and related neurodegenerative disorders. ACS Chem Neurosci 2014;5:443–50. 24766396 10.1021/cn5000309 PMC4063497

[32] Wang H, Brown J, Martin M. Glycogen synthase kinase 3: A point of convergence for the host inflammatory response. Cytokine 2011;53:130–40. 21095632 10.1016/j.cyto.2010.10.009 PMC3021641

[33] Gómez-Sintes R, Hernández F, Lucas JJ, Avila J. er al. GSK-3 mouse models to study neuronal apoptosis and neurodegeneration. Front Mol Neurosci 2011;4:45. 22110426 10.3389/fnmol.2011.00045 PMC3217194

[34] Sayas CL, Ávila J. GSK-3 and tau: A key duet in Alzheimer’s disease. Cells 2021;10:721. 33804962 10.3390/cells10040721 PMC8063930

[35] Ruiz A, Eldar-Finkelman SM. H. Glycogen synthase kinase-3 inhibitors: Preclinical and clinical focus on CNS-a decade onward. Front Mol Neurosci 2021;14:792364. 35126052 10.3389/fnmol.2021.792364 PMC8813766

[36] del T. S. Phase IIa clinical trial on Alzheimer’s disease with NP12, a GSK3 inhibitor. Alzheimers Dement 2010;6:S147.

[37] Bhat R, Xue Y, Berg S, et al. Structural insights and biological effects of glycogen synthase kinase 3-specific inhibitor AR-A014418. J Biol Chem 2003;278:45937–45. 12928438 10.1074/jbc.M306268200

[38] Berg S, Bergh M, Hellberg S, et al. Discovery of novel potent and highly selective glycogen synthase kinase-3β (GSK3β) inhibitors for Alzheimer's disease: design, synthesis, and characterization of pyrazines. J Med Chem 2012;55:9107–19. 22489897 10.1021/jm201724m

[39] Yao H, Uras G, Zhang P, et al. Discovery of novel tacrine-pyrimidone hybrids as potent dual AChE/GSK-3 inhibitors for the treatment of Alzheimer's Disease. J Med Chem 2021;64:7483–506. 34024109 10.1021/acs.jmedchem.1c00160

[40] Sivaprakasam P, Han X, Civiello RL, et al. Discovery of new acylaminopyridines as GSK-3 inhibitors by a structure guided in-depth exploration of chemical space around a pyrrolopyridinone core. Bioorg Med Chem Lett 2015;25:1856–63. 25845281 10.1016/j.bmcl.2015.03.046

[41] Choi S, Park K, Seo HJ, et al. Preparation of pyrazole derivatives as TNIK, IKKε and TBK1 inhibitor and pharmaceutical composition comprising same. US20160289196. 2016.

[42] Ramurthy S, Pfister KB, Boyce RS, et al. Discovery and optimization of novel pyridines as highly potent and selective glycogen synthase kinase 3 inhibitors. Bioorg Med Chem Lett 2020;30:126930. 31926786 10.1016/j.bmcl.2019.126930

[43] Liu SL, Wang C, Jiang T, et al. The role of CDK5 in Alzheimer's Disease. Mol Neurobiol 2016;53:4328–42. 26227906 10.1007/s12035-015-9369-x

[44] Hanger DP, Anderton BH, Noble W. Tau phosphorylation: The therapeutic challenge for neurodegenerative disease. Trends Mol Med 2009;15:112–9. 19246243 10.1016/j.molmed.2009.01.003

[45] Kimura T, Ishiguro K, Hisanaga S. Physiological and pathological phosphorylation of tau by CDK5. Front Mol Neurosci 2014;7:65. 25076872 10.3389/fnmol.2014.00065 PMC4097945

[46] Perez DI, Gil C, Martinez A. Protein kinases CK1 and CK2 as new targets for neurodegenerative diseases. Med Res Rev 2011;31:924–54. 20577972 10.1002/med.20207

[47] Agholme L, Lindström T, Kågedal K, et al. An *in vitro* model for neuroscience: Differentiation of SH-SY5Y cells into cells with morphological and biochemical characteristics of mature neurons. J Alzheimers Dis 2010;20:1069–82. 20413890 10.3233/JAD-2010-091363

[48] Augello G, Emma MR, Cusimano A, et al. The role of GSK-3 in cancer immunotherapy: GSK-3 inhibitors as a new frontier in cancer treatment. Cells 2020;9:1427. 10.3390/cells9061427 PMC7348946 32526891

[49] Beurel E, Grieco SF, Jope RS. Glycogen synthase kinase-3 (GSK3): Regulation, actions, and diseases. Pharmacol Ther 2015;148:114–31. 25435019 10.1016/j.pharmthera.2014.11.016 PMC4340754

[50] Chalecka-Franaszek E, Chuang DM. Lithium activates the serine/threonine kinase Akt-1 and suppresses glutamate-induced inhibition of Akt-1 activity in neurons. Proc Natl Acad Sci U S A 1999;96:8745–50. 10411946 10.1073/pnas.96.15.8745 PMC17587

[51] Buttrick GJ, Wakefield JG. PI3-K and GSK-3: Akt-ing together with microtubules. Cell Cycle 2008;7:2621–5. 18728390 10.4161/cc.7.17.6514

[52] Ikeda S, Kishida S, Yamamoto H, et al. Axin, a negative regulator of the Wnt signaling pathway, forms a complex with GSK-3beta and beta-catenin and promotes GSK-3beta-dependent phosphorylation of beta-catenin . Embo J 1998;17:1371–84. 9482734 10.1093/emboj/17.5.1371 PMC1170485

[53] Wu D, Pan W. GSK3: A multifaceted kinase in Wnt signaling. Trends Biochem Sci 2010;35:161–8. 19884009 10.1016/j.tibs.2009.10.002 PMC2834833

[54] Stamos JL, Weis WI. The β-catenin destruction complex. Cold Spring Harb Perspect Biol 2013;5:a007898. 23169527 10.1101/cshperspect.a007898 PMC3579403

[55] MacDonald BT, Tamai K, He X. Wnt/beta-catenin signaling: components, mechanisms, and diseases . Dev Cell 2009;17:9–26. 19619488 10.1016/j.devcel.2009.06.016 PMC2861485

[56] Salcedo-Tello P, Ortiz-Matamoros A, Arias C. GSK3 function in the brain during development, neuronal plasticity, and neurodegeneration. Int J Alzheimers Dis 2011;2011:189728. 21660241 10.4061/2011/189728 PMC3109514

[57] Skardelly M, Gaber K, Schwarz J, et al. Neuroprotective effects of the beta-catenin stabilization in an oxygen- and glucose-deprived human neural progenitor cell culture system. Int J Dev Neurosci 2011;29:543–7. 21497193 10.1016/j.ijdevneu.2011.03.010

[58] Jin N, Zhu H, Liang X, et al. Sodium selenate activated Wnt/β-catenin signaling and repressed amyloid-β formation in a triple transgenic mouse model of Alzheimer's disease. Exp Neurol 2017;297:36–49. 28711506 10.1016/j.expneurol.2017.07.006

[59] Toledo EM, Inestrosa NC. Activation of Wnt signaling by lithium and rosiglitazone reduced spatial memory impairment and neurodegeneration in brains of an APPswe/PSEN1DeltaE9 mouse model of Alzheimer's disease. Mol Psychiatry 2010;15:272–85, 19621015 10.1038/mp.2009.72

[60] Lu W, Yamamoto V, Ortega B, et al. Mammalian Ryk is a Wnt coreceptor required for stimulation of neurite outgrowth. Cell 2004;119:97–108. 15454084 10.1016/j.cell.2004.09.019

[61] Lin CC, Chou CH, Howng SL, et al. GSKIP, an inhibitor of GSK3beta, mediates the N-cadherin/beta-catenin pool in the differentiation of SH-SY5Y cells. J Cell Biochem 2009;108:1325–36. 19830702 10.1002/jcb.22362

[62] Yap AS, Brieher WM, Pruschy M, et al. Lateral clustering of the adhesive ectodomain: A fundamental determinant of cadherin function. Curr Biol 1997;7:308–15. 9133345 10.1016/s0960-9822(06)00154-0

[63] González JF, Alcántara AR, Doadrio AL, et al. Developments with multi-target drugs for Alzheimer's disease: an overview of the current discovery approaches. Expert Opin Drug Discov 2019;14:879–91. 31165654 10.1080/17460441.2019.1623201

[64] Shi X-L, Wu J-D, Liu P, et al. Synthesis and evaluation of novel GSK-3β inhibitors as multifunctional agents against Alzheimer's disease. Eur J Med Chem 2019;167:211–25. 30772605 10.1016/j.ejmech.2019.02.001

[65] Moradi HR, Hajali V, Khaksar Z, et al. The next step of neurogenesis in the context of Alzheimer's disease. Mol Biol Rep 2021;48:5647–60. 34232464 10.1007/s11033-021-06520-9

[66] Jaworski T, Banach-Kasper E, Gralec K. GSK-3β at the intersection of neuronal plasticity and neurodegeneration. Neural Plast 2019;2019:4209475. 31191636 10.1155/2019/4209475 PMC6525914

[67] Giese KP. GSK-3: A key player in neurodegeneration and memory. IUBMB Life 2009;61:516–21. 19391164 10.1002/iub.187

[68] Benowitz LI, Routtenberg A. GAP-43: An intrinsic determinant of neuronal development and plasticity. Trends Neurosci 1997;20:84–91. 9023877 10.1016/s0166-2236(96)10072-2

[69] Kawasaki A, Okada M, Tamada A, et al. Growth cone phosphoproteomics reveals that GAP-43 phosphorylated by JNK is a marker of axon growth and regeneration. iScience 2018;4:190–203. 30240740 10.1016/j.isci.2018.05.019 PMC6147025

[70] Knoepfler PS, Cheng PF, Eisenman RN. N-myc is essential during neurogenesis for the rapid expansion of progenitor cell populations and the inhibition of neuronal differentiation. Genes Dev 2002;16:2699–712. 12381668 10.1101/gad.1021202 PMC187459

[71] Nothias F, Vernier P, von Boxberg Y, et al. Modulation of NCAM polysialylation is associated with morphofunctional modifications in the hypothalamo-neurohypophysial system during lactation. Eur J Neurosci 1997;9:1553–65. 9283810 10.1111/j.1460-9568.1997.tb01513.x

[72] Sánchez Martin C, Ledesma D, Dotti CG, et al. Microtubule-associated protein-2 located in growth regions of rat hippocampal neurons is highly phosphorylated at its proline-rich region. Neuroscience 2000;101:885–93. 11113337 10.1016/s0306-4522(00)00434-6

[73] Rodriguez-Jimenez FJ, Vilches A, Perez-Arago MA, et al. Activation of neurogenesis in multipotent stem cells cultured *in vitro* and in the spinal cord tissue after severe injury by inhibition of glycogen synthase kinase-3. Neurotherapeutics 2021;18:515–33. 33000422 10.1007/s13311-020-00928-0 PMC8116371

[74] De Simone A, La Pietra V, Betari N, et al. Discovery of the first-in-class GSK-3β/HDAC dual inhibitor as disease-modifying agent to combat Alzheimer’s disease. ACS Med Chem Lett 2019;10:469–74. 30996781 10.1021/acsmedchemlett.8b00507 PMC6466523

[75] Shi XL, Yan N, Cui YJ, et al. A unique GSK-3β inhibitor B10 has a direct effect on Aβ, targets tau and metal dyshomeostasis, and promotes neuronal neurite outgrowth. Cells 2020;9:649. 10.3390/cells9030649 PMC7140427 32155989

[76] Pettersen EF, Goddard TD, Huang CC, et al. UCSF Chimera-a visualization system for exploratory research and analysis. J Comput Chem 2004;25:1605–12. 15264254 10.1002/jcc.20084

[77] Maestro, version 11.9.011; Schrödinger, LLC, New York, NY, 2019.

