# Supplementary material for: Synthesis and biological evaluation of thieno[3,2-c]pyrazol-3-amine derivatives as potent glycogen synthase kinase 3β inhibitors for Alzheimer’s disease
Source: J Enzyme Inhib Med Chem. 2022 Jun 14;37(1):1724–36. doi: 10.1080/14756366.2022.2086867 (PMC9225722; doi:10.1080/14756366.2022.2086867)
Supplement: Supplemental Material [file IENZ_A_2086867_SM8206.pdf]

Synthesis and biological evaluation of thieno[3,2-*c*]pyrazol-3-amine derivatives as potent glycogen synthase kinase 3β inhibitors for Alzheimer's disease

Ning Yan[a], Xiao-Long Shi[a], Long-Qian Tang[a], De-Feng Wang[a], Xun Li[b]*, Chao Liu[a]* and Zhao-Peng Liu[a]*

[a]*Institute of Medicinal Chemistry, Key Laboratory of Chemical Biology (Ministry of Education), School of Pharmaceutical Sciences, Shandong University, Jinan, PR China;*

[b]*Institute of Materia Medica, Shandong First Medical University & Shandong Academy of Medical Sciences, Jinan 250117, PR China*

*Corresponding author

Zhao-Peng Liu (liuzhaop@sdu.edu.cn), School of Pharmaceutical Sciences, Shandong University, Jinan, Shandong 250012, China;

Chao Liu (chaoliu@sdu.edu.cn), School of Pharmaceutical Sciences, Shandong University, Jinan, Shandong 250012, China;

Xun Li (tjulx2004@sdu.edu.cn), Institute of Materia Medica, Shandong First Medical University & Shandong Academy of Medical Sciences, Jinan, Shandong 250117, China;

^{1}H, ^{13}C NMR, HPLC spectra for the synthesized compounds…………………2-24

Compound **16a**

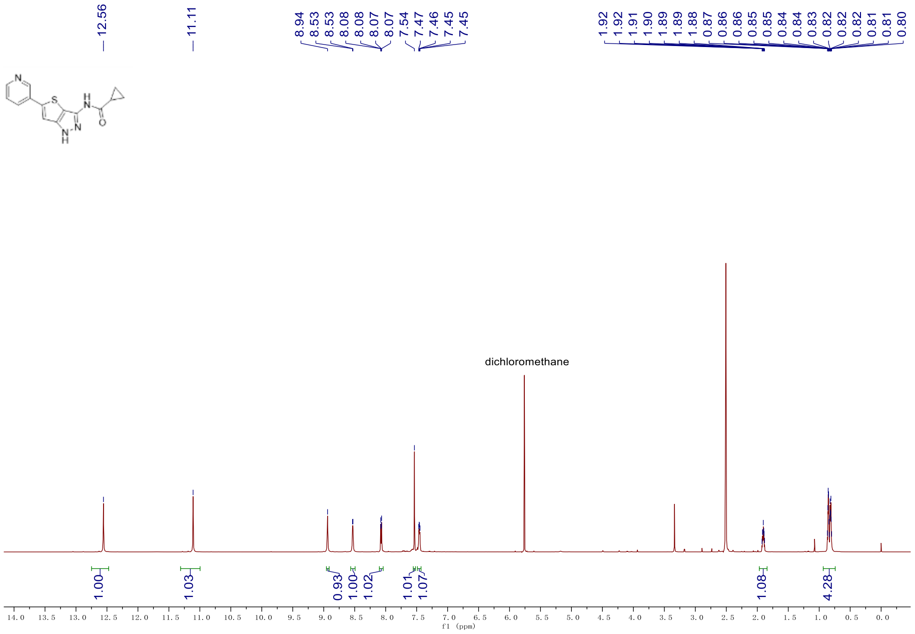

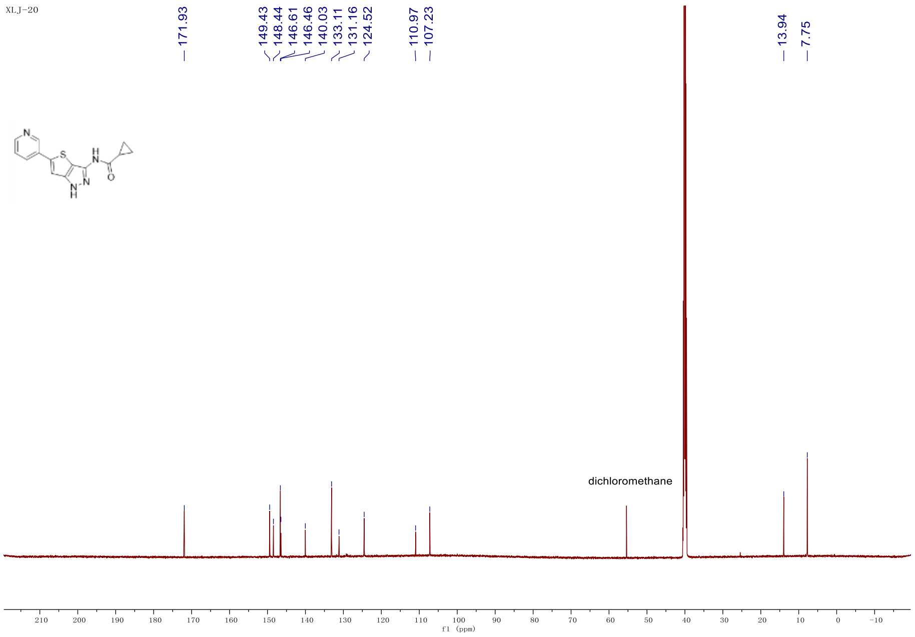

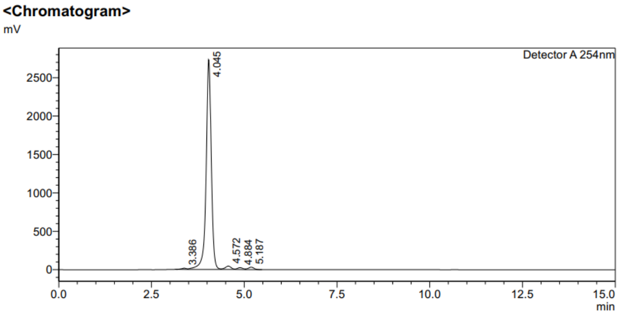

<Peak Table>

Detector A 254nm

Peak#	Ret. Time	Area	Height	Conc.
1	3.386	155890	16542	0.565
2	4.045	26253850	2731119	95.225
3	4.572	529888	44623	1.922
4	4.884	279915	25465	1.015
5	5.187	350890	32270	1.273
Total		27570433	2850019	

Compound **16b**

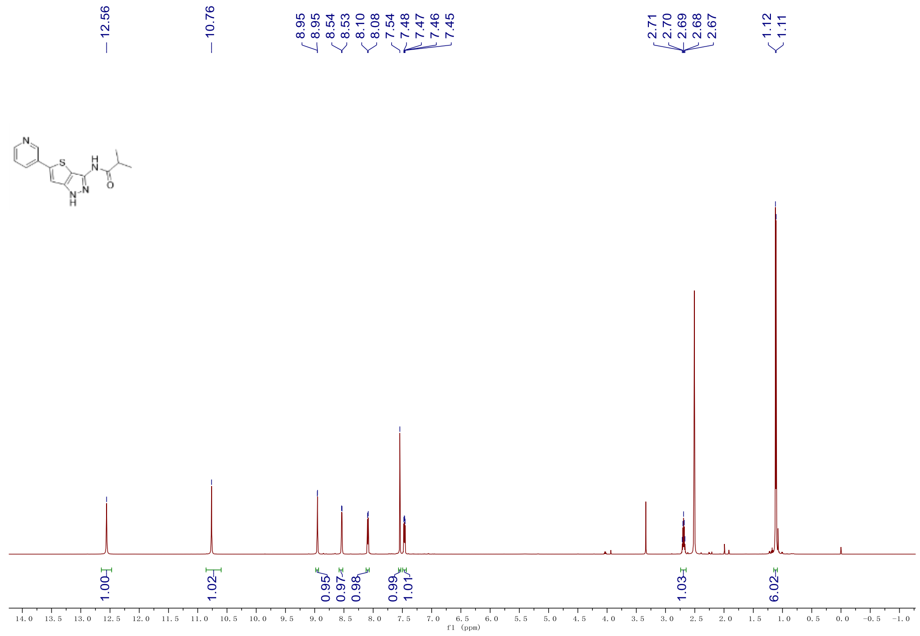

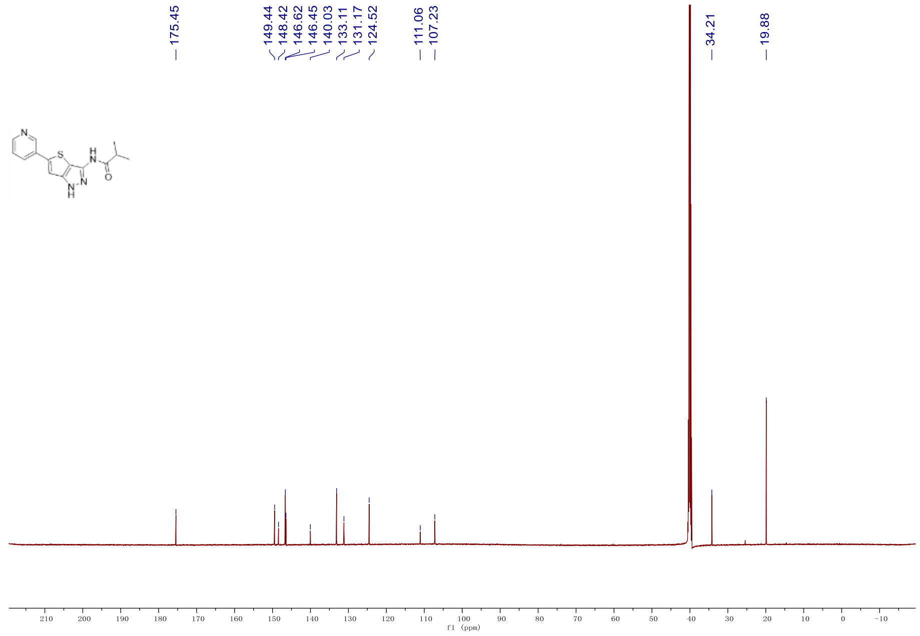

<Chromatogram>

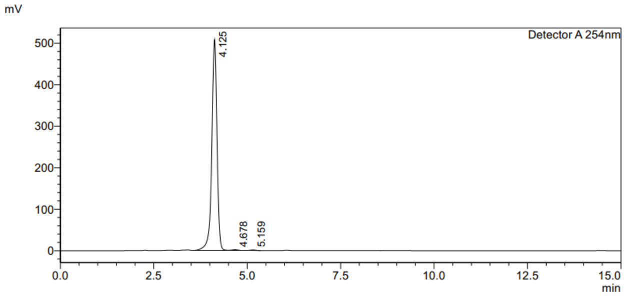

<Peak Table>

Detector A 254nm

Peak#	Ret. Time	Area	Height	Conc.	Unit
1	4.125	4853554	507859	99.318	
2	4.678	22210	1880	0.454	
3	5.159	11108	1249	0.227	
Total		4886872	510989		

Compound **16c**

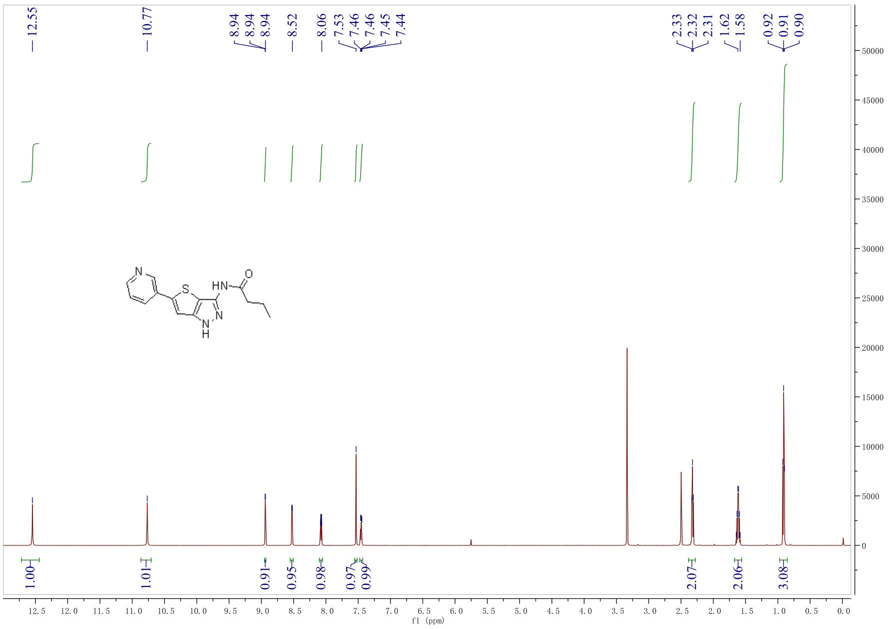

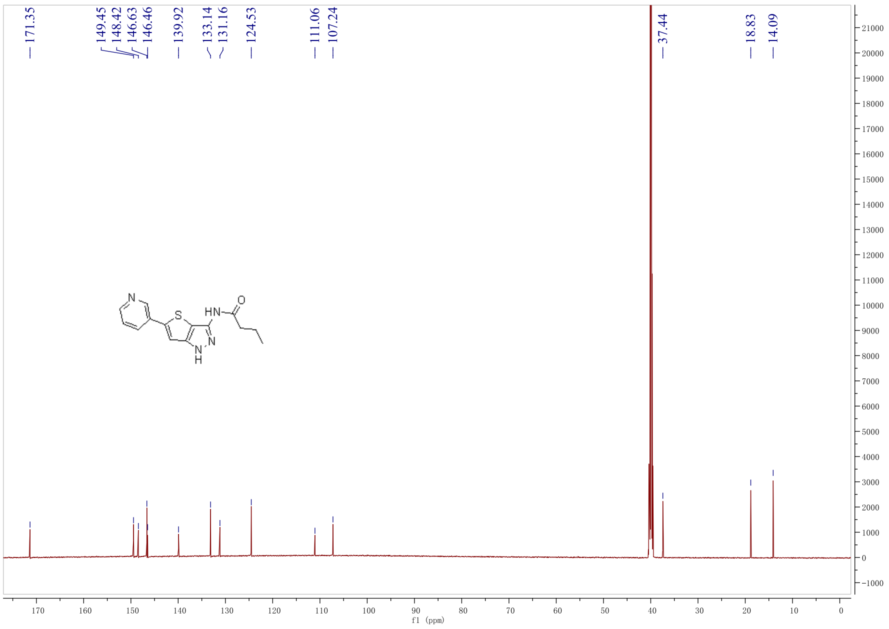

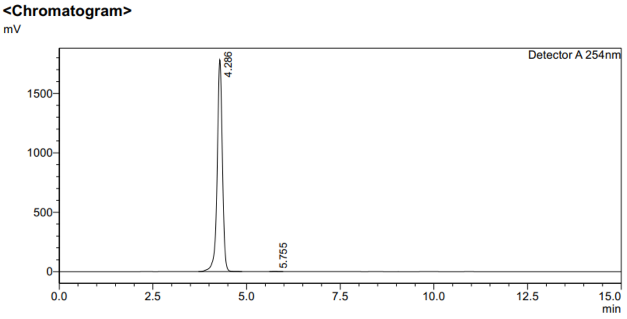

<Peak Table>

Detector A 254nm

Peak#	Ret. Time	Area	Height	Conc.	Unit
1	4.286	17299141	1780888	99.912	
2	5.755	15288	1505	0.088	
Total		17314430	1782393		

Compound **16d**

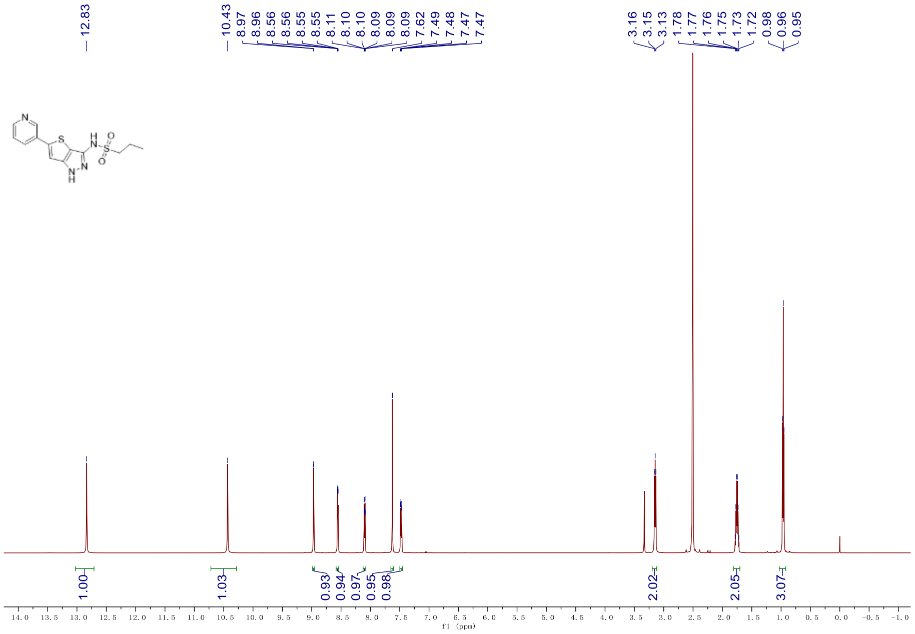

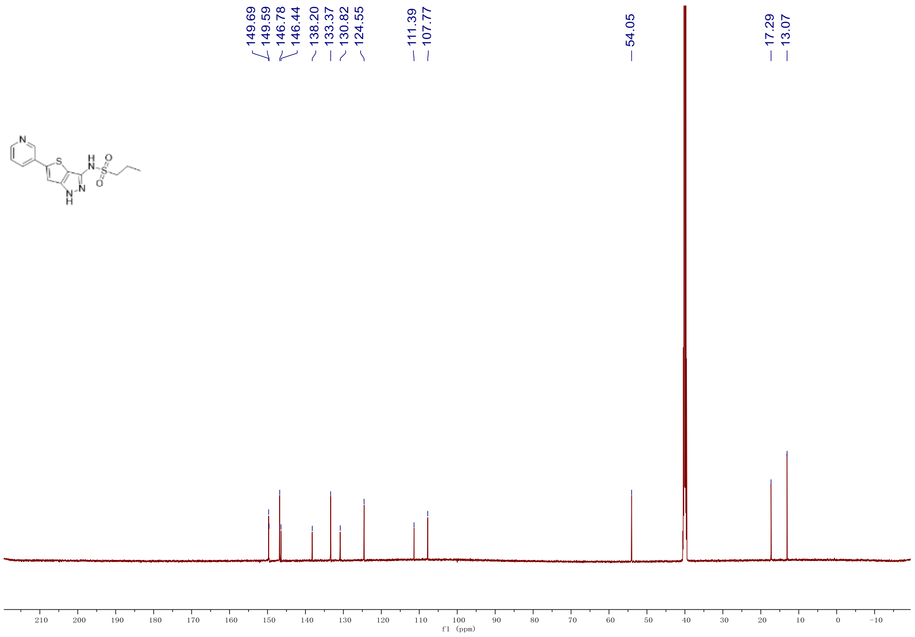

<Chromatogram>

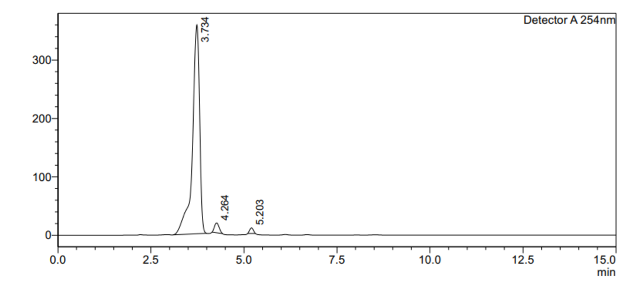

<Peak Table>

Detector A 254nm

Peak#	Ret. Time	Area	Height	Conc.	Unit
1	3.734	4848354	357488	95.824	
2	4.264	145993	16815	2.885	
3	5.203	65286	9495	1.290	
Total		5059633	383798		

Compound **16e**

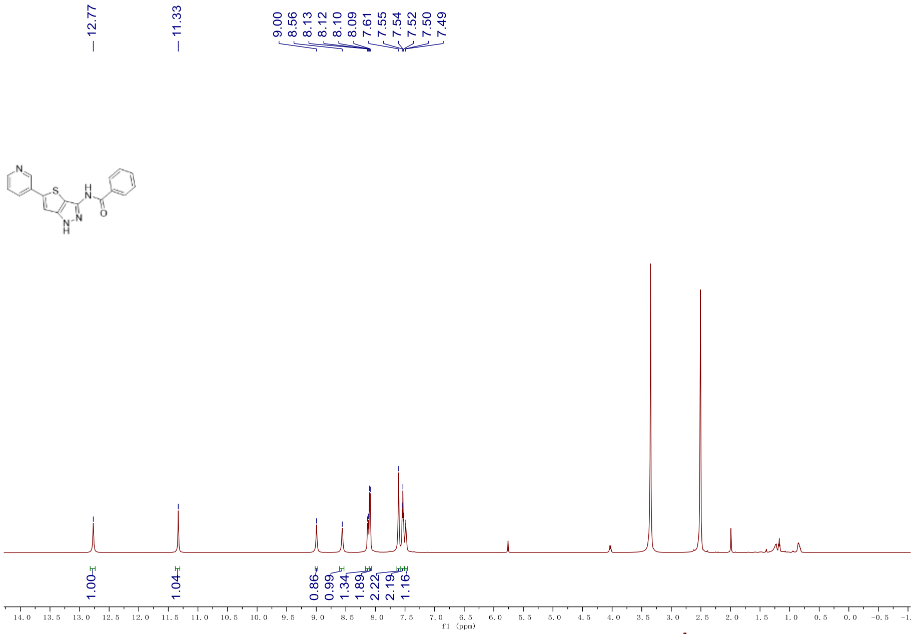

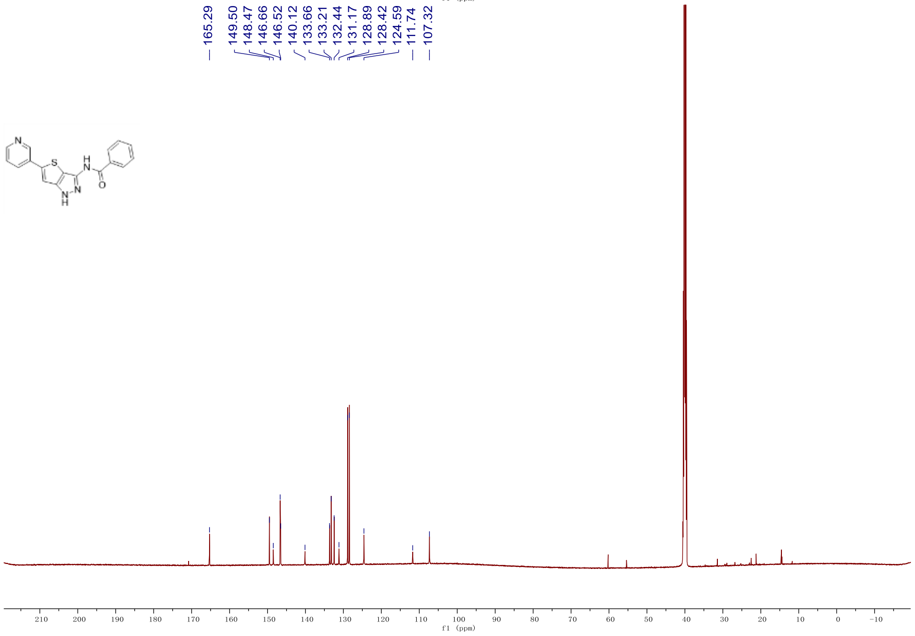

<Chromatogram>

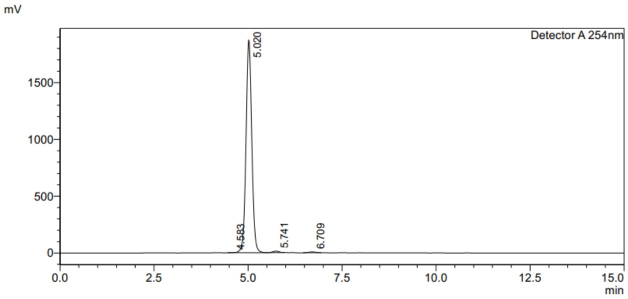

<Peak Table>

Detector A 254nm

Peak#	Ret. Time	Area	Height	Conc.	Unit
1	4.583	18550	2212	0.091	
2	5.020	20126555	1869984	98.697	
3	5.741	156461	13118	0.767	
4	6.709	90753	6862	0.445	
Total		20392319	1892177		

Compound **17a**

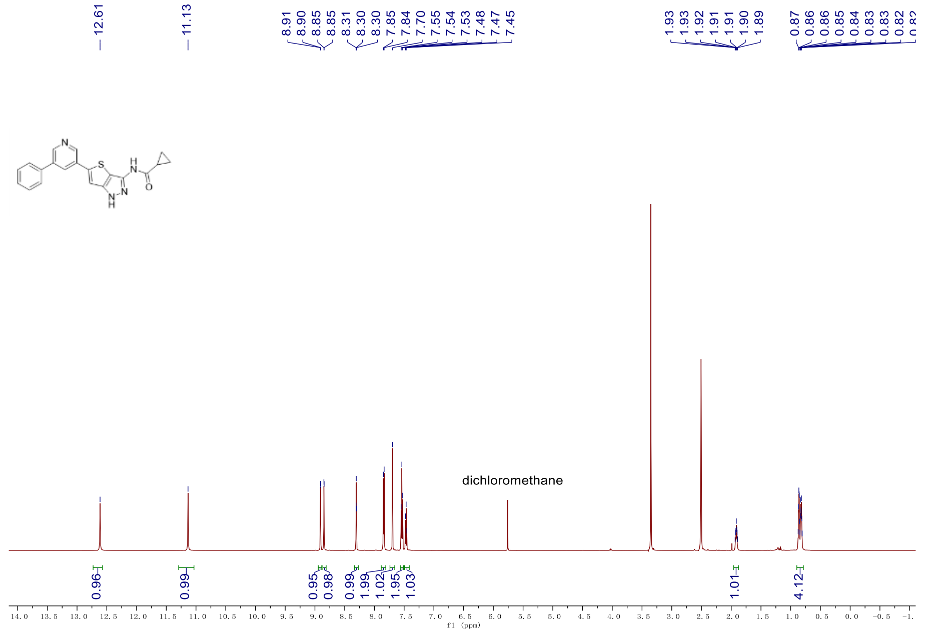

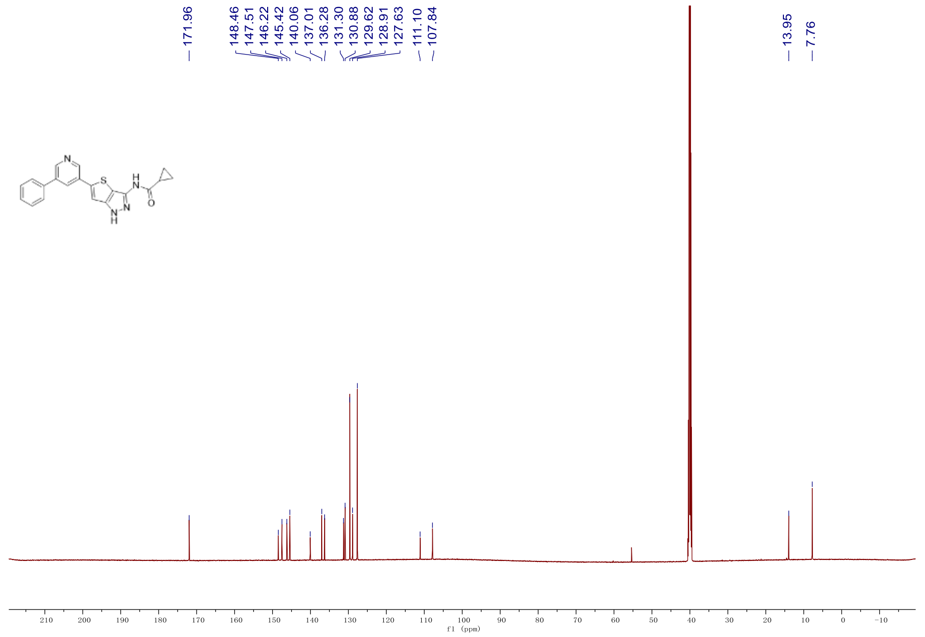

<Chromatogram>

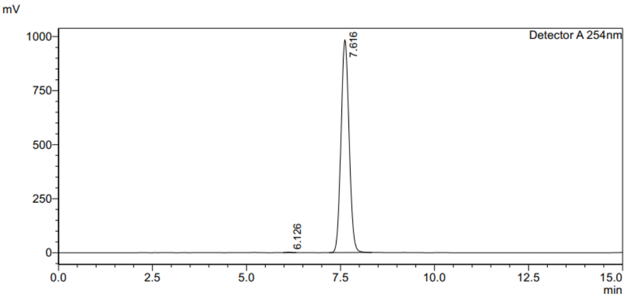

<Peak Table>

Detector A 254nm

Peak#	Ret. Time	Area	Height	Conc.	Unit
1	6.126	18870	2072	0.128	
2	7.616	14723693	982762	99.872	
Total		14742563	984834		

Compound **17b**

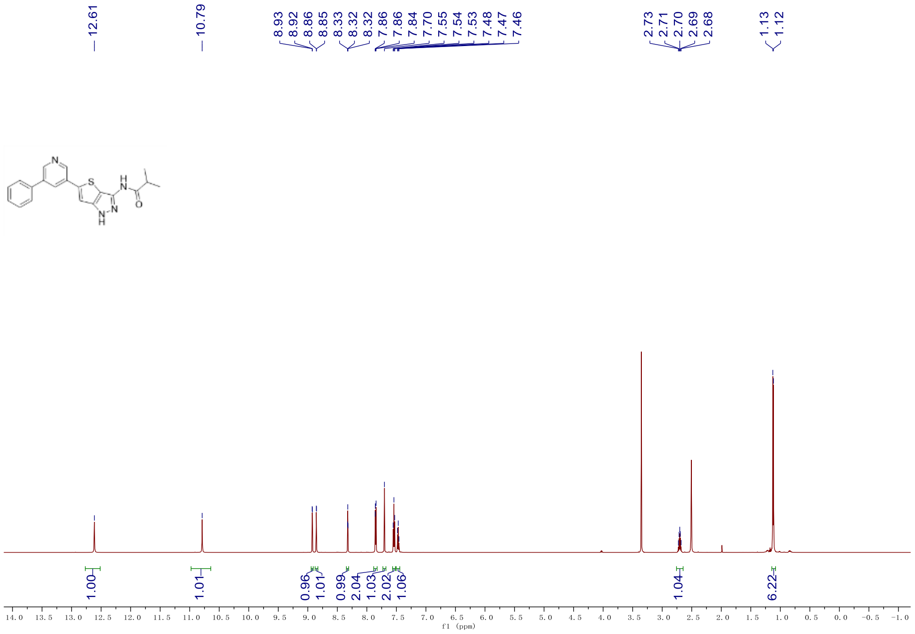

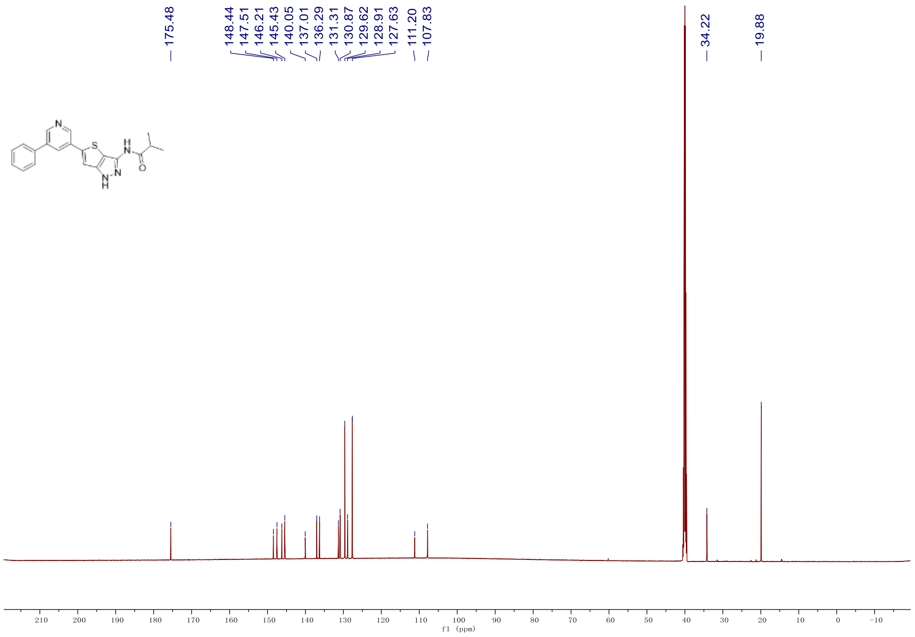

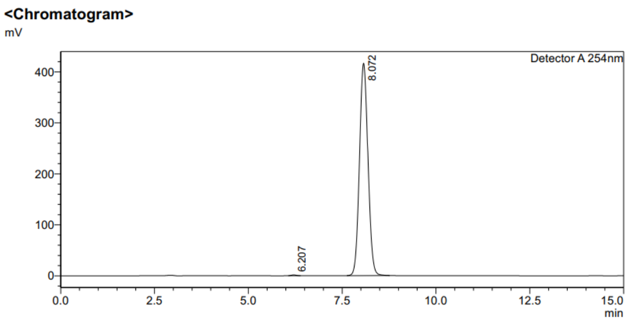

<Peak Table>

Detector A 254nm

Peak#	Ret. Time	Area	Height	Conc.	Unit
1	6.207	14331	1638	0.218	
2	8.072	6545220	416131	99.782	
Total		6559551	417769		

Compound **17c**

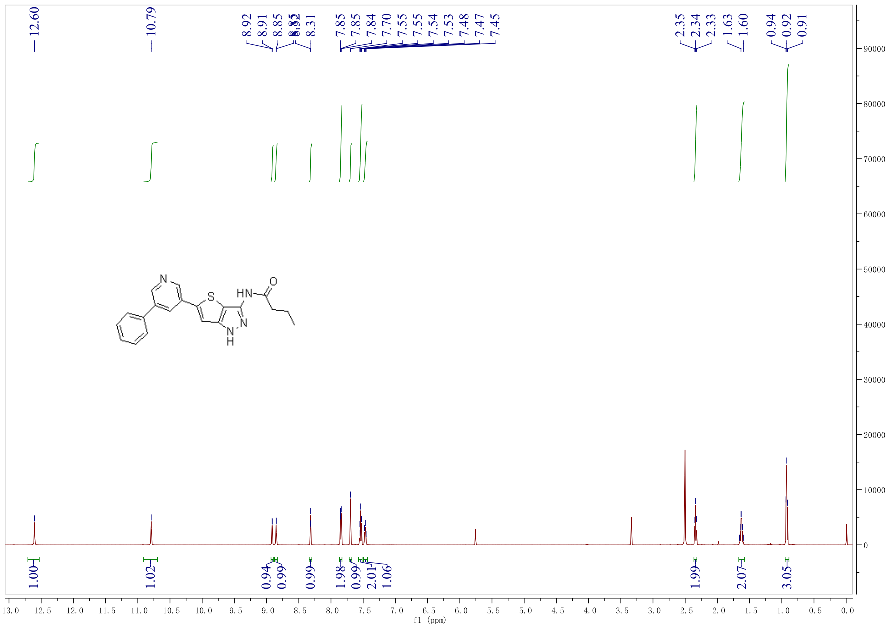

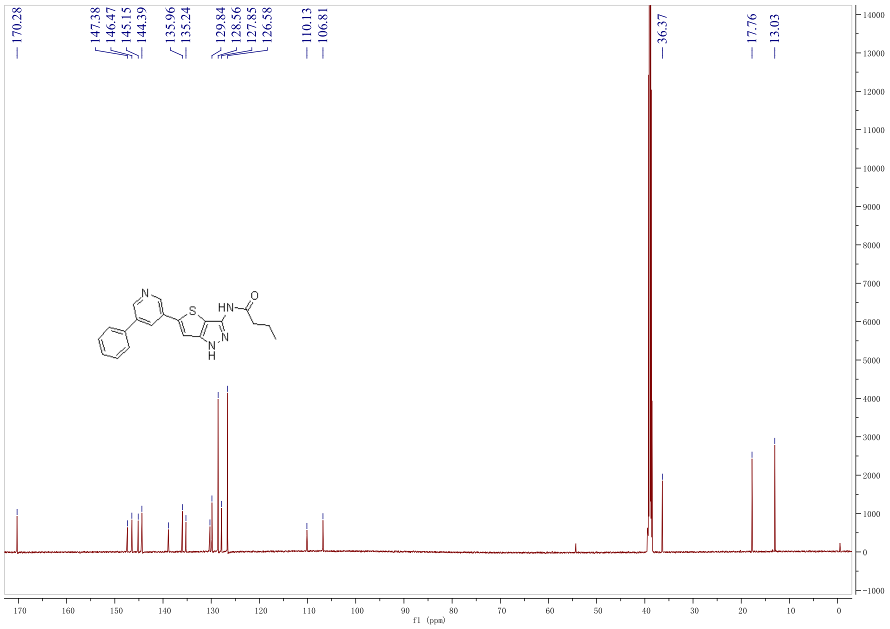

<Chromatogram>

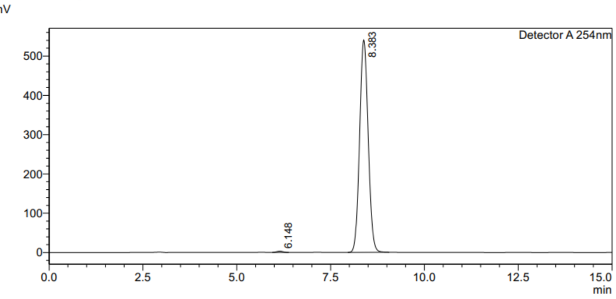

<Peak Table>

Detector A 254nm

Peak#	Ret. Time	Area	Height	Conc.	Unit
1	6.148	35083	3102	0.406	
2	8.383	8602346	540117	99.594	
Total		8637429	543219		

Compound **17d**

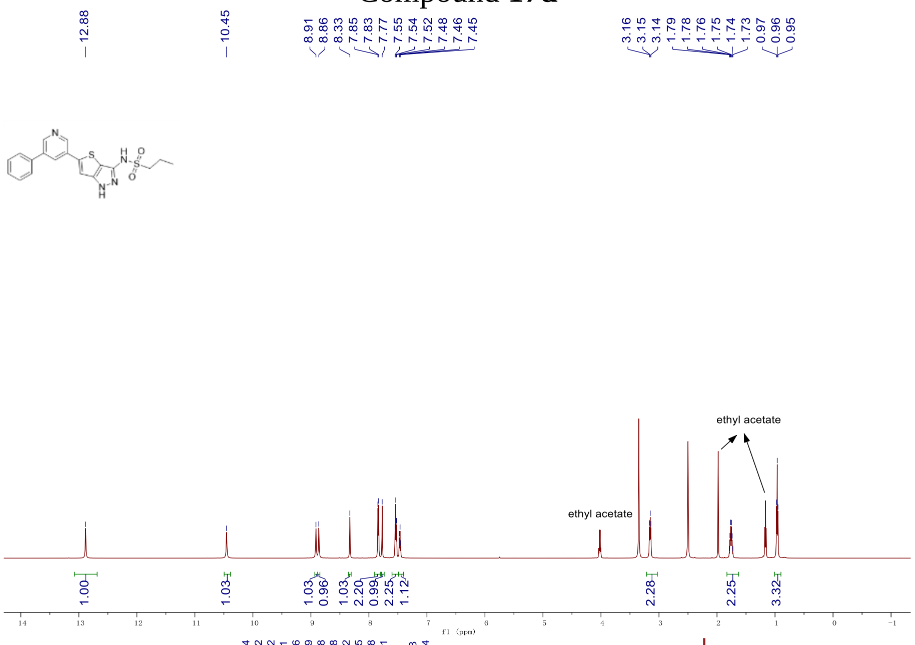

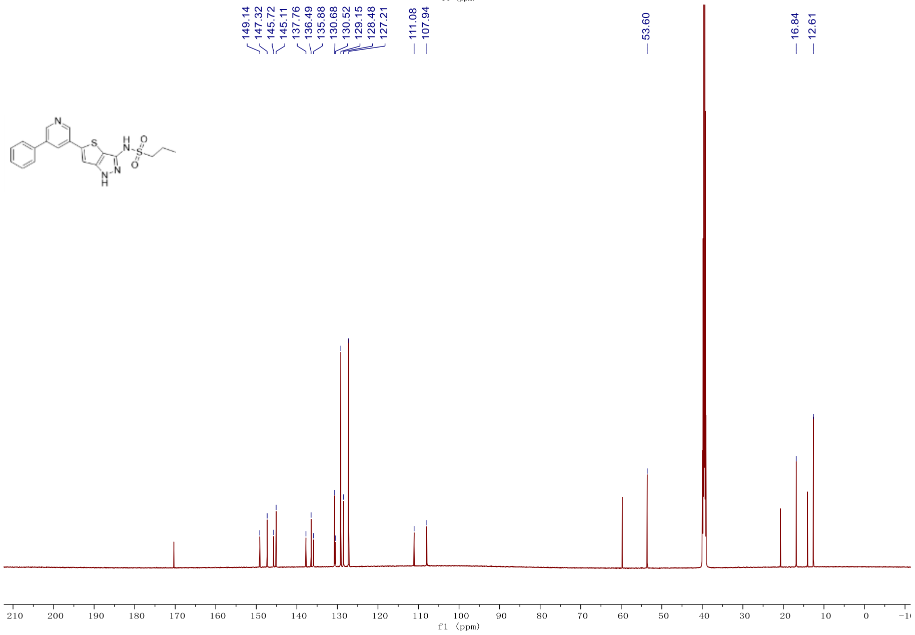

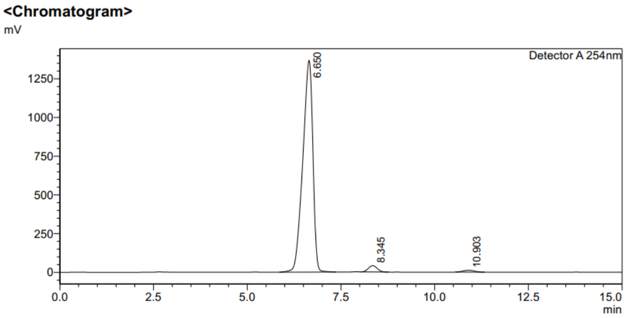

<Peak Table>

Detector A 254nm

Peak#	Ret. Time	Area	Height	Conc.	Unit
1	6.650	25621358	1366599	96.464	
2	8.345	679238	41204	2.557	
3	10.903	259892	11747	0.978	
Total		26560489	1419550		

Compound **18a**

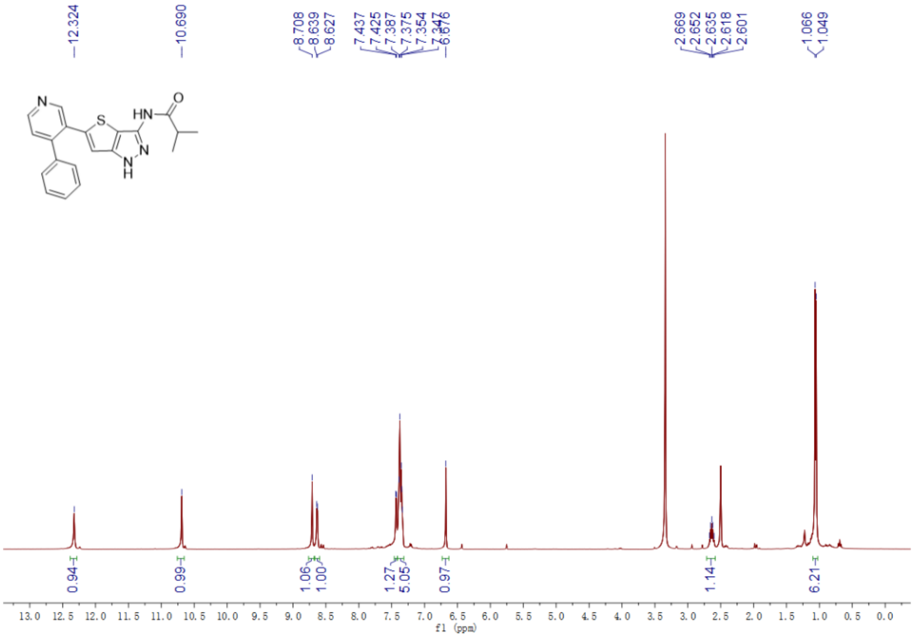

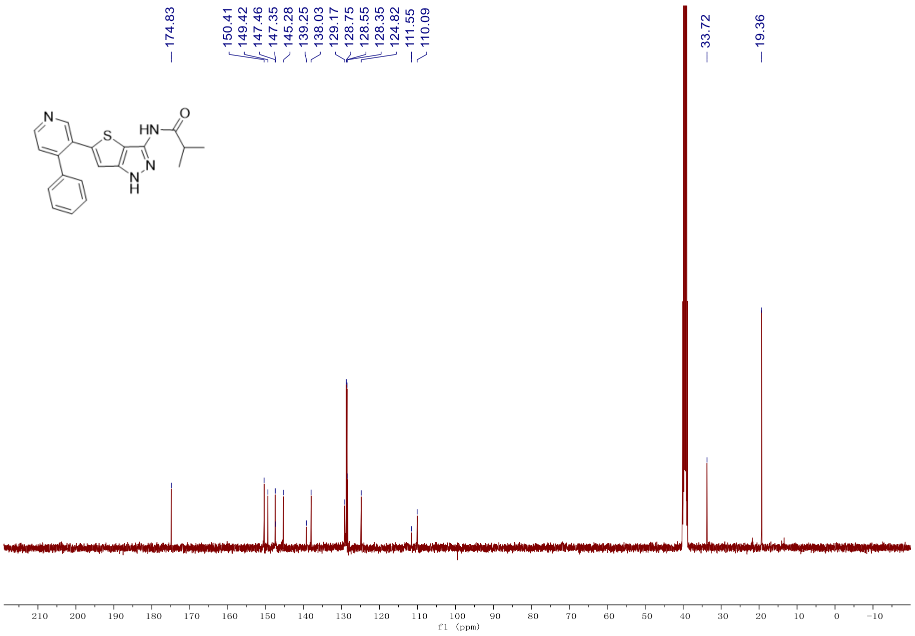

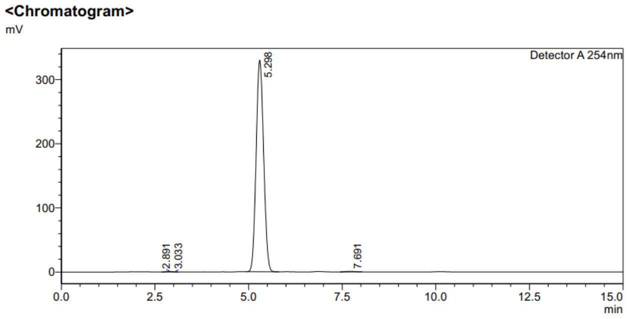

<Peak Table>

Detector A 254nm

Peak#	Ret. Time	Area	Height	Conc.	Unit
1	2.891	10953	958	0.237	
2	3.033	1596	418	0.035	
3	5.298	4590470	329748	99.349	
4	7.691	17527	971	0.379	
Total		4620545	332095		

Compound **18b**

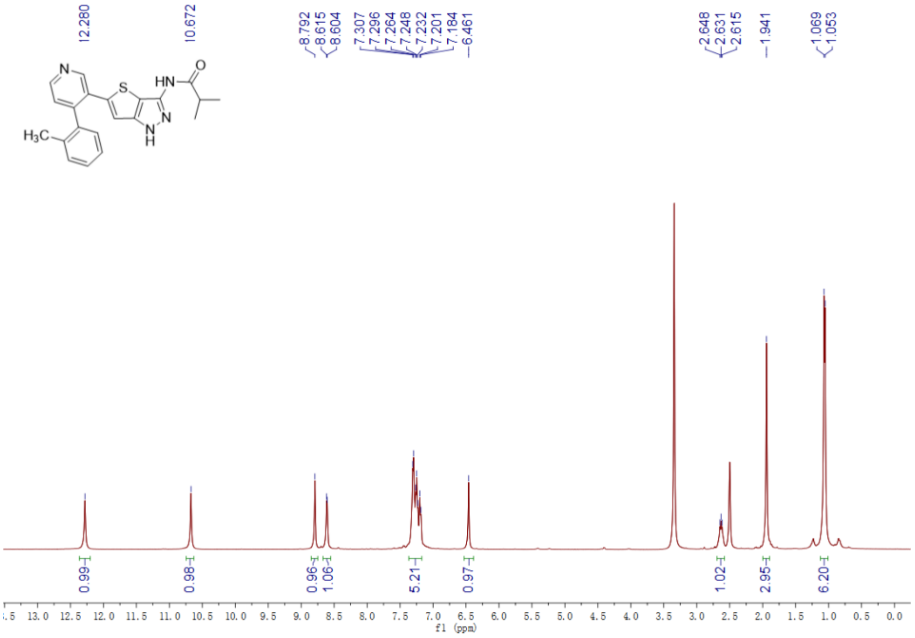

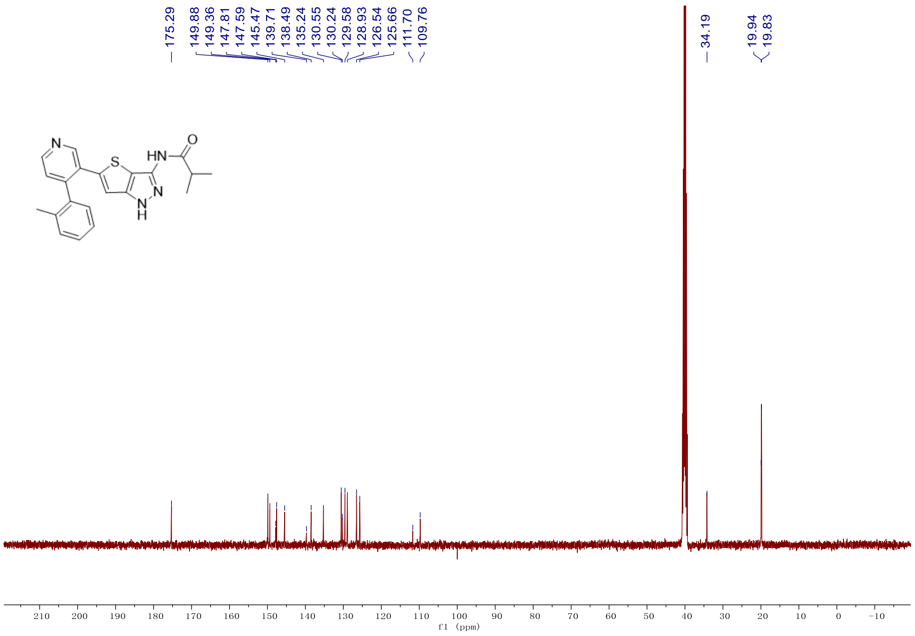

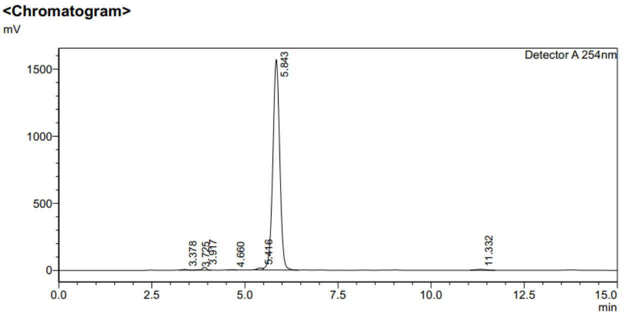

<Peak Table>

Detector A 254nm

Peak#	Ret. Time	Area	Height	Conc.	Unit
1	3.378	38483	5388	0.185	
2	3.725	24209	2981	0.116	
3	3.917	159389	20356	0.766	
4	4.660	18326	2169	0.088	
5	5.416	144961	14767	0.696	
6	5.843	20305430	1565855	97.553	
7	11.332	123956	6391	0.596	
Total		20814754	1617906		

Compound **18c**

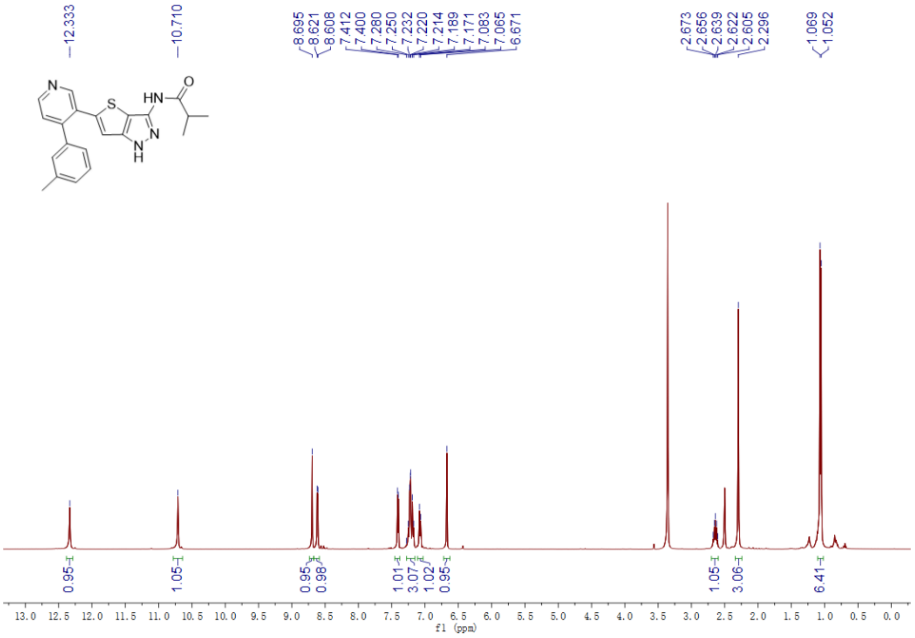

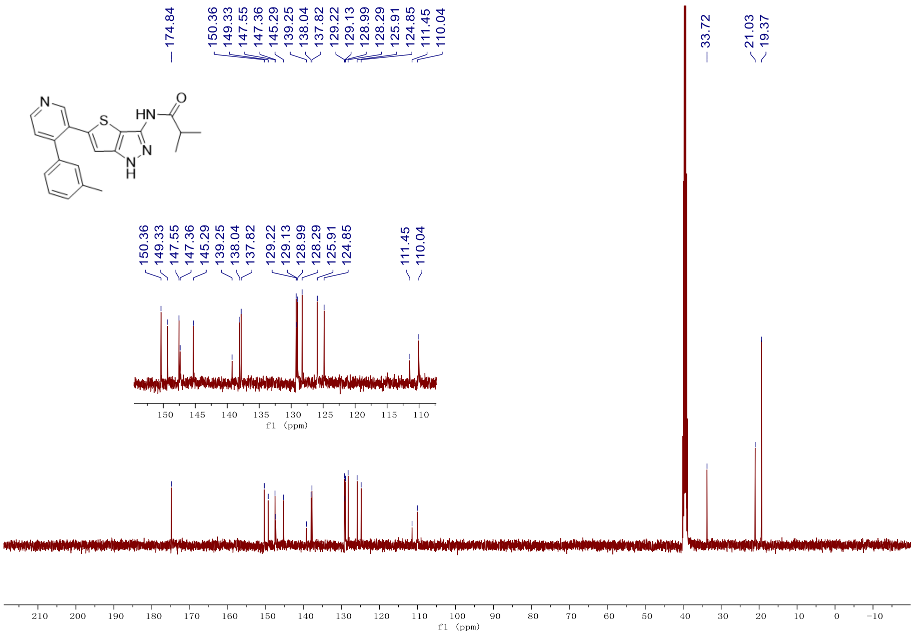

<Chromatogram>

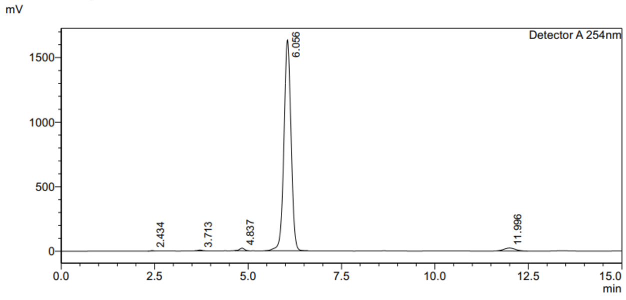

<Peak Table>

Detector A 254nm

Peak#	Ret. Time	Area	Height	Conc.	Unit
1	2.434	17959	2957	0.081	
2	3.713	50650	6779	0.228	
3	4.837	184991	20394	0.832	
4	6.056	21484788	1632499	96.644	
5	11.996	492554	22753	2.216	
Total		22230941	1685381		

Compound **18d**

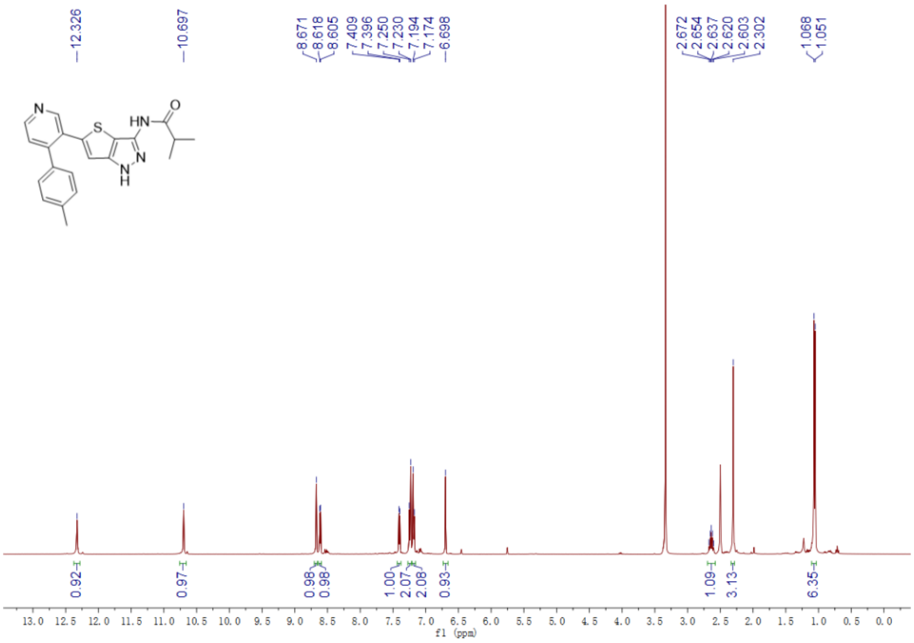

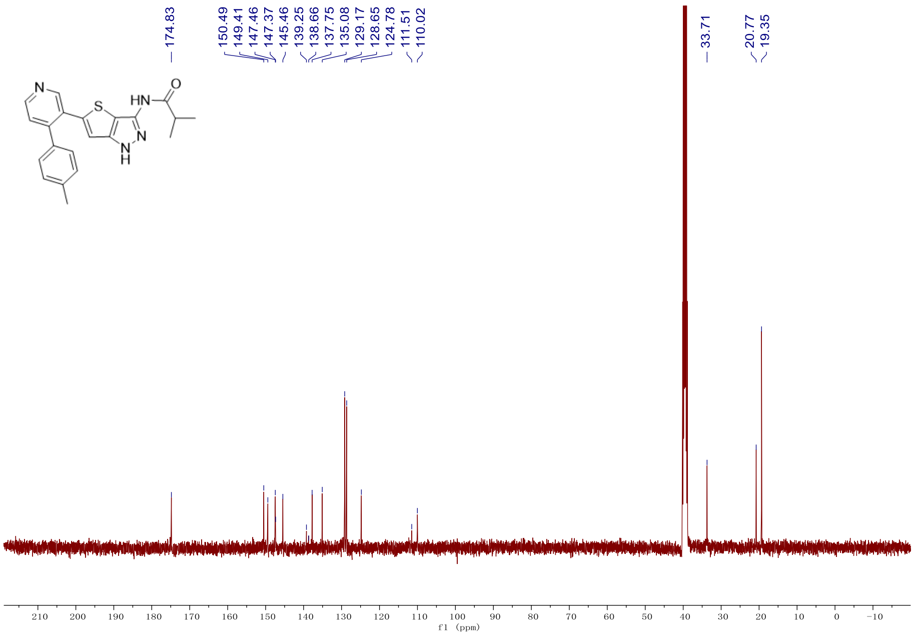

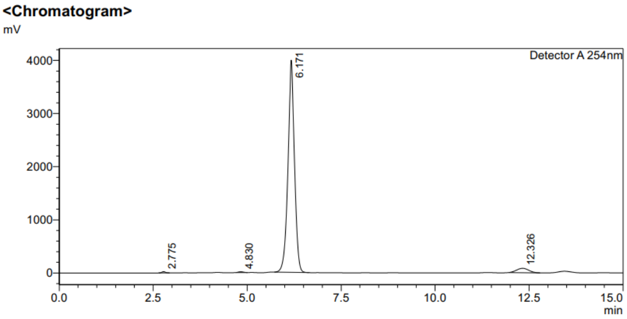

<Peak Table>

Detector A 254nm

Peak#	Ret. Time	Area	Height	Conc.	Unit
1	2.775	159250	23990	0.322	
2	4.830	129065	14248	0.261	
3	6.171	47308979	3988395	95.725	
4	12.326	1824549	82275	3.692	
Total		49421843	4108907		

Compound **18e**

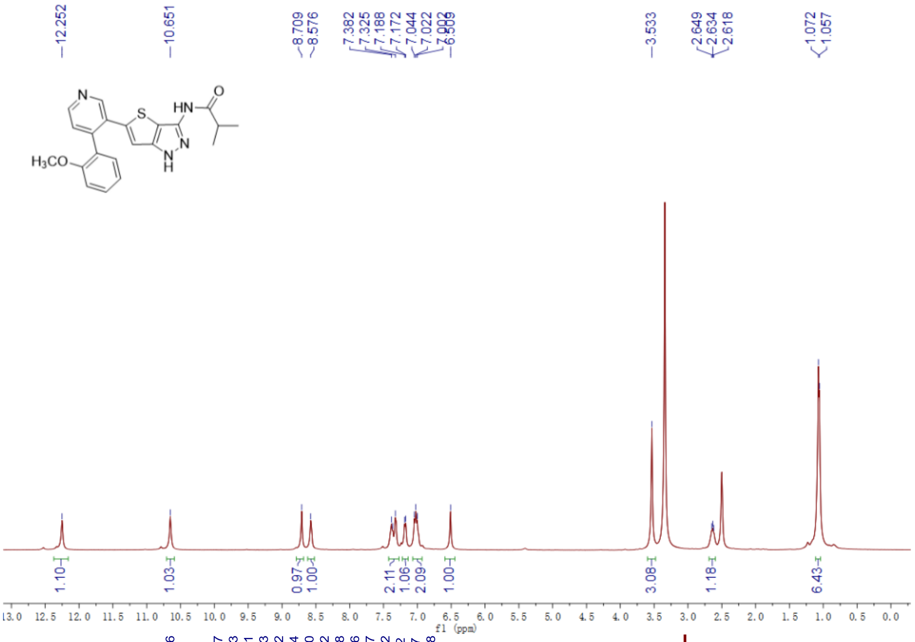

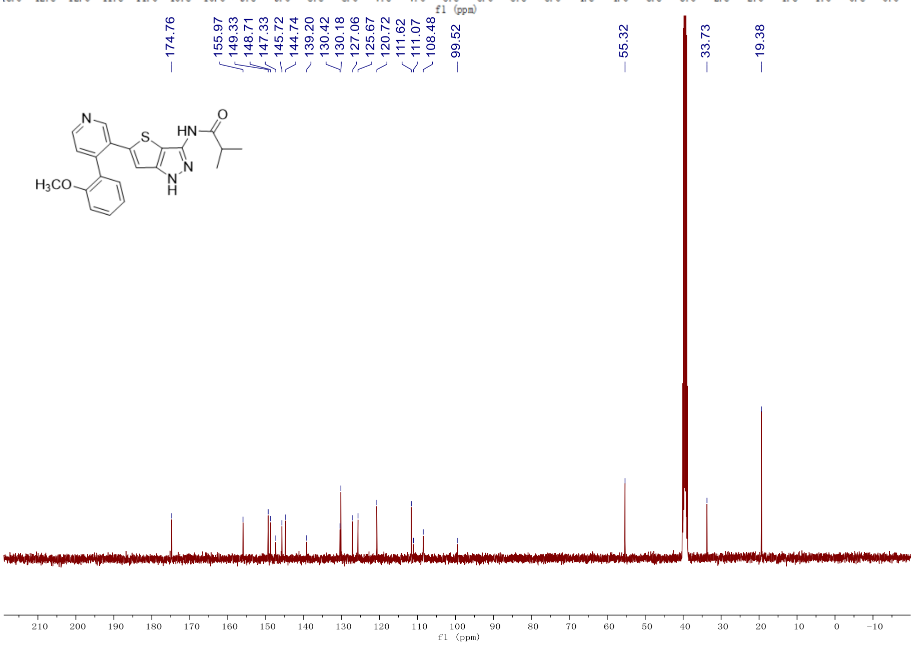

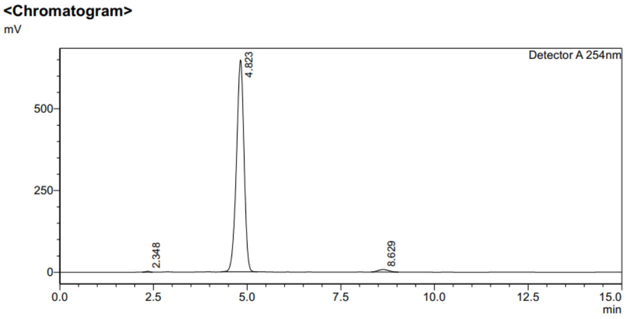

<Peak Table>

Detector A 254nm

Peak#	Ret. Time	Area	Height	Conc.	Unit
1	2.348	18611	2817	0.201	
2	4.823	9103747	647676	98.112	
3	8.629	156599	7804	1.688	
Total		9278957	658297		

Compound **18f**

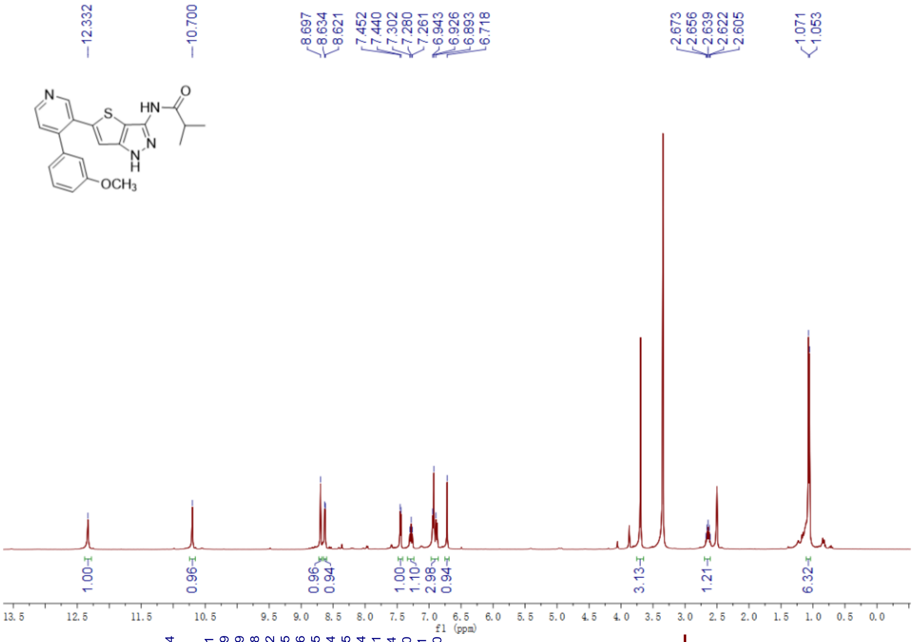

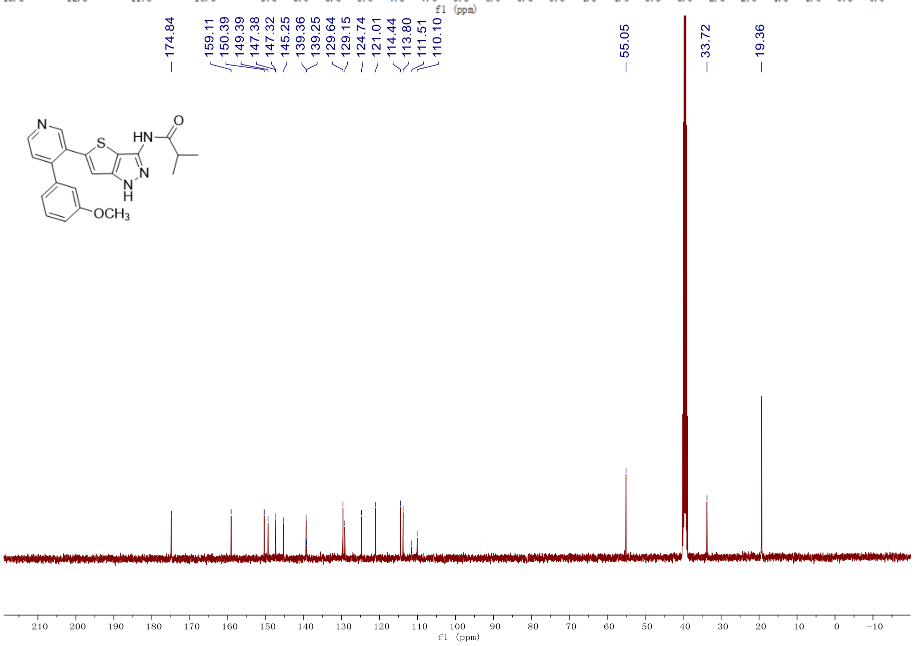

<Chromatogram>

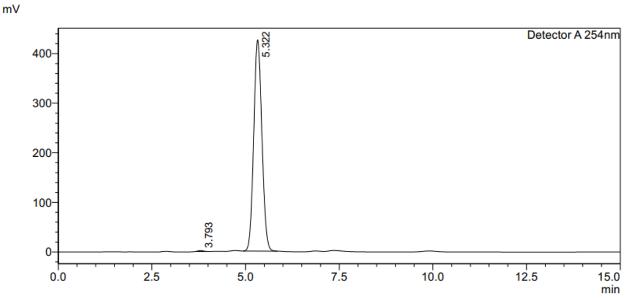

<Peak Table>

Detector A 254nm

Peak#	Ret. Time	Area	Height	Conc.	Unit
1	3.793	19499	1969	0.300	
2	5.322	6482714	425888	99.700	
Total		6502213	427858		

Compound **18g**

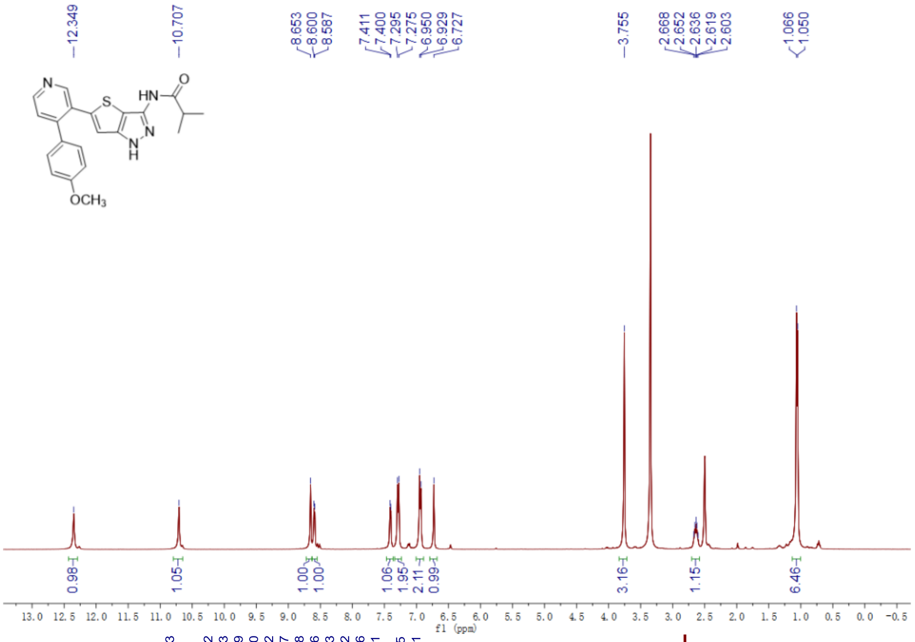

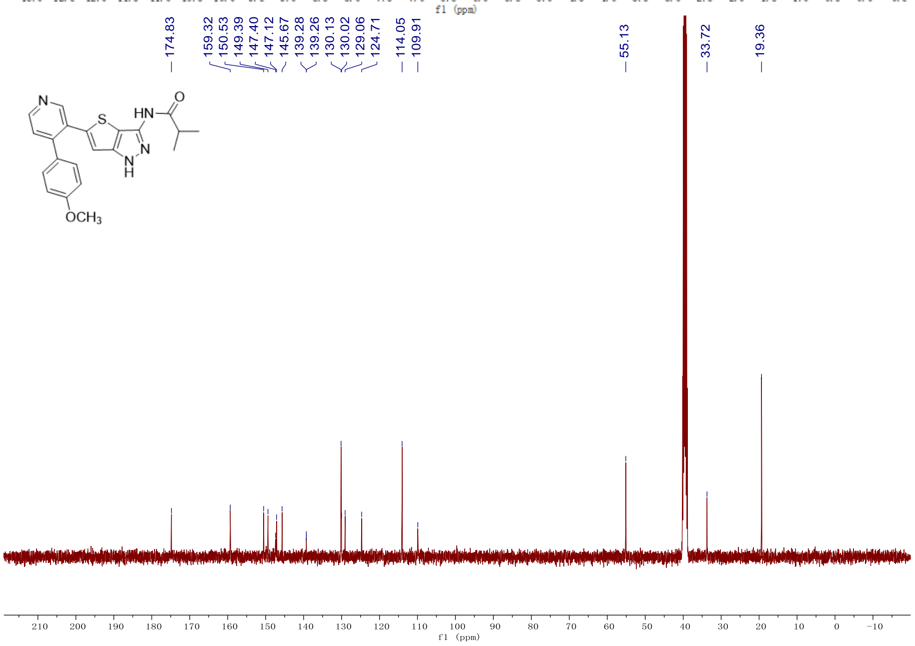

<Chromatogram>

mV

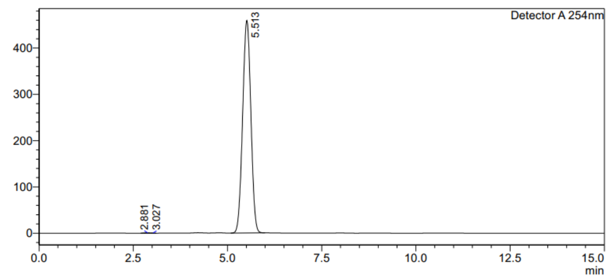

<Peak Table>

Detector A 254nm

Peak#	Ret. Time	Area	Height	Conc.	Unit
1	2.881	10812	1006	0.154	
2	3.027	1877	414	0.027	
3	5.513	6985636	459219	99.819	
Total		6998325	460639		

Compound **18h**

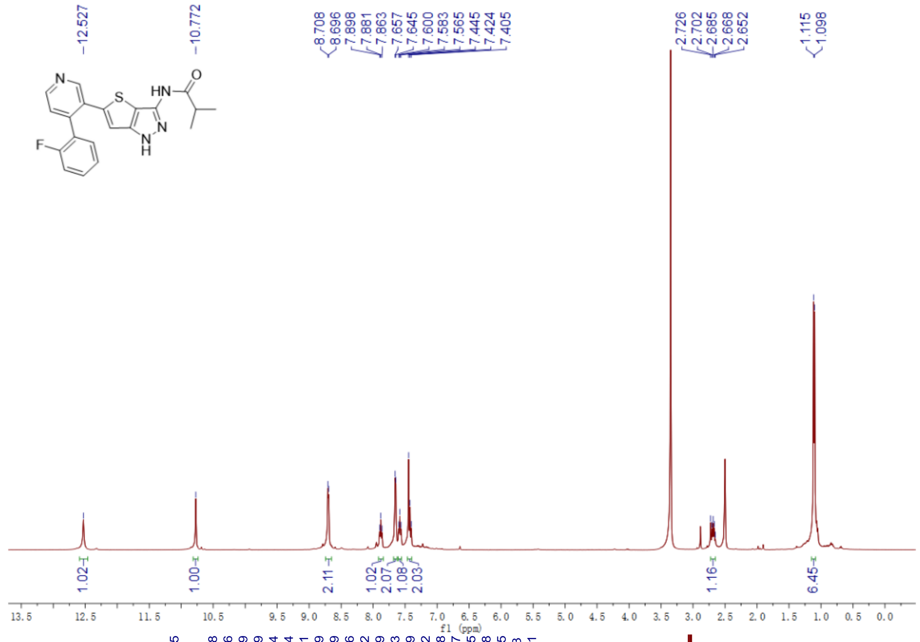

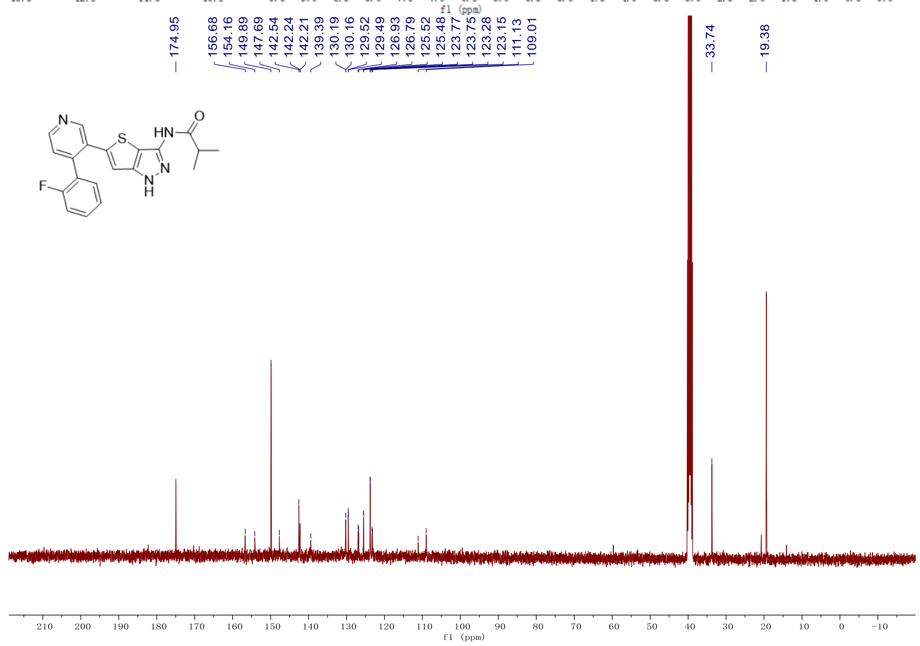

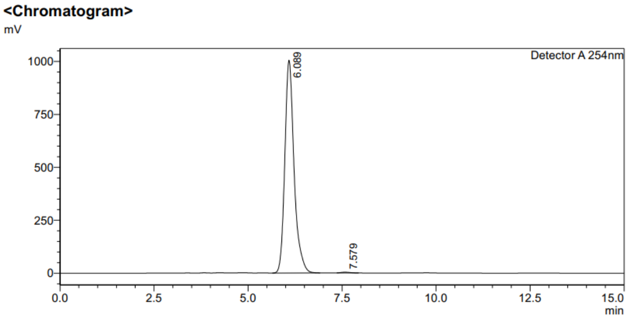

<Peak Table>

Detector A 254nm

Peak#	Ret. Time	Area	Height	Conc.	Unit
1	6.089	17209598	1004173	99.682	
2	7.579	54970	3376	0.318	
Total		17264569	1007549		

Compound **18i**

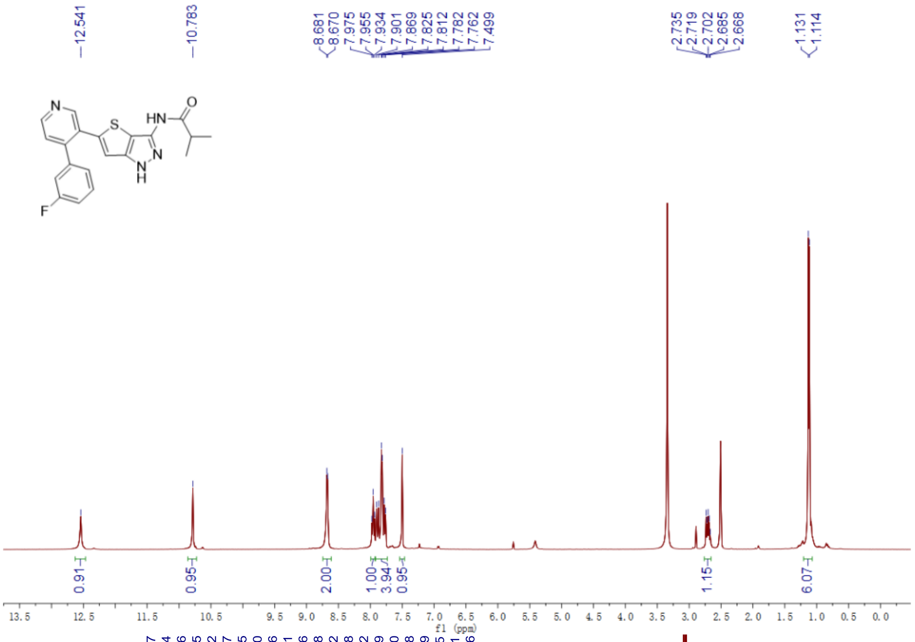

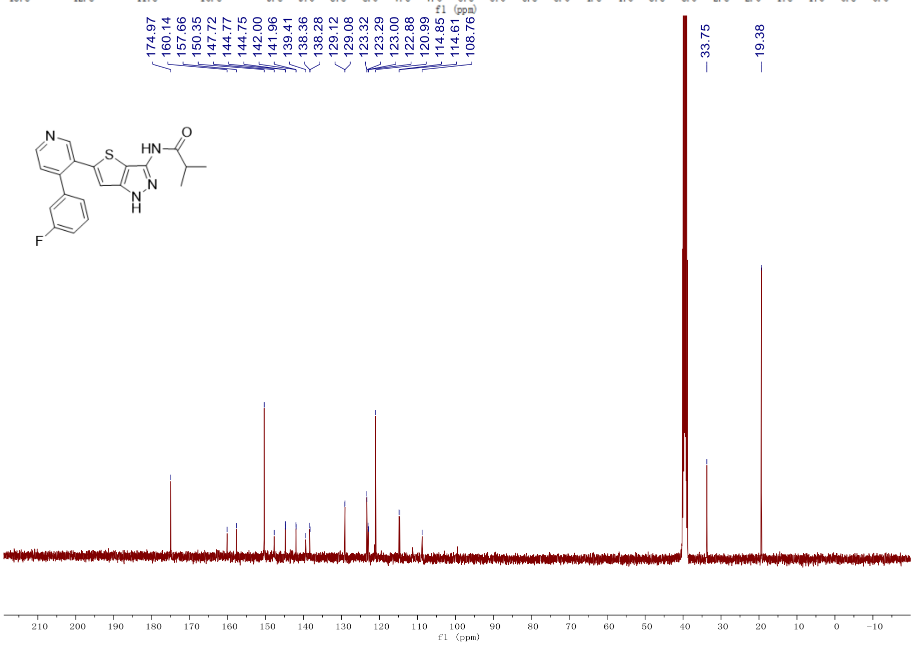

<Chromatogram>

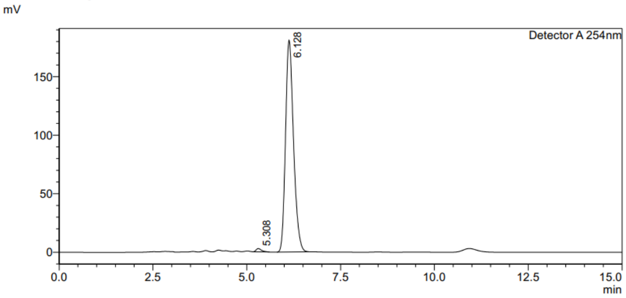

<Peak Table>

Detector A 254nm

Peak#	Ret. Time	Area	Height	Conc.	Unit
1	5.308	28297	2801	1.044	
2	6.128	2681380	180764	98.956	
Total		2709676	183565		

Compound **18j**

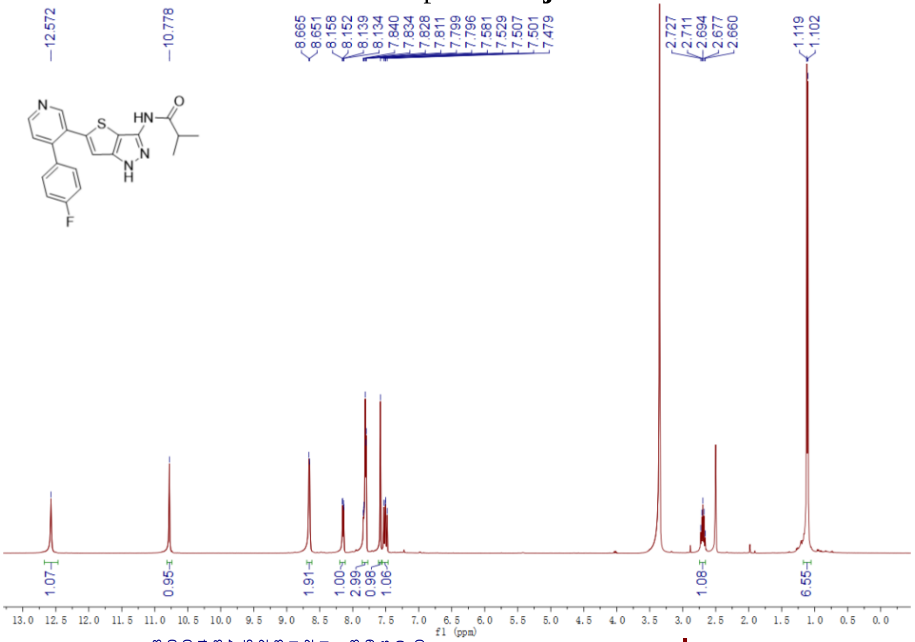

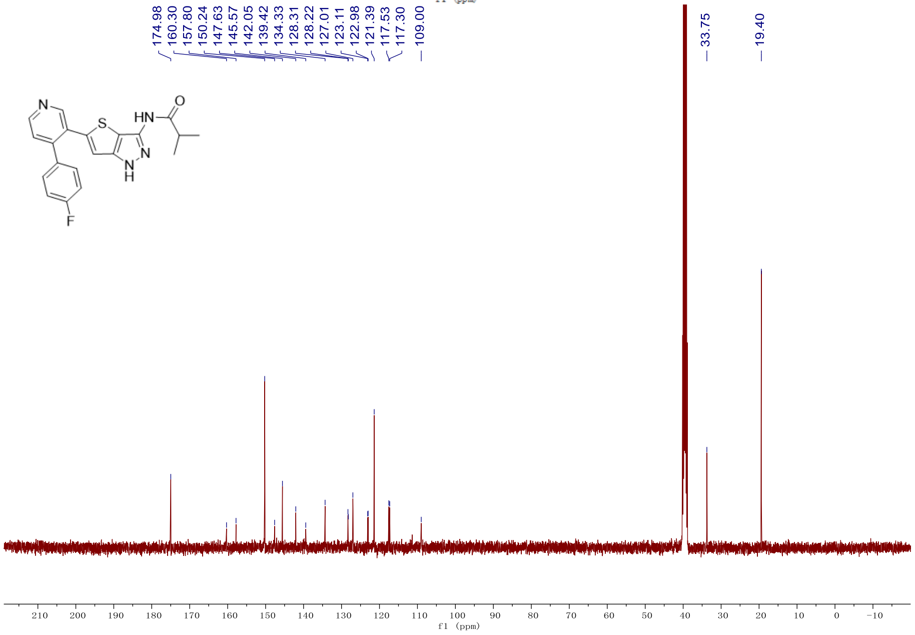

<Chromatogram>

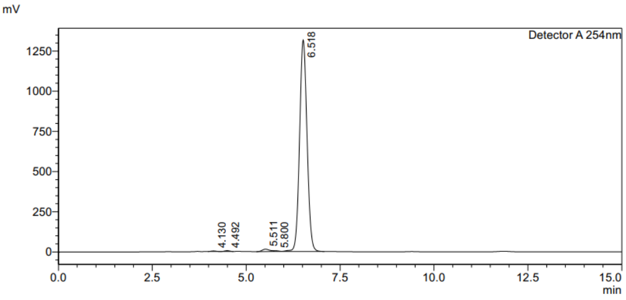

<Peak Table>

Detector A 254nm

Peak#	Ret. Time	Area	Height	Conc.	Unit
1	4.130	30377	3345	0.163	
2	4.492	49016	5336	0.263	
3	5.511	217774	15383	1.167	
4	5.800	49573	5276	0.266	
5	6.518	18314682	1315830	98.142	
Total		18661422	1345168		

Compound **18k**

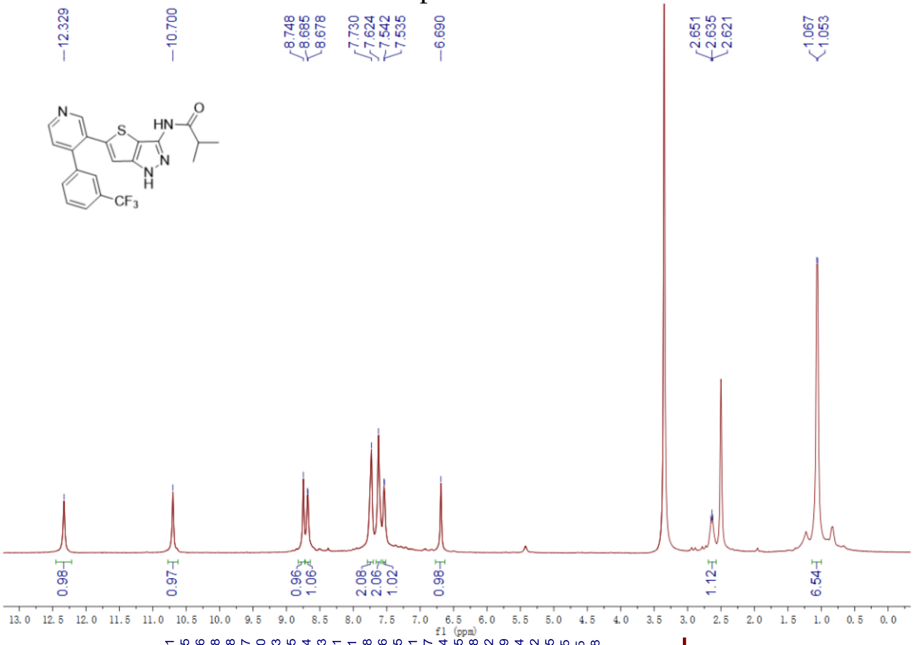

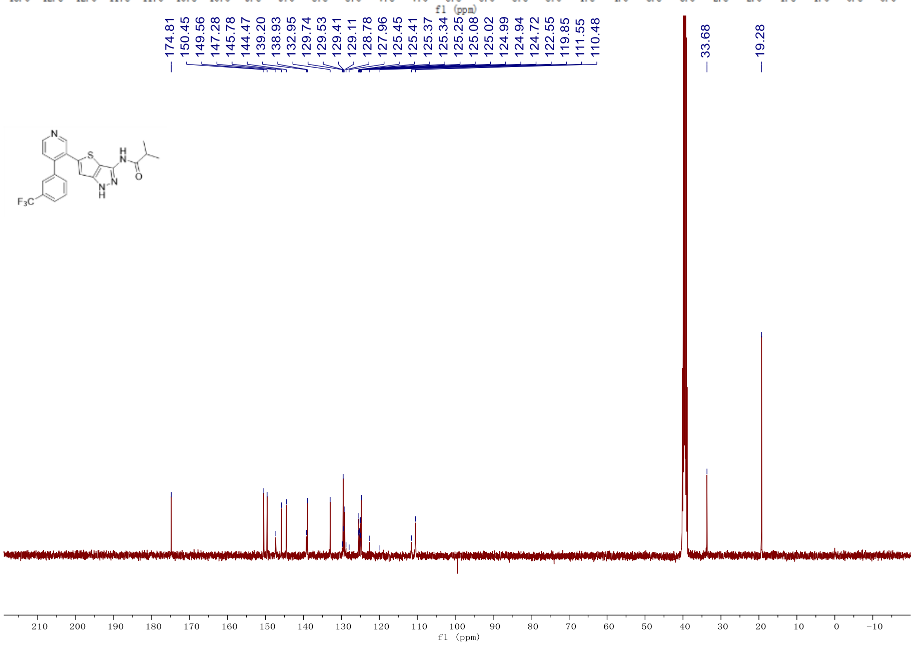

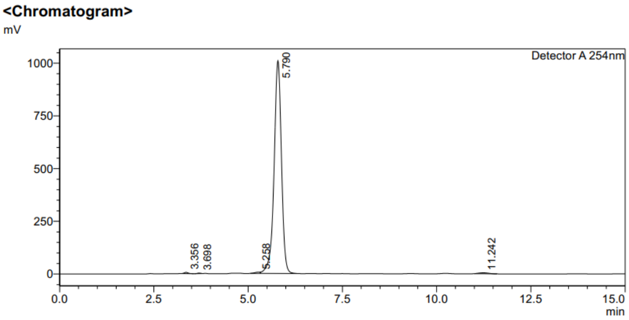

<Peak Table>

Detector A 254nm

Peak#	Ret. Time	Area	Height	Conc.	Unit
1	3.356	43268	6218	0.308	
2	3.698	13891	2253	0.099	
3	5.258	60238	6029	0.429	
4	5.790	13828493	1009234	98.584	
5	11.242	81163	4550	0.579	
Total		14027052	1028284		

Compound **18l**

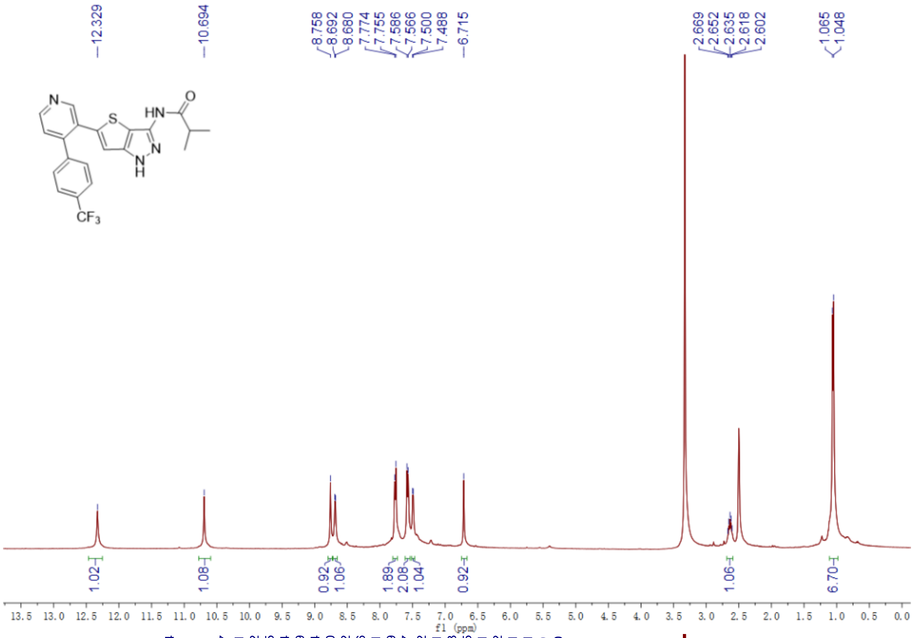

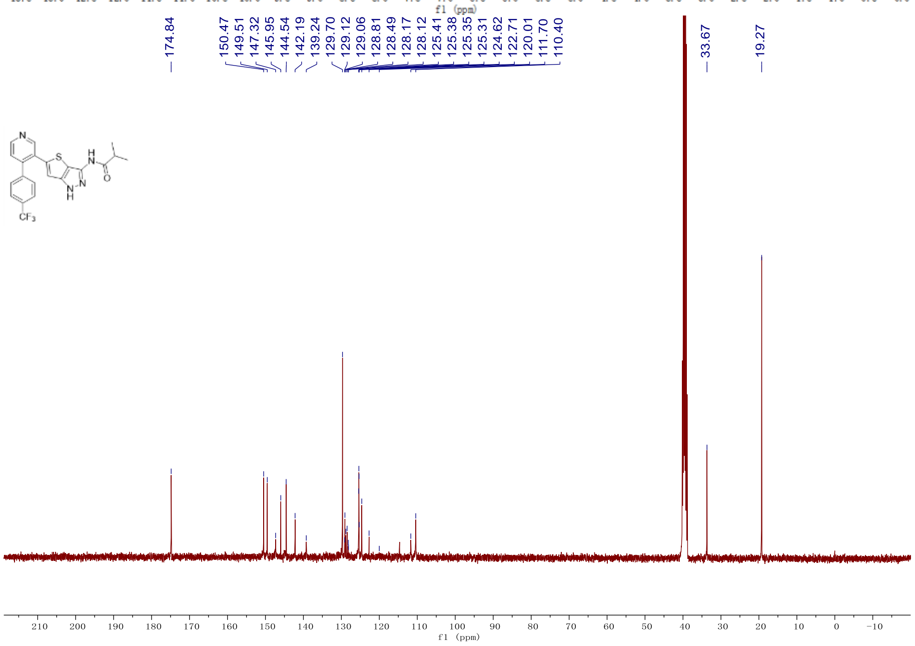

<Chromatogram>

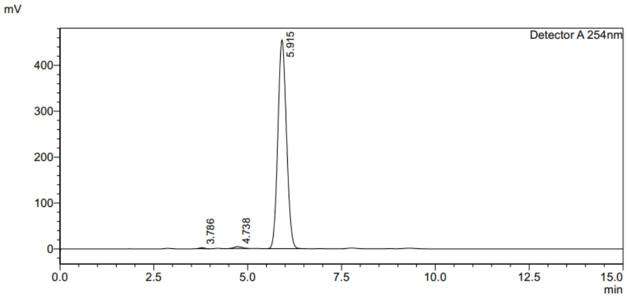

<Peak Table>

Detector A 254nm

Peak#	Ret. Time	Area	Height	Conc.	Unit
1	3.786	19815	2274	0.270	
2	4.738	59063	4060	0.806	
3	5.915	7249680	454338	98.924	
Total		7328558	460672		

Compound **18m**

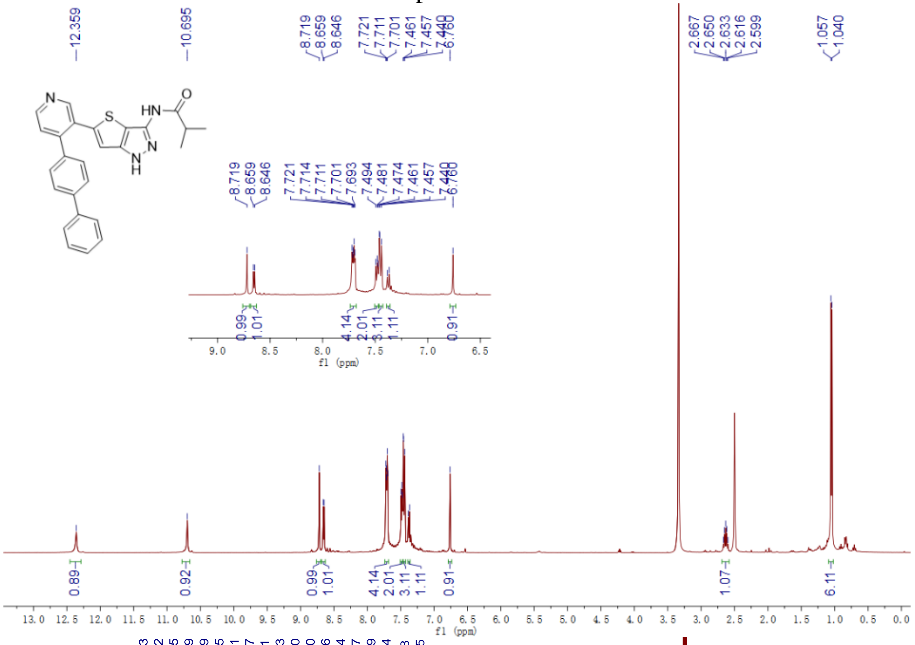

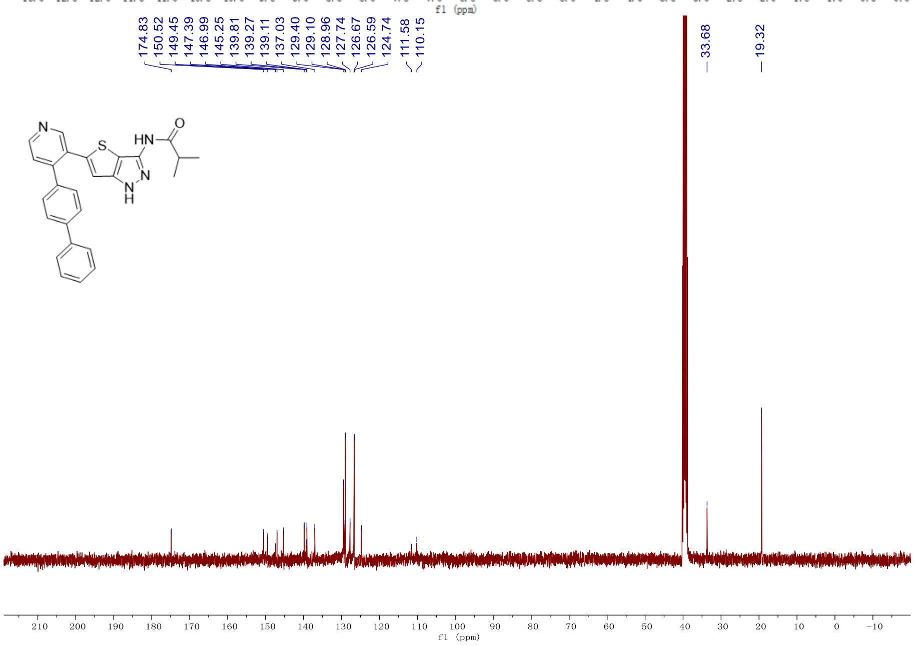

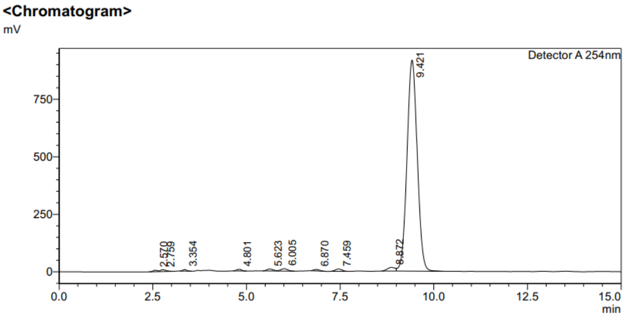

<Peak Table>

Detector A 254nm

Peak#	Ret. Time	Area	Height	Conc.	Unit
1	2.570	57744	6287	0.315	
2	2.759	86829	8152	0.474	
3	3.354	45428	6676	0.248	
4	4.801	64587	7069	0.353	
5	5.623	100753	8259	0.550	
6	6.005	131691	10232	0.719	
7	6.870	66068	6152	0.361	
8	7.459	127371	10141	0.695	
9	8.872	226610	15594	1.237	
10	9.421	17408527	916533	95.047	
Total		18315607	995095		

Compound **18n**

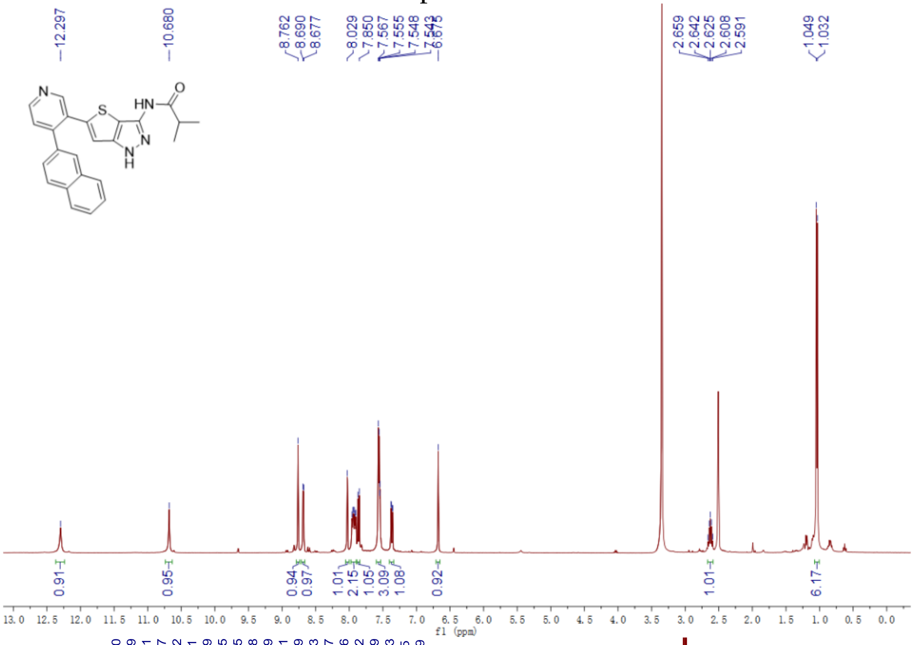

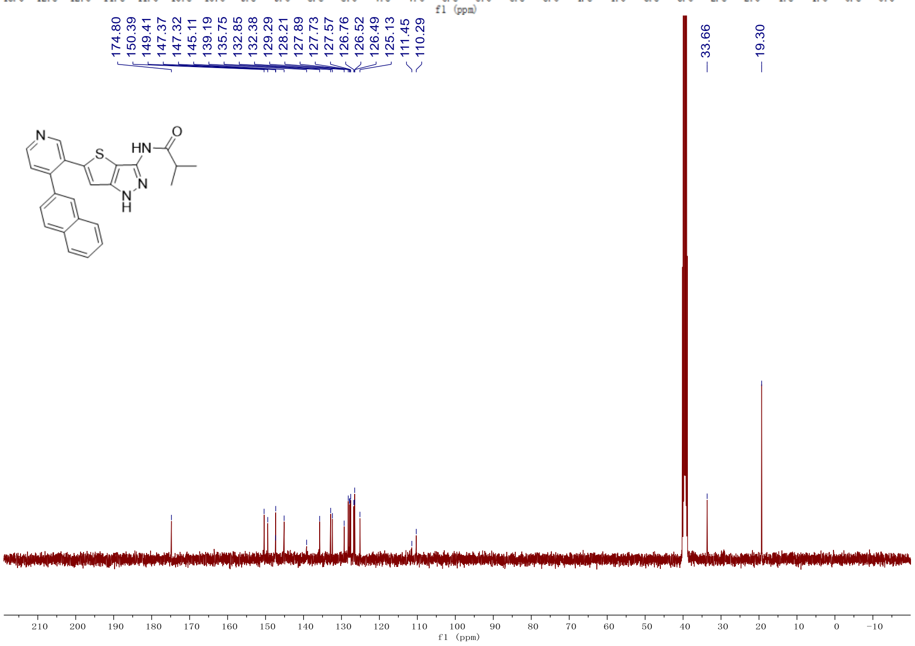

<Chromatogram>

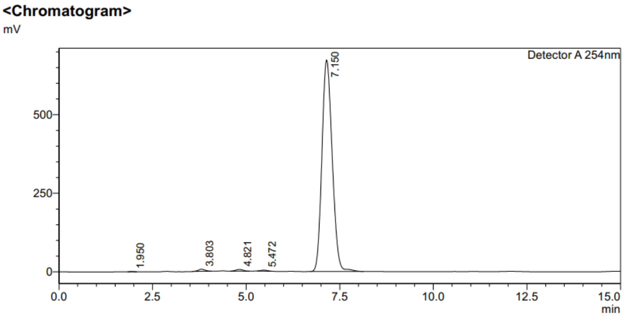

<Peak Table>

Detector A 254nm

Peak#	Ret. Time	Area	Height	Conc.	Unit
1	1.950	8226	1066	0.063	
2	3.803	90158	6960	0.689	
3	4.821	77621	5629	0.593	
4	5.472	40456	3142	0.309	
5	7.150	12863068	672858	98.345	
Total		13079529	689655		
